# Comparative genomics of the Natural Killer Complex in carnivores

**DOI:** 10.3389/fimmu.2024.1459122

**Published:** 2024-10-03

**Authors:** Jan Futas, April L. Jelinek, Pamela A. Burger, Petr Horin

**Affiliations:** ^1^ Research Group Animal Immunogenomics, Central European Institute of Technology (CEITEC) VETUNI, Brno, Czechia; ^2^ Department of Animal Genetics, Faculty of Veterinary Medicine, University of Veterinary Sciences Brno (VETUNI), Brno, Czechia; ^3^ Research Institute of Wildlife Ecology, University of Veterinary Medicine Vienna (VETMEDUNI), Vienna, Austria

**Keywords:** *CLEC*, *KLR*, Natural Killer Complex, carnivore, Felids, genomes

## Abstract

**Background:**

The mammalian Natural Killer Complex (NKC) harbors genes and gene families encoding a variety of C-type lectin-like proteins expressed on various immune cells. The NKC is a complex genomic region well-characterized in mice, humans and domestic animals. The major limitations of automatic annotation of the NKC in non-model animals include short-read based sequencing, methods of assembling highly homologous and repetitive sequences, orthologues missing from reference databases and weak expression. In this situation, manual annotations of complex genomic regions are necessary.

**Methods:**

This study presents a manual annotation of the genomic structure of the NKC region in a high-quality reference genome of the domestic cat and compares it with other felid species and with representatives of other carnivore families. Reference genomes of Carnivora, irrespective of sequencing and assembly methods, were screened by BLAST to retrieve information on their killer cell lectin-like receptor (KLR) gene content. Phylogenetic analysis of *in silico* translated proteins of expanded subfamilies was carried out.

**Results:**

The overall genomic structure of the NKC in Carnivora is rather conservative in terms of its C-type lectin receptor gene content. A novel KLRH-like gene subfamily (KLRL) was identified in all Carnivora and a novel KLRJ-like gene was annotated in the Mustelidae. In all six families studied, one subfamily (KLRC) expanded and experienced pseudogenization. The KLRH gene subfamily expanded in all carnivore families except the Canidae. The KLRL gene subfamily expanded in carnivore families except the Felidae and Canidae, and in the Canidae it eroded to fragments.

**Conclusions:**

Knowledge of the genomic structure and gene content of the NKC region is a prerequisite for accurate annotations of newly sequenced genomes, especially of endangered wildlife species. Identification of expressed genes, pseudogenes and gene fragments in the context of expanded gene families would allow the assessment of functionally important variability in particular species.

## 1 Introduction

Natural killer (NK) cells constitute a heterogeneous lymphocyte population (1). Conventional NK (cNK) cells found in the peripheral blood and spleen are endowed with potent cytotoxic capacity. They are primarily involved in innate immune responses against intracellular pathogens and tumor cells and contribute to the recognition of allogeneic cells. They also influence adaptive immune responses via the production of inflammatory cytokines (2) and crosstalk with dendritic cells (3). Tissue-resident NK (trNK) cells present in the liver, skin, kidney and virgin uterus (4) represent different NK lineages. Both uterine trNK and cNK cells orchestrate the implantation of semi-allogeneic embryo(s) and successful pregnancy in mice (5) and humans (6).

The diversity of the NK cell receptor repertoire represented by inherited haplotypes of NK cell receptor genes is essential for multiple NK cell functions. Stochastic expression of NK cell receptors on individual NK cells results in different sets of inhibitory and activation receptors on their surface. NK cell inhibitory receptors signal via cytoplasmic immunoreceptor tyrosine-based inhibitory motifs (ITIM) that recruit tyrosine phosphatase SHP1. Contrarily, activation receptors mostly couple through a cationic amino acid in their transmembrane domain to molecules containing immunoreceptor tyrosine-based activation motifs (ITAM), e.g. adaptor proteins CD3ζ, FcϵRIγ, DAP10 or DAP12. Integration of activating and inhibitory signals originating from various surface receptors determines the activation status of an individual NK cell, providing the capacity to discriminate between self and non-self or altered self (7).

The mechanism of missing-self recognition is based on the capacity of inhibitory NK receptors to bind MHC class I molecules on target cells, preventing NK cell degranulation and overriding activation signals mediated by different ligands. The molecules recognizing MHC class I ligands are killer cell lectin-like receptors (KLR) Ly49 in mice and killer cell immunoglobulin-like receptors (KIR) in humans. Both of these show high allelic and haplotypic variability in individuals. Activating members of these gene families binding MHC class I molecules are crucial for successful pregnancy, as exemplified by the differential outcomes of various combinations of maternal KIR haplotypes and fetal HLA-C2 in humans (8) and by a Ly49 knock down model in mice (9). The mechanism of altered-self recognition relies on different activating NK receptors which, upon binding various stress-induced ligands expressed on target cells, can override inhibitory signals and activate NK cells.

The natural killer gene complex (NKC) is located on mouse chromosome 6 and human chromosome 12 and contains a number of C-type lectin-like receptor (CTLR) genes. The mouse and human NKC genomic regions have been well characterized (10). NKC genes have been classified as ‘killer cell lectin-like receptor’ (*KLR*) genes expressed by NK cells and ‘C-type lectin receptor’(*CLEC*) genes expressed on myeloid cells. Eleven sub-families/loci of *KLR* genes are currently recognized in mammals (*KLRA*-*KLRK*). Within the mouse NKC, genes encoding *CLEC4* receptors which bind carbohydrates in the presence of Ca^2+^ ions constitute a gene family located between the *KLRG* and *KLRB* loci. The *KLRG1* gene has been relocated in the human NKC. It encodes an inhibitory homodimeric receptor for cadherin molecules in the mouse and humans (11). Genes of the *KLRB* and/or *KLRF* family are intercalated with genes of the *CLEC2* family, thus forming pairs of genetically linked receptors and ligands, respectively (12). These are followed by the genes of the Dectin-1 cluster. The functions and known ligands of myeloid receptors were reviewed by Scur et al. (13). The *KLRE* gene is adjacent to this cluster in the mouse, but not in the human NKC. Genes of the *KLRE* and *KLRI* loci, which code for heterodimeric receptors in the mouse and rat (14), have also been identified in even-toed ungulates (15, 16). The *KLRD* gene codes for the CD94 protein, one chain of the heterodimeric receptors for non-classical MHC class I molecules Qa-1(b) in mice (17) and HLA-E in humans (18). The second chain of these receptors is encoded by genes of the *KLRC* family. *KLRC1* codes for an inhibitory receptor with two ITIM motifs (NKG2A) (19); *KLRC2* encodes an activating receptor (NKG2C) with arginine in the transmembrane domain (20). The *KLRK* gene encodes an homodimeric activating receptor NKG2D for diverse stress ligands in humans (MICA, MICB, ULBL1-6) and mice (H60, Rae1, Mult1) as reviewed by Lanier (21). In rats, *KLRH1* codes for a homodimeric inhibitory receptor recognizing MHC class I ligands (22); however, the mouse reference genome contains a *KLRH1* gene with a mutated ITIM. The *KLRH* gene family was identified in ungulates with a varying extent of pseudogenization (15) as well as in rabbits (23). In rodents, the *KLRA* (Ly49) genes encode homodimeric receptors for MHC class I molecules. They have expanded into a huge family containing both inhibitory and activating receptors (24). Similarly, the horse (25) and equids (26) possess at least five functional *KLRA* genes. The human *KLRA* counterpart is a pseudogene. It marks the boundary of the NKC.

The organization of the NKC in the draft genomes available to date of species other than rodent or human was compared by Hao et al. (27). Both expansion and reduction of gene numbers was observed for some gene families in cattle and dogs. Although mRNA for bovine *KLRJ* was described (28), its product has not yet been characterized as a receptor or co-receptor. A more comprehensive comparison between KLR gene clusters located within the NKCs of ruminants (cow, goat, sheep), ungulates (pig, horse), lemurs, rats and humans was made by Schwartz et al. (15). Long-read assemblies of cow and goat genomes made a detailed characterization of their expanded *KLRC* and *KLRH* genes possible. An outline of the NKC evolution in mammals documenting the plasticity of the KLR genes was presented by Hilton et al. (23).

The accuracy of the annotation of complex genomic regions such as the Leukocyte Receptor Complex (LRC), NKC and MHC is limited by the methods used for whole genome sequencing. Automatic annotation of genomes has proved to be insufficient for immune genes in non-model species (59% inaccurately annotated and 21% of genes not annotated) due to poor support of gene models by RNA-sequencing data and to genes missing in model databases (29). Manual annotations based on long contigs scaffolded with Hi-C may lead to a near-complete reconstruction of immune gene clusters. As such, they represent the approach of choice for complex regions of non-model species (29). Recently, we compared the LRC regions of carnivores (30). Here, we made a similar comparison and evolutionary analysis for the carnivore NKC with special focus on the KLR genes that represent the most variable part of the mammalian NKC.

## 2 Methods

### 2.1 Genomic resources

Eighty-nine NCBI reference or other genomes of 74 carnivore species were analyzed (https://www.ncbi.nlm.nih.gov/genome). A full list of these along with corresponding links and abbreviations used throughout the text is provided in **Supplementary Table 1**. Families of carnivores were represented as follows: Felidae (22 species), Viverridae (1 species), Canidae (14 species), Mustelidae (19 species), Otariidae (4 species), Phocidae (8 species) and Ursidae (6 species). Short-read based genomes of three Feliformia families (Eupleridae, Herpestidae, and Hyaenidae) and four Caniformia families (Ailuridae, Mephitidae, Odobenidae, and Procyonidae) were excluded because their fragmented genes hampered the analysis.

### 2.2 Annotations

We followed the nomenclature rules of the HUGO Gene Nomenclature Committee for orthologues in humans (https://www.genenames.org/) (**Table 1**) and VGNC for the cat and dog (https://vertebrates.genenames.org/). According to rules suggested for KLR genes in non-model animals (15), numbers should be assigned to genes and pseudogenes consecutively from centromere to telomere. Our numbering of genes in expanded gene families is provisional. Two categories of pseudogenes were distinguished: the full-length genes with a 1-2 bp insertion/deletion or premature stop codon and obvious pseudogenes with frameshift mutations or missing parts. For the sake of clarity, numbers were given only to full length genes identified between chosen framing genes, ordered from the mannose-6-phosphate receptor gene (*M6PR*) to the protein mago nashi homolog 2 (*MAGOHB*), irrespective of the centromere to telomere orientation of the NKC. As usual in mammals, *KLRC1* denotes inhibitory receptors, while *KLRC2* designates activating receptors. A new category (*KLRC3*) was introduced for dual purpose receptors containing both inhibitory and activating signaling motifs. Likewise, a new name was introduced for *KLRH-like* genes: *KLRL*. The acronym *KLRL1* was already used in two papers (31, 32). However, according to the current nomenclature, the name *CLEC12A* is used for the corresponding gene in humans and mice. Therefore, we prefer *KLRL* to the next acronym *KLRM* for this newly identified gene family in carnivores.

**Table 1:** Comparison of CTLRs of mouse and human NKC.

Locus	Mouse proteins	Human proteins	Signaling motif
*KLRG*	Klrg1	MAFA	ITIM
*CLEC4*	Dcar, Dcar1 – Dcar4, Dectin-2, Mcl, Mincle	BDCA2, DCIR, DECTIN-2, MCL, MINCLE	ITAM/ITIM
*KLRB*	Nkrp1-a – Nkrp1-g	NKR-P1A	ITIM/arginine
*CLEC2*	Clr-a – Clr-h, Cd69	AICL, KACL, LLT1, CD69	
*KLRF*	Not applicable	NKp80, NKp65	hemITAM
*CLEC12*	Micl, Clec12b	MICL (KLRL1), CLEC12-B	ITIM
*CLEC1*	Clec-2, Clec-1	CLEC-2	hemITAM
*CLEC9*	Dngr-1	DNGR1	hemITAM
*CLEC7*	Dectin-1	DECTIN-1	hemITAM
*OLR1*	Lox-1	LOX-1	
*KLRE*	Nkg2i	Not applicable	
*KLRD*	Cd94	CD94	
*KLRK*	Nkg2d	NKG2D	arginine
*KLRC*	Nkg2a/b, Nkg2c, Nkg2e	NKG2A, B, C, E	ITIM/arginine
*KLRI*	Klri1, Klri2	Not applicable	ITIM/lysine
*KLRH*	Klrh1	Not applicable	(ITIM)
*KLRA*	Ly49a-Ly49q	(pseudogene)	ITIM/arginine

The presence of NKC genes was assessed in the NCBI genome annotations of the highly contiguous genomes of five Carnivora representatives: domestic cat (*Felis catus*, GCF_018350175.1), ermine (*Mustela erminea*, GCF_009829155.1), California sea lion (*Zalophus californianus*, GCF_009762305.2), northern elephant seal (*Mirounga angustirostris*, GCF_021288785.2), and brown bear (*Ursus arctos*, GCF_023065955.2). Model mRNA sequences of full length proteins were retrieved for each species and compared across the entire group using the NCBI Splign tool (33) (stopped in March 2024, https://www.ncbi.nlm.nih.gov/sutils/splign/splign_note.html) against the NKC regions of other species. For genomes not annotated by NCBI, the annotation was made by comparison with closely related species. The grey wolf NKC (*Canis lupus*, GCA_905319855.2) was annotated based on the dog NCBI RefSeq annotation (GCF_014441545.1). Similarly, the masked palm civet NKC (*Paguma larvata*, GCA_030068075.1) was annotated based on the domestic cat reference genome. Corrected annotations of seven genomic regions between the *M6PR* and *MAGOHB* genes are provided in **Supplementary Table 2**. Coding sequences (CDS) of CTLR genes are listed in **Supplementary Data Sheet 1**. Typically, CTLRs are type II transmembrane glycoproteins with an N-terminal cytoplasmic tail, a transmembrane domain, a stalk and a single extracellular C-type lectin-like domain (CTLD). Transmembrane domains were checked by DeepTMHMM prediction (https://dtu.biolib.com/DeepTMHMM) and CTLD domains were checked by NCBI Conserved Domain Search on presumed protein sequences (https://www.ncbi.nlm.nih.gov/Structure/cdd/wrpsb.cgi).

To mine data from Carnivora genomes regarding *KLR* gene content, high quality mRNA models of KLR sequences were used to BLAST^®^ search (https://blast.ncbi.nlm.nih.gov/Blast.cgi?PROGRAM=blastn&PAGE_TYPE=BlastSearch&LINK_LOC=blasthome) other genomes within the relevant family to retrieve fragments with similar sequences. These BLAST hits were identified by Splign tool alignment. The coding sequences of full length genes were extracted to BioEdit version 7.2.6 (34) and are available in FASTA format in **Supplementary Data Sheet 2**. VISTA comparisons (https://genome.lbl.gov/vista/index.shtml) were used for uncertain sequences/regions identified during manual annotation.

### 2.3 Phylogenetic analysis

The phylogeny of Carnivora CTLR genes was reconstructed using MEGA X version 11.0.13 (35). Sequences were aligned using the Multiple Sequence Comparison by Log-Expectation (MUSCLE) algorithm as implemented in the software. The Neighbor-Joining method was used both for nucleotide (CDS, p-distance) and protein sequences (p-distance) with 1000 bootstrap replicates to construct phylogenetic trees. A set of murine reference genes was used as an outgroup; their CDS are available in **Supplementary Data Sheet 1**.

## 3 Results

### 3.1 Structure of the NKC genomic region in carnivores

**Figure 1:**
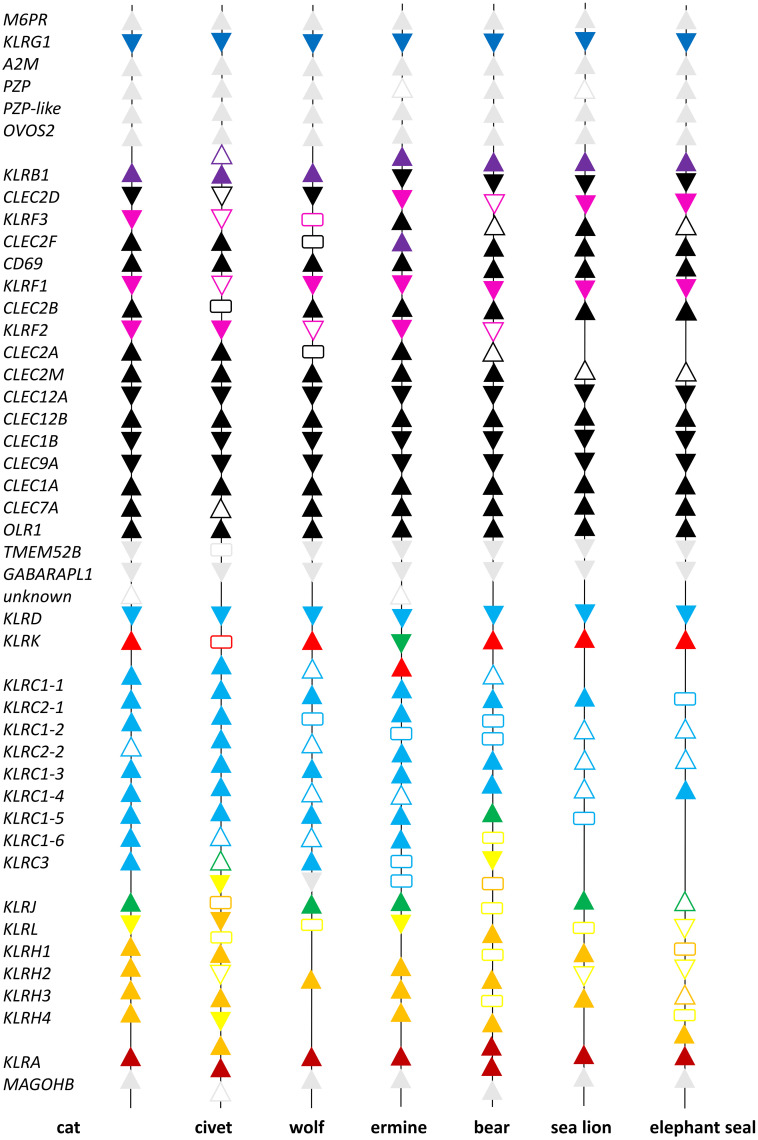
Comparison of the Natural Killer Complex structure in carnivores. Seven representatives of Carnivora are depicted: the domestic cat (*Felis catus*), masked palm civet (*Paguma larvata*), gray wolf (*Canis lupus*), ermine (*Mustela erminea*), brown bear (*Ursus arctos*), California sea lion (*Zalophus californianus*) and northern elephant seal (*Mirunga angustirostris*). Individual NCBI reference genome sequences of the NKC were manually annotated based on cross-species comparisons. The scheme is not to scale, functional genes are represented by *solid color triangles* pointing in the direction of transcription, full length pseudogenes by *open triangles* and fragments of genes by *rectangles*. Color scheme: grey - framing genes; black - *CLEC* genes; blue - *KLRG*; violet - *KLRB*; magenta - *KLRF*; cyan - *KLRD* and *KLRC*; red - *KLRK*; green - *KLRJ*; yellow - *KLRL*; orange - *KLRH*; and brown - *KLRA* genes. For details on gene names see **Supplementary Table 2**.

A comparison of the NKCs of seven representatives of carnivores can be found in **Figure 1**. Details concerning the names of genes and mRNA models used are summarized in **Supplementary Table 2**. Based on these data, the NKC of carnivores can be sub-divided into two parts.

**Figure 2:**
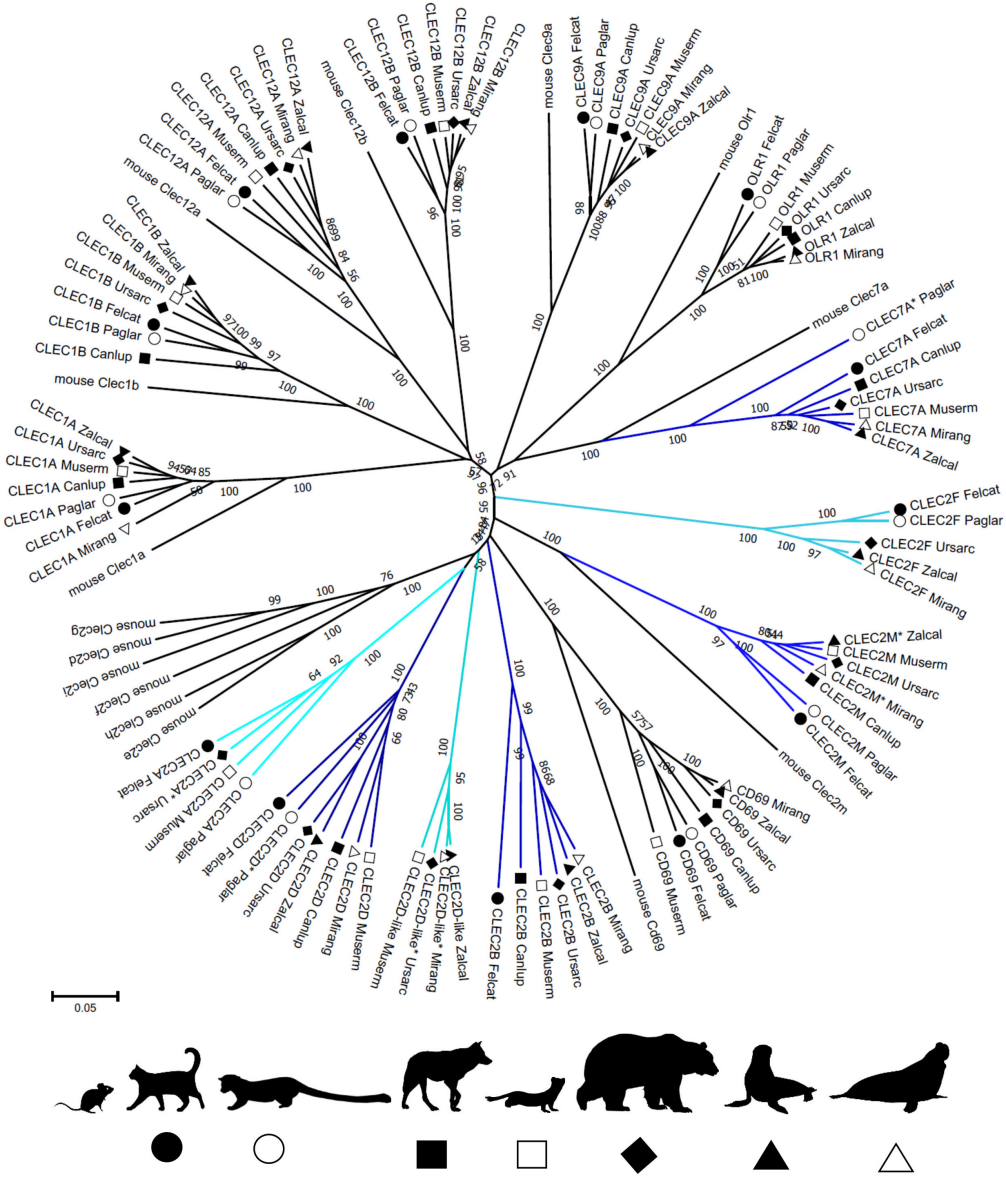
Phylogeny of CLEC genes in the NKC of Carnivora. Nucleotide coding sequences of putatively functional genes and full length pseudogenes (*) from the domestic cat (*Felcat*), masked palm civet (*Paglar*), gray wolf (*Canlup*), ermine (*Muserm*), brown bear (*Ursarc*), California sea lion (*Zalcal*) and northern elephant seal (*Mirang*) were compared to murine CLECs (**Supplementary Data Sheet 1**) by the Neighbor-Joining method and p-distance model in MEGA X. A tree is presented with branches reproduced in over 50% of 1000 replicates. Genes not having a functional homolog in all Carnivora representatives are highlighted in hues of blue. Silhouettes were adopted from Silhouette Image Collection - PhyloPic.

The first part, delimited by *M6PR* and *TMEM52B*, comprises mostly CLEC genes (**Figure 1**). It is rather conservative in terms of the number of genes. The few exceptions are mainly KLR genes (*KLRB* and *KLRF*) and *CLEC2* genes. Eleven CLEC genes were found in all species and their orthologues/homologues are present in mice (**Figure 2**). Three *CLEC2* genes (*CLEC2A*, *CLEC2D-like* and *CLEC2F*) were found only in some of the species analyzed. *KLRG* is a single gene in all species except the masked palm civet, in which a processed pseudogene was identified on the chromosome containing the NKC. The *KLRB* gene is present as a single gene in all species except the masked palm civet and ermine, in which subfamilies consisting of two genes were identified. The *KLRF* subfamily of three genes (*KLRF1*, *KLRF2* and *KLRF3*) was identified in all species except marine carnivores. The *KLRF2* gene was not found in the sea lion and elephant seal.

**Figure 3:**
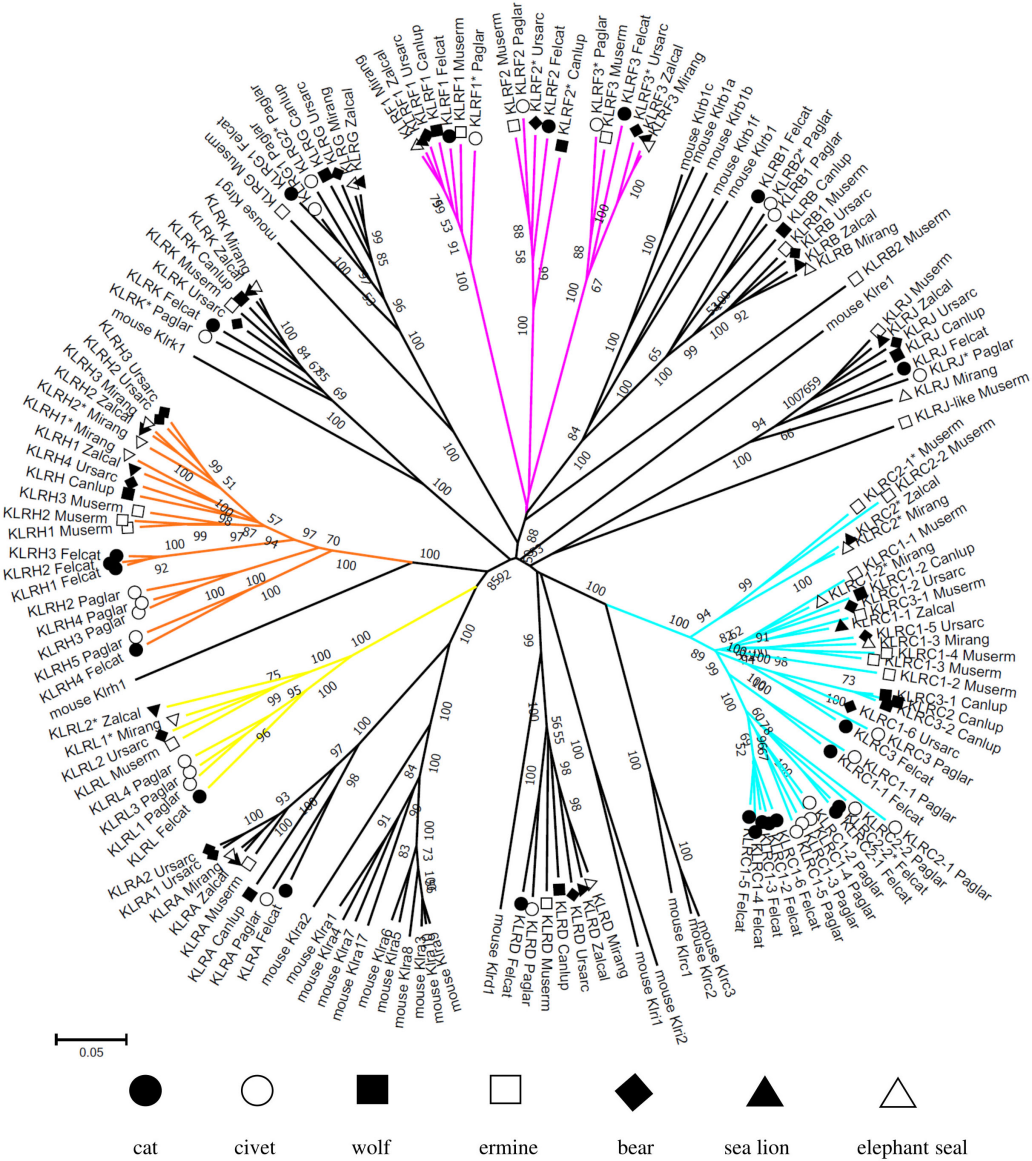
Phylogeny of KLR genes in Carnivora. Nucleotide coding sequences of putatively functional genes and pseudogenes (*) from the domestic cat (*Felcat*), masked palm civet (*Paglar*), gray wolf (*Canlup*), ermine (*Muserm*), brown bear (*Ursarc*), California sea lion (*Zalcal*) and northern elephant seal (*Mirang*) were compared to murine KLRs (**Supplementary Data Sheet 1**) by the Neighbor-Joining method and p-distance model in MEGA X. A tree is presented with branches reproduced in over 50% of 1000 replicates. KLR subfamilies with expansion/retraction of genes in more than two carnivores are highlighted: *KLRC* (*cyan*), *KLRF* (*magenta*), *KLRH* (*orange*) and *KLRL* (*yellow*).

The second part of the NKC of carnivores is delimited by *GABARAPL1* and *MAGOHB* (**Figure 1**). It comprises four KLR subfamilies, *KLRA*, *KLRC*, *KLRH* and *KLRL* (a novel *KLRH-like* subfamily), with variable numbers of genes. The *KLRD*, *KLRK* and *KLRJ* genes are present as single genes in all species. A *KLRJ-like* gene was identified in the ermine (**Figure 3**).

The phylogenetic reconstruction of the coding sequences of CLEC genes (**Figure 2**) shows rather conservative evolution and no gene was identified as arising from a recent duplication event. The phylogenetic tree for KLR genes (**Figure 3**) presents different evolutionary paths for individual lineages of KLR genes. While there are some conserved single genes in all seven species (*KLRD, KLRG, KLRJ* and *KLRK)* or with duplication in one species (*KLRA* and *KLRB*), the duplications of *KLRF* gene must have occurred in a common ancestor of Feliformia and Caniformia. The lineages of the *KLRC*, *KLRH* and *KLRL* families document relatively recent duplication events for some of their genes with species-specific amplification.

### 3.2 The KLR gene contents of the NKC in carnivores

**Figure 4:**
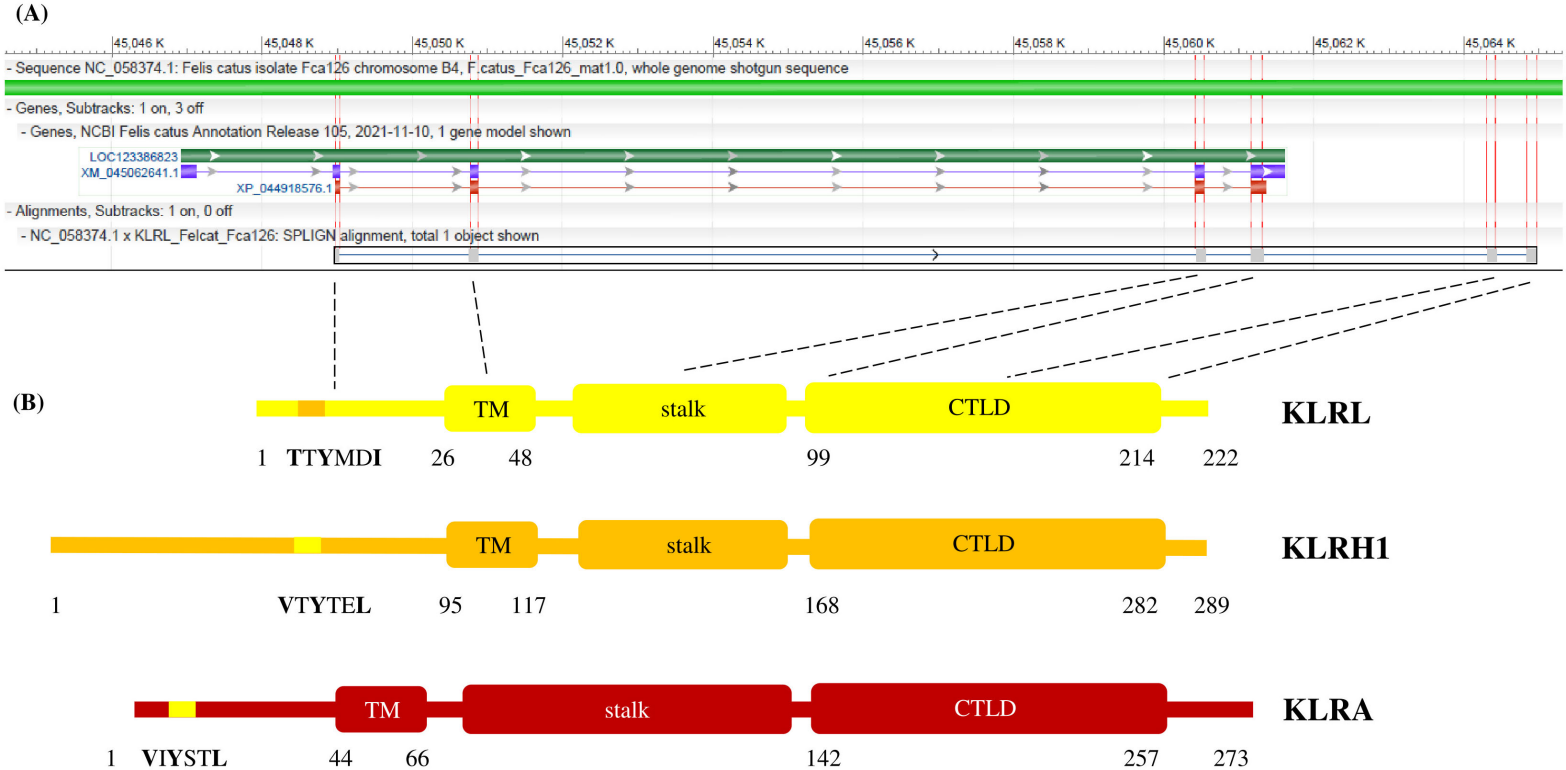
Structures of KLR genes and a corresponding polypeptide chain. **(A)** The NCBI annotation of the *KLRL* locus (LOC123386823, *green*) from the domestic cat reference genome (*Felis catus* NC_058374.1). Locus spans approx. 14 kb on chromosome B4 (between 45,046 kb and 45,062 kb) and is annotated with an mRNA model (XM_045062641.1, *blue*) and a protein model (XP_044918576.1, *red*), both missing a part of the functional gene. Comparison is made with the manual annotation based on Splign alignment of the corrected CDS model (KLRL_Felcat_Fca126, *grey*) obtained by cross-species Splign of Carnivora sequences. Exons are highlighted (*red vertical bars*) and their content is related to the polypeptide chain (*dashed black lines*); the 3´-untraslanted region is not shown. The image was created using NCBI Genome Workbench version 3.9.1 (http://www.ncbi.nlm.nih.gov/tools/gbench/, retired March 2024). **(B)** The predicted polypeptide chain of feline KLRL (222 amino acids) is compared to the closely related KLRH1 (289 amino acids) and to KLRA (273 amino acids). All three proteins represent the typical organization of a KLR monomer: cytoplasmic tail with a signaling motif; transmembrane domain (TM); stalk region; and C-type lectin-like domain (CTLD). KLRL contains an immunoreceptor tyrosine-based switch motif (with TTYMDI amino acid sequence), KLRH1 (VTYTEL) and KLRA (VIYSTL) possess an immunoreceptor tyrosine-based inhibitory motif each. Numbers denote the boundaries of the respective elements.

The structure of a KLR gene typically involves the 5´-untranslated region, an exon coding for the cytoplasmic tail, an exon for the transmembrane domain, an exon for the stalk region and three exons for the CTLD, accompanied by the 3´-untranslated region. The feline *KLRL* (**Figure 4A**) provides an example of the corrected CDS for a gene based on cross-species comparisons. The organization of its polypeptide was compared to feline KLRH1 and KLRA (**Figure 4B**).

A full set of model mRNA sequences of KLR genes was searched against the genomes of six carnivore families. The numbers of genes (full length and pseudogenes) found are summarized in **Table 2**. Individual species are described in **Supplementary Table 3**. The organization of the KLR sub-regions of the NKC in carnivores is compared in **Supplementary Figures 1**-**6**.

**Table 2:** Complement of KLR genes/pseudogenes in carnivores.

Family (number of species)	*KLRA*	*KLRB*	*KLRC*	*KLRD*	*KLRF*	*KLRG*	*KLRH*	*KLRJ*	*KLRK*	*KLRL*
Felidae (22)	1	1	7-19	1	3	1	3-26	1	1	1
Canidae (14)	1	1	4-11	1	3	1	1-2	1	1	1
Mustelidae (19)	1	2	8-13	1	2-3	1	1-8	2	1	1-2
Otariidae (4)	1	1	4-5	1	2	1	2	1	1	1-2
Phocidae (8)	1	1	4-5	1	2	1	3-5	1	1	3-4
Ursidae (6)	0-3	1	6	0-1	3	1	1-4	1	1	2-5

#### 3.2.1 Felidae

The structure of the NKC of the Felidae is the most variable among carnivores. Their NKC is characterized by single *KLRA*, *KLRB*, *KLRD*, *KLRG*, *KLRJ*, *KLRK* and *KLRL* genes, three *KLRF* and various numbers of functional *KLRC* and *KLRH* genes (**Supplementary Figure 1**). One dual-purpose receptor, *KLRC3*, is found in all Felid genomes along with five to eleven predominantly inhibitory *KLRC* genes. Likewise, one activating *KLRH* in all Felids is accompanied by one to four *KLRH* genes for activating and/or inhibitory receptors.

#### 3.2.2 Canidae

Low numbers and low variability of functional KLR genes were observed in the NKC genomic region of dogs and wolfs. In addition to single *KLRA*, *KLRB*, *KLRD*, *KLRF1*, *KLRG*, *KLRH*, *KLRJ* and *KLRK* genes, the region contains three to four *KLRC* genes. In some assemblies, one inhibitory and two dual-purpose receptors are accompanied by another activating receptor. Foxes possess a very similar NKC with two to three *KLRC* genes (**Supplementary Figure 2**). The red fox and Corsac fox have two functional *KLRF* genes (*KLRF1* and *KLRF3*).

#### 3.2.3 Mustelidae

Mustelids have the most complicated and diverse structure of the NKC among Caniformia (**Supplementary Figure 3**). Single genes of *KLRA*, *KLRD*, *KLRG*, *KLRK* and *KLRL* are accompanied by two *KLRB*, three *KLRF* and two *KLRJ* genes (*KLRJ* and *KLRJ-like*). Four to six *KLRC* genes encoding various types of receptors (KLRC1, KLRC2, KLRC3) are complemented by one to four *KLRH* with activating, inhibitory or both signaling motifs.

#### 3.2.4 Otariidae

The genomes of the Guadalupe fur seal, the northern fur seal, the Steller sea lion, and the California sea lion contain the smallest numbers of functional KLR genes among carnivores. Except for the Guadalupe fur seal, all possess *KLRA*, *KLRB*, *KLRD* with inhibitory *KLRC1* (CD94/NKG2), *KLRG*, *KLRJ*, and *KLRK* accompanied by two inhibitory *KLRH* genes (**Supplementary Figure 4**). *KLRK* and *KLRH2* are missing in the Guadalupe fur seal. Two functional *KLRF* genes (*KLRF1* and *KLRF3*) were found in all but the northern fur seal (*KLRF1* only).

#### 3.2.5 Phocidae

The gray seal, the Weddell seal, the northern elephant seal, the southern elephant seal, the Hawaiian monk seal, the harbor seal, the Saimaa ringed seal, and the Baikal seal possess a set of functional KLR genes comparable to Otariidae along with a putative *KLRL* gene without a signaling motif (**Supplementary Figure 5**). Their functional inhibitory *KLRC1* is a different gene from the gene found in the Otariidae. The genomes of the Hawaiian monk seal, the harbor seal, and the Saimaa ringed seal have an additional inhibitory *KLRH* gene (three *KLRH* total). The Weddell seal and Hawaiian monk seal have only one functional *KLRF* gene (*KLRF1*).

#### 3.2.6 Ursidae

The giant panda, the Malayan sun bear, the spectacled bear, the American black bear, the brown bear, and the polar bear are a more diverse group in terms of their NKC compared to marine carnivores, but less variable in comparison to mustelids. The *Ursus* species all possess the same set of functional KLR genes (two inhibitory *KLRA*, single *KLRB*, *KLRD* with three inhibitory *KLRC1* (CD94/NKG2), *KLRF1*, *KLRG*, two inhibitory *KLRH, KLRJ*, *KLRK*, and *KLRL*) with the exception of one activating *KLRH* which is functional in the brown bear and the polar bear but pseudogenized in the American black bear (**Supplementary Figure 6**). Although probably improperly assembled, the NKC of the Malayan sun bear seems to be similar to the NKC of the spectacled bear with one *KLRB*, *KLRD* with four inhibitory *KLRC1*, *KLRF1*, *KLRG*, one inhibitory *KLRH*, *KLRJ*, *KLRK*, and *KLRL*. The giant panda reference genome also shows similarities to the spectacled bear NKC, although its *KLRH* is activating and functional *KLRA* has both inhibitory and activating signaling motifs (an ITIM and a cationic amino acid in the transmembrane domain). However, another giant panda genome differs from the reference genome by three *KLRA* genes (one activating, two inhibitory) and a stop codon in *KLRL2.*

### 3.3 Phylogeny of KLR receptors

**Figure 5:**
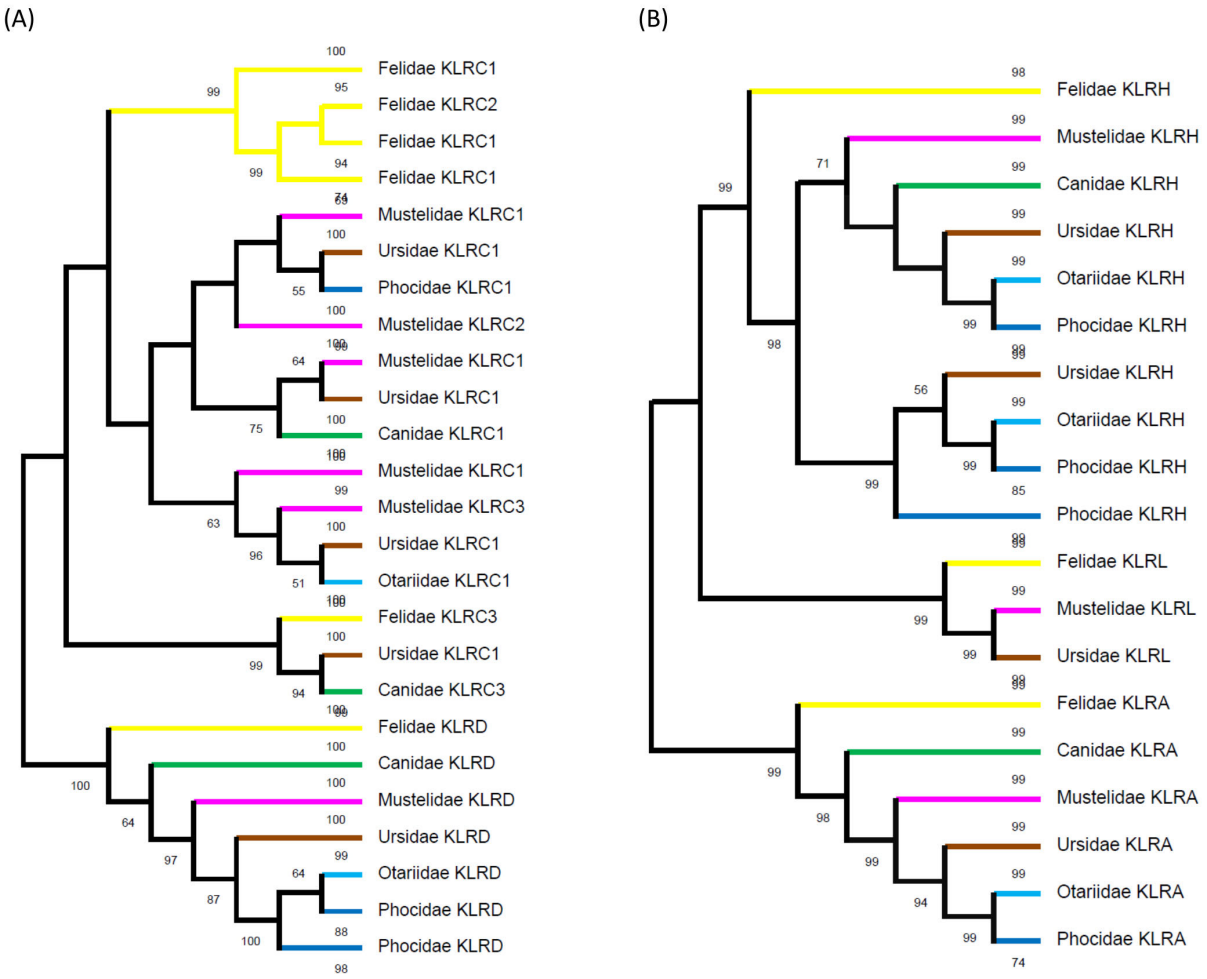
Phylogeny of expanded lineages of KLR receptors. **(A)** Overview of the phylogenetic tree for KLRC proteins identified in families of Carnivora related to co-receptor KLRD amino acid sequences. While the KLRD lineage is conserved, the KLRC lineages split in ancestors of Caniformia and Feliformia independently. The KLRC3 lineage was present in a common ancestor of Carnivora. For details see **Supplementary Figure 7**. **(B)** Simplified view of the phylogenetic tree for KLRH and KLRL proteins identified in families of Carnivora related to KLRA amino acid sequences. KLRA and KLRL lineages were present in a common ancestor of Carnivora. The KLRH lineage split in ancestors of Caniformia and the expansion of KLRH receptors seen in Felidae is family-specific. For details see **Supplementary Figure 8**.

A comparison of the phylogenetic tree for KLRA, KLRH and KLRL to the tree for KLRC and KLRD protein sequences identified in Carnivora families is depicted in **Figure 5**.

A phylogenetic reconstruction of the expanded KLRC was compared to the KLRD co-receptor proteins (for details see **Supplementary Figure 7**). While it suggests conservative evolution of KLRD from a common ancestor in all six carnivore families, a family-specific expansion of KLRC genes (except KLRC3) occurred in the Felidae. It seems that feline KLRC3 probably has a common ancestor with both KLRC3 and KLRC2 of Canidae and KLRC1-6 of Ursidae. There appear to be at least three other KLRC lineages in the common ancestor of the Caniformia. Canidae, Otariidae and Phocidae have retained only one lineage each, Ursidae and Mustelidae have retained all three lineages, and in Mustelidae duplications have occurred in two of the lineages.

Likewise, the expanded KLRH proteins were compared to the KLRA and KLRL proteins (for details see **Supplementary Figure 8**). Comparable to KLRA in all six families, the KLRL protein in the Felidae, Mustelidae and Ursidae can be traced back to a common ancestor of carnivores. The KLRH amplifications in the Felidae and Mustelidae are family-specific, while there were probably two lineages of KLRH in a common ancestor of other Caniformia. The Canidae have retained only one lineage, but both lineages remain in the Otariidae. The Ursidae and Phocidae experienced duplication in one of the lineages.

### 3.4 Signaling motifs of carnivore KLR receptors

**Figure 6:**
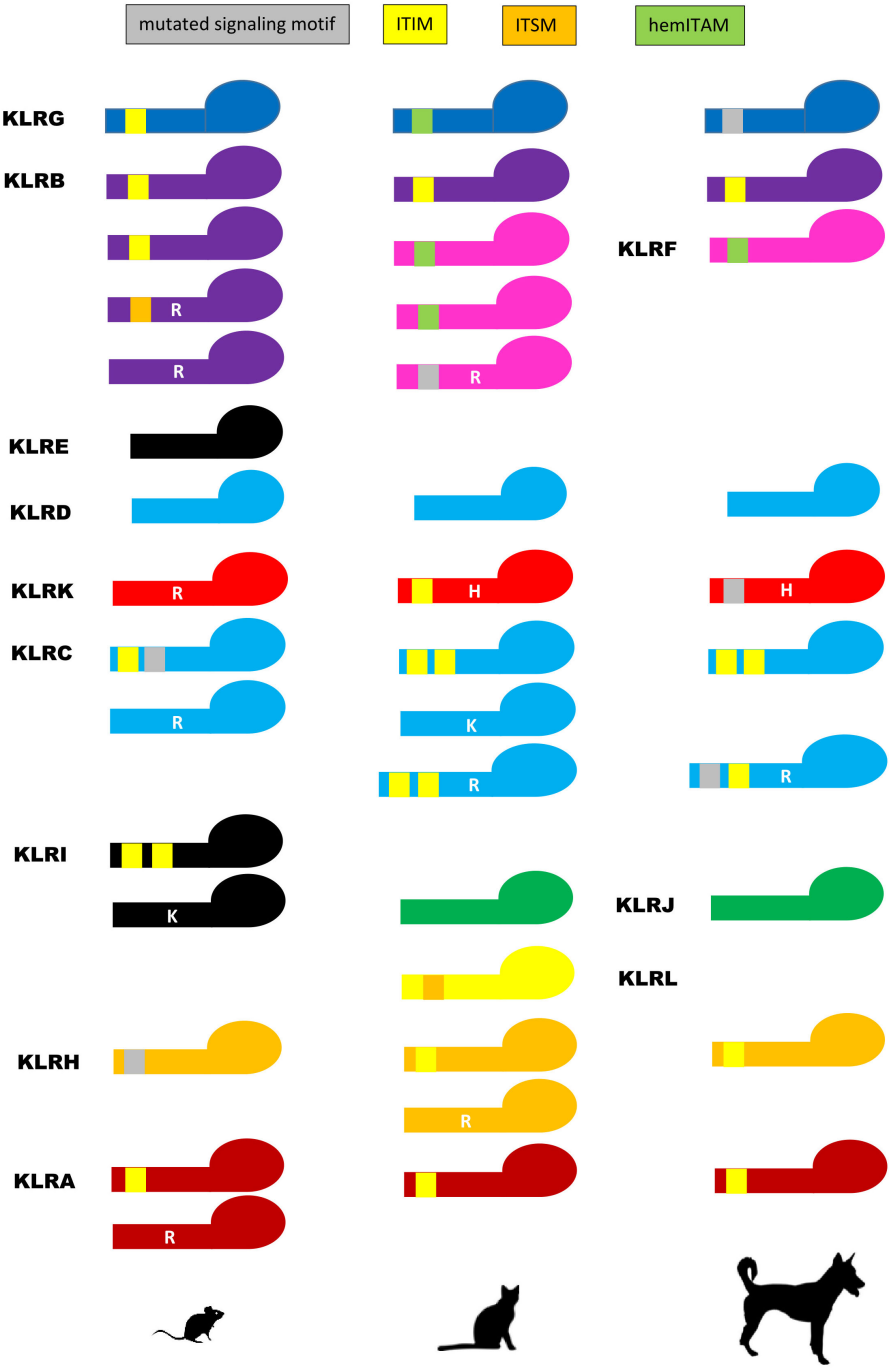
Signaling motifs of KLR proteins. Various signaling motifs identified in the polypeptide chain sequences of KLR receptors of the domestic cat (*Felis catus*) and the dog (*Canis lupus familiaris*) are compared to murine (*Mus musculus*) KLR monomers. The immunoreceptor tyrosine-based inhibitory motif (ITIM, *yellow rectangle*), the immunoreceptor tyrosine-based switch motif (ITSM, *orange rectangle*) and the hemi-immunoreceptor tyrosine-based activation motif (hemITAM, *green rectangle*) were recognized if conforming to consensus amino acid sequences: ITIM – I/V/LxYxxI/V/L; ITSM – S/TxYxxI/L; hemITAM – DEDGYxxL. Mutated signaling motifs are depicted as *grey rectangles*. A cationic amino acid in the transmembrane domain conferring an activation signal is depicted by a *white capital letter* (arginine – R, histidine – H, lysine – K). Color coding for KLR receptor monomers follows the scheme used for *KLR* gene lineages in **Figure 1**. KLRE forms heterodimers with KLRI in mice. They are not present in Carnivora, and thus are given in *black*. Silhouettes were adopted from Silhouette Image Collection - PhyloPic.

A comparison of the signaling motifs of carnivore KLR receptors to their murine counterparts shows their common as well as contrasting features (**Figure 6**). There are three KLR lineages concordant with murine receptors. The KLRA receptors of carnivores possess canonical ITIMs like some of murine Klra (Ly49), but one receptor with a mutated ITIM and a transmembrane arginine was identified in the giant panda. The KLRB molecule of carnivores contains an ITIM in its cytoplasmic tail as seen in two of murine Klrb receptors. As in mice, KLRD contains no signaling motif in any of the carnivores.

Two KLR lineages are comparable to murine homologs. The two opposing murine Klrc receptors are mirrored in carnivores by an inhibitory KLRC1 containing two ITIM motifs and by activating KLRC2 with a cationic amino acid in the transmembrane domain. In those Caniformia with functional genes, the KLRC2 transmembrane domain contains an arginine, while all Felidae have a lysine in this domain. The existence of KLRC3 dual-purpose receptors with both ITIM(s) and a transmembrane arginine is specific to Carnivora. Comparably to rat Klrh with inhibitory function, the KLRH of Carnivora contain ITIM(s). But again, some dual purpose or activating receptors with an ITIM (or a mutated ITIM) and/or a transmembrane arginine were also found across carnivores.

Two lineages differ between carnivores and mice. In contrast to the murine (and human) ITIM-containing gene *Klrg1*, the KLRG molecule of the domestic cat possesses a signal resembling the hemi-immunoreceptor tyrosine-based activation motif (hemITAM) (36). This feature is common to all Felidae, Otariidae (except the northern fur seal KLRG which has an ITIM domain) and Phocidae. In the Ursidae, this motif differs (DDGIYSEL/DGGIYSEL), while in the Canidae and Mustelidae, only mutated motifs were identified. Likewise, the KLRKs of all Caniformia possess a histidine in the transmembrane region while the murine activating Klrk contains arginine. In all Felidae, this signaling motif is accompanied by an ITIM-like motif in the cytoplasmic tail of the molecule.

There are few lineages without homologs between mice and carnivores. KLRE and KLRI are not present in Carnivora. KLRF, KLRJ, KLRJ-like and KLRL can be found in carnivores but not in mice. The KLRF proteins are characterized by hemITAM motifs in all carnivores except KLRF3 which bears an arginine in its transmembrane region. KLRJ contains no signaling motif, contrary to the KLRJ-like of mustelids which bears an ITIM. The newly identified KLRL molecules have a predicted immunoreceptor tyrosine-based switch motif (ITSM) in all Felidae, but no signaling motif was identified in the Mustelidae or the Ursidae.

## 4 Discussion

The remarkable evolutionary plasticity of the NKC and LRC gene clusters has been established by previous studies. Even closely related species may differ markedly as a result of rapid, lineage-specific expansions or contractions of sets of loci (10). Therefore, interspecific extrapolations are of limited value and a detailed annotation of these complex regions for each individual non-model species is necessary, as demonstrated by this study of Carnivora.

The advantage of exploring genomes obtained by a ´kitchen sink approach´ to genome sequencing and assembly is that it allows the integration of information from a variety of different methods leading to a reconstruction of complex gene families such as the MHC, LRC, and NKC regions (29). This approach combines multiple methods (long-reads, short-reads, HiC scaffolding and optical mapping) and benefits from the complementary pros and cons of individual methods. Highly continuous genomes are required to evaluate the genomic architecture of the NKC; these were available for seven out of 89 species present in NCBI databases, representing seven out of 14 carnivore families. Due to the high evolutionary conservation of CLEC genes (**Figure 2**) and their good annotation in reference genomes, we focused on the characterization of the KLR sub-region of the NKC in carnivores.

Not all lineages of KLR genes identified in mammals (27) were found within the natural killer complex of Carnivora; namely, *KLRE* and *KLRI* are missing. On the other hand, the *KLRL* lineage was identified as a novel KLR gene subfamily specific to carnivores. While *KLRL* is a single gene in all Felidae, this locus is amplified in Viverridae. In Caniformia, there is no functional *KLRL* gene in the Canidae or Otariidae, and the Phocidae probably do not possess a functional gene despite the amplification of this locus. Amplification of *KLRL* has led to one functional gene accompanied by a various number of pseudogenes in the Mustelidae and Ursidae.

There are four lineages of KLR genes represented by a single gene. *KLRD* is conserved and present as a functional gene in all Carnivora families studied. *KLRG* was also found as a single gene in Carnivora. Although there are *KLRG2* genes annotated in the genomes of carnivores (cat, dog, ermine, bear, Californian sea lion and northern elephant seal) based on the mouse and human *KLRG2* genes. However, as KLRG2 in humans (and mice) differs from the structure of KLRG1 and their C-type lectin-like domains have only 33% homology, we suppose this is an erroneous designation for a different gene. *KLRJ* is highly conserved. It is present in all carnivores but has no evident signaling motifs, and its function remains unknown. Strikingly, a related *KLRJ-like* gene with an inhibitory motif was identified in the Mustelidae. The *KLRK* gene is also highly conserved and present in all carnivores. It is presumably an activating receptor in most carnivore families, while it shows features of a dual-purpose receptor in the Felidae.

Two lineages underwent duplication of the gene in only some carnivores. *KLRA* is present as a single gene in most carnivores studied except the Ursidae, in which this locus amplified. This is in agreement with the previous findings of an analysis of three seal and one sea lion species focused on specific loci (*KLRA* and *KIR3DL*) (37). The *KLRB* gene encodes an inhibitory receptor in all Carnivora; only Mustelidae have an amplified *KLRB* locus comparably to mice.

Three other lineages are characterized by the amplification of KLR genes. *KLRC1* appears to have experienced the most diversified expansion and pseudogenization, and is amplified in all studied families of carnivores. *KLRC2* is pseudogene in the Otariidae and Phocidae, while it is missing in the Ursidae. In other families, it underwent amplification and pseudogenization. A *KLRC3* gene encoding a dual-purpose receptor is present in the Felidae, Viverridae and Mustelidae, while it is missing in the Otariidae and Phocidae. On the other hand, it underwent amplification in the Canidae. Phylogenetic comparisons (**Figures 3**, **5**) showed that in the Ursidae this gene lost its arginine from the transmembrane domain, while in the Mustelidae another KLRC gene acquired one. *KLRF* amplified in a common ancestor of Carnivora with the genes for their presumed ligands (*CLEC2*) in their vicinity. Three *KLRF* loci followed different evolutionary paths. All three remain functional in the Felidae and Mustelidae, while two loci function in the Otariidae, Phocidae (with exceptions) and in some foxes. Only one locus works in the Viverridae, Canidae and Ursidae. As for *KLRH*, each Carnivora family contains species with at least duplicated or even expanded genes.

Expansions of KLR subfamilies (*KLRC* and *KLRH*) in carnivores seem to be family-specific, although some of their genes may have been duplicated in common ancestors. Receptors with presumed inhibitory signaling predominate in both the KLRC and KLRH subfamilies, partly due to preferential pseudogenization of activating receptors. A striking feature of the KLR genes of carnivores is the existence of some putative dual-purpose receptors containing both activating and inhibitory signaling motifs in the KLRC and KLRH subfamilies. Nothing is known about the signaling mechanisms of such receptors or their ligands, and functional studies are needed to address this issue. Some of these genes may represent an intermediate state in the evolution as they appear independently in only few species, and their expression could be silenced or cells bearing such a receptor could be eliminated during NK cell development. A distinct functional role may be supposed for KLRC3 as these receptors are likely to have been present in ancestors of Feliformia and Caniformia and the recent families of Felidae, Viverridae and Canidae have retained them. On the other hand, the role might be only a subtler regulation of cell activation as Ursidae have lost KLRC3 receptor and Mustelidae have acquired another one.

In general, the mammalian NKC is very variable in terms of its KLR gene content. At one extreme, the naked mole-rat (missing NK cells) has a very simplified complement of 6 genes (*KLRA*, *KLRC*, *KLRD*, *KLRE*, *KLRG* and *KLRK*) and 4 pseudogenes (two *KLRA, KLRC*, and *KLRI*). In contrast, the platypus possesses one *KLRB*, *KLRH*, and *KLRJ* gene, two *KLRA* and *KLRI* genes, expanded *KLRC*, *KLRF* and *KLRK* families (dozens of genes), and an extreme expansion of the *KLRD* family (54 genes) (23).

Carnivora represent an intermediate point in this diversity. Many aspects of Carnivora NKCs are typical for mammals. For example, although an expanded *KLRA* family has been identified in Rodentia, Lagomorpha and odd-toed ungulates, Carnivora are similar to the majority of mammals with only one *KLRA* gene, with the exception of Ursidae possessing two to three *KLRA* genes. The *KLRB*, *KLRD*, *KLRG* and *KLRK* genes are quite stable in their numbers. Again, in this aspect, Carnivora do not differ from the majority of other mammals. Moreover, the *KLRE* and *KLRI* lineages have been identified only in Rodentia and ungulates. This is in alignment with our finding that Carnivora do not possess *KLRE* and *KLRI* sequences. Amplifications of *KLRC* genes, such as those seen in carnivores, are present in the majority of mammalian species.

On the other hand, some carnivore KLR families are less typical. *KLRF* is duplicated in primates and further amplified in Carnivora, ungulates and Chiroptera. *KLRJ*, identified in all Carnivora families included in the present study, is not commonly found in mammals other than ungulates or Chiroptera. Further, *KLRH* appears to be specific to Carnivora, ungulates, Rodentia and Lagomorpha.

In comparison with other previously studied mammalian NKCs, ruminants are the most comparable to Carnivora. NKCs with expanded *KLRC* and *KLRH* genes and with duplicated *KLRD* have been identified in ruminants. Bovine and caprine *KLRC* genes located between the *KLRI* and *KLRA* loci are interspersed with specific *KLRH*-like genes (15). These are combinations of *KLRH* CTLD with *KLRC2* cytoplasmic, transmembrane and stalk regions, unlike Carnivora *KLRH* which is fully homologous to mouse *Klrh1*.

To mine as much information on KLR genes as possible, we have not limited our analyses solely to high-quality genomes. In so doing, some pitfalls must be considered. Short-read sequencing technologies limit the reassembly of repetitive and complex immune sequences. This leaves a number of genes fragmented on short scaffolds and can potentially produce false pseudogenization. On the other hand, long-read technologies help to span complex and repetitive sequences, resulting in long contigs and scaffolds that contain multiple immune genes with complete coding sequences (29), but their raw reads are error-prone. Comparisons of the NKC in draft-quality genomes of closely related species could be beneficial, but caution should be taken when assessing variability within species.

This point is well illustrated by the example of the giant panda. The NCBI reference genome ´Jingjing´ (Illumina, 10X Genomics, Flow-sorted chromosome sequencing) and another giant panda genome ´CPB_GP_2021´ (PacBio) contain seemingly complete NKCs and suggest intraspecific variability in the number of *KLRA* genes (**Supplementary Figure 6**). An unusual single *KLRA* gene with both inhibitory and activating signaling motifs in the ´Jingjing´ genome is different from the three *KLRA* genes in the ´CPB_GP_2021´ genome, of which one gene has activating and mutated inhibitory motifs and two are inhibitory genes. A VISTA alignment of these genomic regions (**Supplementary Figure 9**) revealed the combined nature of the *KLRA* gene (parts of *KLRA1* and *KLRA3* of ´CPB_GP_2021´) in ´Jingjing´. A BLAST search of the ´Jingjing´ genome identified another eight *KLRA* fragments scattered on various unplaced contigs. Their VISTA alignment against ´CPB_GP_2021´ (**Supplementary Figure 10**) suggests that the reference genome could in fact also have three *KLRA* genes. Further, the *KLRL2* gene contains a stop codon in ´CPB_GP_2021´, while an intact gene was found in the ´Jingjing´ genome. It is plausible that at least some of this intraspecific variability is in fact an artifact of the different sequencing and assembly technologies employed and errors in the resultant genomes.

A comparison of different assemblies of the domestic cat genome is another example which seemingly suggests the existence of within-species variability. In this case, it is the number of *KLRC* and *KLRH* genes which appear variable (**Supplementary Figure 1**). As these assemblies were obtained by different methods, this presumed variability needs to be confirmed.

For comparison, two dingo genomes constructed using a combination of long- and short-read sequencing as hybrid assemblies also differ in the number of *KLRC* genes. However, in this case, one haplotype is in common with domestic dog genomes while the second one is found in the grey wolf, which supports the idea of such variability in the dingo (**Supplementary Figure 2**). In this context, we assume that even KLR sequences assembled based on short reads could be helpful for identifying their presence/absence in their respective species and for phylogenetic analyses based on interspecific differences. Nevertheless, we cannot exclude the possibility of technical artifacts (such as the number of functional genes vs. pseudogenes) produced by our analyses of genomes obtained by different methods, and we thus have to consider the comparative view of the carnivore NKC as a still-emerging picture of such a complex genomic region.

A good knowledge of the structure of the complex immune response related genomic regions may contribute to a better understanding of NK cell development and function with implications for veterinary medicine (38). Single-cell RNA sequencing of peripheral blood mononuclear cells has been used to identify NK cell populations as documented for the domestic cat, tiger and dog (39). Recently, a targeted analysis of KLRA showed its almost exclusive expression in canine NK cells (40). A high-quality annotation of the reference transcriptome is a prerequisite for single-cell RNA sequencing. The data obtained in this study cover an important part of the carnivore families and may help to characterize the NKC genomic region of other wild felid species. This knowledge may then be used for conservation efforts in endangered species (41).

## Data Availability

The original data are freely available in the public database NCBI Genome (RRID:SCR_002474) and are referenced in **Supplementary Table 1**. The contributions presented in the study are included in the article and **Supplementary Material**. Further inquiries can be directed to the corresponding author.

## References

[1] Allan AJ Sanderson ND Gubbins S Ellis SA Hammond JA. Cattle NK cell heterogeneity and the influence of MHC class I. J Immunol. (2015) 195:2199–206. doi: 10.4049/jimmunol.1500227 PMC4543905 26216890

[2] Vivier E Raulet DH Moretta A Caligiuri MA Zitvogel L Lanier LL. Innate or adaptive immunity? The example of natural killer cells. Science. (2011) 331:44–9. doi: 10.1126/science.1198687 PMC3089969 21212348

[3] Hamerman JA Ogasawara K Lanier LL. NK cells in innate immunity. Curr Opin Immunol. (2005) 17:29–35. doi: 10.1016/j.col.2004.11.001 15653307

[4] Sojka DK Plougastel-Douglas B Yang L Pak-Wittel MA Artyomov MN Ivanova Y. Tissue-resident natural killer (NK) cells are cell lineages distinct from thymic and conventional splenic NK cells. Elife. (2014) 3:e01659. doi: 10.7554/eLife.01659 24714492 PMC3975579

[5] Sojka DK Yang L Yokoyama WM. Uterine natural killer cells. Front Immunol. (2019) 10:960. doi: 10.3389/fimmu.2019.00960 31118936 PMC6504766

[6] Huhn O Zhao X Esposito L Moffett A Colucci F Sharkey AM. How do uterine natural killer and innate lymphoid cells contribute to successful pregnancy? Front Immunol. (2021) 12:607669. doi: 10.3389/fimmu.2021.607669 34234770 PMC8256162

[7] Lanier LL. NK cell receptors. Annu Rev Immunol. (1998) 16:359–93. doi: 10.1146/annurev.immunol.16.1.359 9597134

[8] Hiby SE Apps R Sharkey AM Farrell LE Gardner L Mulder A. Maternal activating KIRs protect against human reproductive failure mediated by fetal HLA-C2. J Clin Invest. (2010) 120:4102–10. doi: 10.1172/JCI43998 PMC2964995 20972337

[9] Lima PDA Tu MM Rahim MMA Peng AR Croy PA Makrigiannis AP. Ly49 receptors activate angiogenic mouse DBA^+^ uterine natural killer cells. Cell Mol Immunol. (2014) 11:467–76. doi: 10.1038/cmi.2014.44 PMC4197209 24954223

[10] Kelley J Walter L Trowsdale J. Comparative genomics of natural killer cell receptor gene clusters. PloS Genet. (2005) 1:129–39. doi: 10.1371/journal.pgen.0010027 PMC1193534 16132082

[11] Ito M Maruyama T Saito N Koganei S Yamamoto K Matsumoto N. Killer cell lectin-like receptor G1 binds three members of the classical cadherin family to inhibit NK cell cytotoxicity. J Exp Med. (2006) 203:289–95. doi: 10.1084/jem20051986 PMC2118217 16461340

[12] Zhang Q Rahim MMA Allan DSJ Tu MM Belanger S Abou-Samra E. Mouse Nkrp1-Clr gene cluster sequence and expression analyses reveal conservation of tissue-specific MHC-independent immunosurveillance. PloS One. (2012) 7:e50561. doi: 10.1371/journal.pone.0050561 23226525 PMC3514311

[13] Scur M Parsons BD Dey S Makrigiannis AP. The diverse roles of C-type lectin-like receptors in immunity. Front Immunol. (2023) 14:1126043. doi: 10.3389/fimmu.2023.1126043 36923398 PMC10008955

[14] Saether PC Westgaard IH Hoelsbrekken SE Benjamin J Lanier LL Fossum S. KLRE/I1 and KLRE/I2: a novel pair of heterodimeric receptors that inversely regulate NK cell cytotoxicity. J Immunol. (2008) 181:3177–82. doi: 10.4049/jimmunol.181.5.3177 PMC2577148 18713988

[15] Schwartz JC Gibson MS Heimeier D Koren S Phillippy AM Bickhart DM. The evolution of the natural killer complex; a comparison between mammals using new high-quality genome assemblies and targeted annotation. Immunogenetics. (2017) 69:255–69. doi: 10.1007/s00251-017-0973-y PMC5350243 28180967

[16] Futas J Oppelt J Jelinek A Elbers JP Wijacki J Knoll A. Natural killer cell receptor genes in camels: Another Mammalian Model. Front Genet. (2019) 10:620. doi: 10.3389/fgene.2019.00620 31312212 PMC6614441

[17] Vance RE Kraft JR Altman JD Jensen PE Raulet DH. Mouse CD94/NKG2A is a natural killer cell receptor for the nonclassical major histocompatibility complex (MHC) class I molecule Qa-1(b). J Exp Med. (1998) 188:1841–8. doi: 10.1084/jem.188.10.1841 PMC2212405 9815261

[18] Braud VM Allan DS O’Callaghan CA Söderström K D’Andrea A Ogg GS. HLA-E binds to natural killer cell receptors CD94/NKG2A, B and C. Nature. (1998) 391:795–9. doi: 10.1038/35869 9486650

[19] Le Drean E Vely F Olcese L Cambiaggi A Guia S Krystal G. Inhibition of antigen-induced T cell response and antibody-induced NK cell cytotoxicity by NKG2A: association of NKG2A with SHP-1 and SHP-2 protein-tyrosine phosphatase. Eur J Immunol. (1998) 28:264–76. doi: 10.1002/(SICI)1521-4141(199801)28:01<264::AID-IMMU264>3.0.CO;2-O 9485206

[20] Lanier LL Corliss B Wu J Phillips JH. Association of DAP12 with activating CD94/NKG2C NK cell receptors. Immunity. (1998) 8:693–701. doi: 10.1016/S1074-7613(00)80574-9 9655483

[21] Lanier LL. NKG2D receptor and its ligands in host defense. Cancer Immunol Res. (2015) 3:575–82. doi: 10.1158/2326-6066.CIR-15-0098 PMC4457299 26041808

[22] Daws MR Ke-Zheng D Zinöcker S Naper Ch Kveberg L Hedrich HJ. Identification of an MHC class I ligand for the single member of a killer cell lectin-like receptor family, KLRH1. J Immunol. (2012) 189:5178–84. doi: 10.4049/jimmunol.1201983 23100519

[23] Hilton HG Rubinstein ND Janki P Ireland AT Bernstein N Fong NL. Single-cell transcriptomics of the naked mole-rat reveals unexpected features of mammalian immunity. PloS Biol. (2019) 17:e3000528. doi: 10.1371/journal.pbio.3000528 31751331 PMC6894886

[24] Dimasi N Biassoni R. Structural and functional aspects of the Ly49 natural killer cell receptors. Immunol Cell Biol. (2005) 83:1–8. doi: 10.1111/j.1440-1711.2005.01301.x 15661035

[25] Takahashi T Yawata M Raudsepp T Lear TL Chowdhary BP Antczak DF. Natural killer cell receptors in the horse: evidence for the existence of multiple transcribed LY49 genes. Eur J Immunol. (2004) 34:773–84. doi: 10.1002/eji.200324695 14991607

[26] Futas J Horin P. Natural killer cell receptor genes in the family *Equidae*: not only Ly49. PloS One. (2013) 8:e64736. doi: 10.1371/journal.pone.0064736 23724088 PMC3665701

[27] Hao L Klein J Nei M. Heterogeneous but conserved natural killer receptor gene complexes in four major orders of mammals. Proc Natl Acad Sci U S A. (2006) 103:3192–7. doi: 10.1073/pnas.0511280103 PMC1413923 16492762

[28] Storset AK Slettedal I Williams JL Law A Dissen E. Natural killer cell receptors in cattle: a bovine killer cell immunoglobulin-like receptor multigene family contains members with divergent signaling motifs. Eur J Immunol. (2003) 33:980–90. doi: 10.1002/eji.200323710 12672064

[29] Peel E Silver L Brandies P Zhu Y Cheng Y Hogg CJ. Best genome sequencing strategies for annotation of complex immune gene families in wildlife. GigaScience. (2022) 11:giac100. doi: 10.1093/gigascience/giac100 36310247 PMC9618407

[30] Jelinek AL Futas J Burger PA Horin P. Comparative genomics of the Leukocyte Receptor Complex in carnivores. Front Immunol. (2023) 14:1197687. doi: 10.3389/fimmu.2023.1197687 37234165 PMC10206138

[31] Han Y Zhang M Li N Chen T Zhang Y Wan T. KLRL1, a novel killer cell lectinlike receptor, inhibits natural killer cell cytotoxicity. Blood. (2004) 104:2858–66. doi: 10.1182/blood-2004-03-0878 15238421

[32] Liu G Yin S Li P Han Y Zheng Y Zhang Y. mKLRL1 regulates the maturation of dendritic cells and plays important roles in immune tolerance. Am J Transl Res. (2019) 11:300–13. PMC6357304 30787988

[33] Kapustin Y Souvorov A Tatusova T Lipman D. Splign: algorithms for computing spliced alignments with identification of paralogs. Biol Direct. (2008) 3:20. doi: 10.1186/1745-6150-3-20 18495041 PMC2440734

[34] Hall TA. BioEdit: a user-friendly biological sequence alignment editor and analysis program for windows 95/98/NT. Nucleic Acids Symp Ser. (1999) 41:95–8.

[35] Kumar S Stecher G Li M Knyaz C Tamura K. MEGA X: molecular evolutionary genetics analysis across computing platforms. Mol Biol Evol. (2018) 35:1547. doi: 10.1093/molbev/msy096 29722887 PMC5967553

[36] Bauer B Steinle A. HemITAM: A single tyrosine motif that packs a punch. Sci Signal. (2017) 10:eaan3676. doi: 10.1126/scisignal.aan3676 29208681

[37] Hammond JA Guethlein LA Abi-Rached L Moesta AK Parham P. Evolution and survival of marine carnivores did not require a diversity of killer cell Ig-like receptors or Ly49 NK cell receptors. J Immunol. (2009) 182:3618–27. doi: 10.4049/jimmunol.0803026 PMC2754575 19265140

[38] Gingrich AA Reiter TE Judge SJ York D Yanagisawa M Razmara A. Comparative immunogenomics of canine natural killer cells as immunotherapy target. Front Immunol. (2021) 12:670309. doi: 10.3389/fimmu.2021.670309 34594320 PMC8476892

[39] Li Z Sun C Wang F Wang X Zhu J Luo L. Molecular mechanisms governing circulating immune cell heterogeneity across different species revealed by single-cell sequencing. Clin Transl Med. (2022) 12:e689. doi: 10.1002/ctm2.689 35092700 PMC8800483

[40] Gingrich AA Razmara AM Gingrich PW Rebhun RB Murphy WJ Kent MS. Missing a “missing self” mechanism: modeling and detection of Ly49 expression in canine NK cells. Immunohorizons. (2023) 7:760–70. doi: 10.4049/immunohorizons.2300092 PMC10696421 37971282

[41] Meißner R Mokgokong P Pretorius C Winter S Labuschagne K Kotze A. Diversity of selected toll-like receptor genes in cheetahs (*Acinonyx jubatus*) and African leopards (*Panthera pardus pardus*). Sci Rep. (2024) 14:3756. doi: 10.1038/s41598-024-54076-y 38355905 PMC10866938

