# Corrigendum: Comparative genomics of the Natural Killer Complex in carnivores

**DOI:** 10.3389/fimmu.2024.1521966

**Published:** 2024-11-28

**Authors:** Jan Futas, April L. Jelinek, Pamela A. Burger, Petr Horin

**Affiliations:** ^1^ Research Group Animal Immunogenomics, Central European Institute of Technology (CEITEC) VETUNI, Brno, Czechia; ^2^ Department of Animal Genetics, Faculty of Veterinary Medicine, University of Veterinary Sciences Brno (VETUNI), Brno, Czechia; ^3^ Research Institute of Wildlife Ecology, University of Veterinary Medicine Vienna (VETMEDUNI), Vienna, Austria

**Keywords:** *CLEC*, *KLR*, Natural Killer Complex, carnivore, Felids, genomes

In the published article, there was an error in the author list, and author “Petr Horin” was erroneously named as corresponding author. The corrected corresponding author appears below:

“Pamela A. Burger

Pamela.burger@vetmeduni.ac.at”

The authors apologize for this error and state that this does not change the scientific conclusions of the article in any way. The original article has been updated.

