# Supplementary material for: Comparative genomics of the Natural Killer Complex in carnivores
Source: Front Immunol. 2024 Oct 3;15:1459122. doi: 10.3389/fimmu.2024.1459122 (PMC11484026; doi:10.3389/fimmu.2024.1459122)
Supplement: Supplementary file 3 [file DataSheet3.pdf]

Supplementary Material

Comparative genomics of the Natural Killer Complex in carnivores

Jan Futas, April L. Jelinek, Pamela A. Burger, Petr Horin*

*** Correspondence:** Petr Horin: horin@ics.muni.cz

1 Supplementary Data

Supplementary Data 1 – Coding sequences of CLEC and KLR genes of the mouse (C57BL/6J strain), domestic cat, masked palm civet, gray wolf, ermine, California sea lion, northern elephant seal, and brown bear. Sequences were isolated from NCBI reference genomes according to the mRNA accession numbers listed in Supplementary Table 2 or by cross-species comparison. (FASTA)

Supplementary Data 2 – Coding sequences of all KLR genes identified in carnivores. Sequences were extracted from the NCBI genomes listed in Supplementary Table 1. KLR mRNA models of representative species of Carnivora families were used for Splign alignments of BLAST-retrieved contigs in each species of the family in question. (FASTA)

2 Supplementary Figures and Tables

2.1 Supplementary Tables

Supplementary Table 1 – List of analyzed NCBI genomes of carnivores. (Excel)

Supplementary Table 2 – Annotations of the Natural Killer Complex in seven carnivores: domestic cat, masked palm civet, gray wolf, ermine, California sea lion, northern elephant seal, and brown bear. (Excel)

Supplementary Table 3 – Numbers of KLR genes identified in individual species of carnivores. (PDF)

2.2 Supplementary Figures

Supplementary Figures 1-6 – Comparison of the KLR sub-region in families of carnivores. Functional genes *(solid color arrows)*, potentially functional genes with 1-2 bp indels or mutations in splice sites (*striped arrows*) and pseudogenes/fragments (*open arrows*) are drawn in the direction of transcription, not to scale. Marks denote the type of signaling motif(s) of the corresponding receptor: activating (+), inhibitory (-), dual purpose (+/-) and switch motif (x). Mutated motifs are given in parentheses.

Supplementary Figure 1. Structure of the Natural Killer Complex in Felidae. Assemblies of the following species are compared: domestic cat, jungle cat, black-footed cat, Pallas's cat, leopard cat, fishing cat, cheetah, Geoffroy's cat, Canada lynx, Spanish lynx, bobcat, Sunda clouded leopard, Clouded leopard, lion, jaguar, leopard, tiger, snow leopard, puma and jaguarundi. (PDF)

Supplementary Figure 2. Structure of the Natural Killer Complex in Canidae. Assemblies of the following species are compared: coyote, gray wolf, dingo, dog, maned wolf, African hunting dog, raccoon dog, bat-eared fox, gray fox, Corsac fox, Tibetan sand fox, Arctic fox and red fox. (PDF)

Supplementary Figure 3. Structure of the Natural Killer Complex in Mustelidae. Assemblies of the following species are compared: tayra, northern sea otter, American wolverine, North American river otter, Eurasian river otter, yellow-throated marten, European pine marten, Eurasian badger, ermine, steppe polecat, European mink, black-footed ferret, domestic ferret and American mink. (PDF)

Supplementary Figure 4. Structure of the Natural Killer Complex in Otariidae. Assemblies of the following species are compared: Guadalupe fur seal, northern fur seal, Steller sea lion and California sea lion. (PDF)

Supplementary Figure 5. Structure of the Natural Killer Complex in Phocidae. Assemblies of the following species are compared: gray seal, Weddell seal, northern elephant seal, southern elephant seal, Hawaiian monk seal, harbor seal and Baikal seal. (PDF)

Supplementary Figure 6. Structure of the Natural Killer Complex in Ursidae. Assemblies of the following species are compared: giant panda, Malayan sun bear, spectacled bear, American black bear, brown bear and polar bear. (PDF)

Supplementary Figure 7. Phylogeny reconstruction of KLRC and KLRD proteins in carnivores. The bootstrap consensus tree using the Neighbor-Joining method based on p-distances (MEGA X) is depicted with branch node values (greater than 50%) as the percentage of trees (out of 1000 replicates) in which the associated sequences clustered together. The analysis involved 415 amino acid sequences. The tree was rooted to KLRD sequences. Carnivore families are highlighted: Felidae (*yellow*), Canidae (*green*), Mustelidae (*magenta*), Otariidae (*cyan*), Phocidae (*blue*) and Ursidae (*brown*). (PDF)

Supplementary Figure 8. Phylogeny of Carnivora KLRH proteins compared to KLRA and KLRL proteins. The bootstrap consensus tree using the Neighbor-Joining method based on p-distances (MEGA X) is depicted with branch node values (greater than 50%) as the percentage of trees (out of 1000 replicates) in which the associated sequences clustered together. The analysis involved 255 amino acid sequences. The tree was rooted to KLRA sequences. Carnivore families are highlighted: Felidae (*yellow*), Canidae (*green*), Mustelidae (*magenta*), Otariidae (*cyan*), Phocidae (*blue*) and Ursidae (*brown*). (PDF)

Supplementary Figure 9. VISTA comparison of *KLRA* genes in two giant panda genomes. A plot of the LAGAN alignments of the *Ailuropoda melanoleuca* reference genome "Jingjing" (ASM200744v3) to the genome sequence "CPB_GP_2021" (CPB_AME_v1) is depicted. Conserved regions with more than 70% sequence similarity over a 100 base pair window are colored: non-coding sequences (*apricot*), exons (*purple*) and untranslated regions (*cyan*). The region from *MAGOHB* to the *KLRH* gene was analyzed for the presence of long interspersed repeats (*red*), short interspersed repeats (*green*), long terminal repeats (*pink*), and different repeats (*yellow*) known in Carnivora genomes, and genes (*dark gray arrows*) are annotated in the upper line. While the *MAGOHB* and *KLRH* genes are concordant between genomes, the *KLRA* gene in the reference genome is probably a combination of the *KLRA3* and *KLRA1* sequences found in the other panda´s genome. (PDF)

Supplementary Figure 10. VISTA alignment of fragments of *KLRA* genes from the reference genome of the giant panda. A plot of the Shuffle-LAGAN alignments of eight contigs bearing parts of *KLRA* found by BLAST in the "Jingjing" genome to the genome sequence "CPB_GP_2021" is depicted. The color scheme applies as for Supplementary Figure 7 (see above). Contig (*light gray arrows*) rearrangements suggest that the reference genome may contain disassembled *KLRA3* and *KLRA2* genes. (PDF)
