# Supplementary material for: Comparative genomics of the Natural Killer Complex in carnivores
Source: Front Immunol. 2024 Oct 3;15:1459122. doi: 10.3389/fimmu.2024.1459122 (PMC11484026; doi:10.3389/fimmu.2024.1459122)
Supplement: Supplementary file 4 [file Image1.pdf]

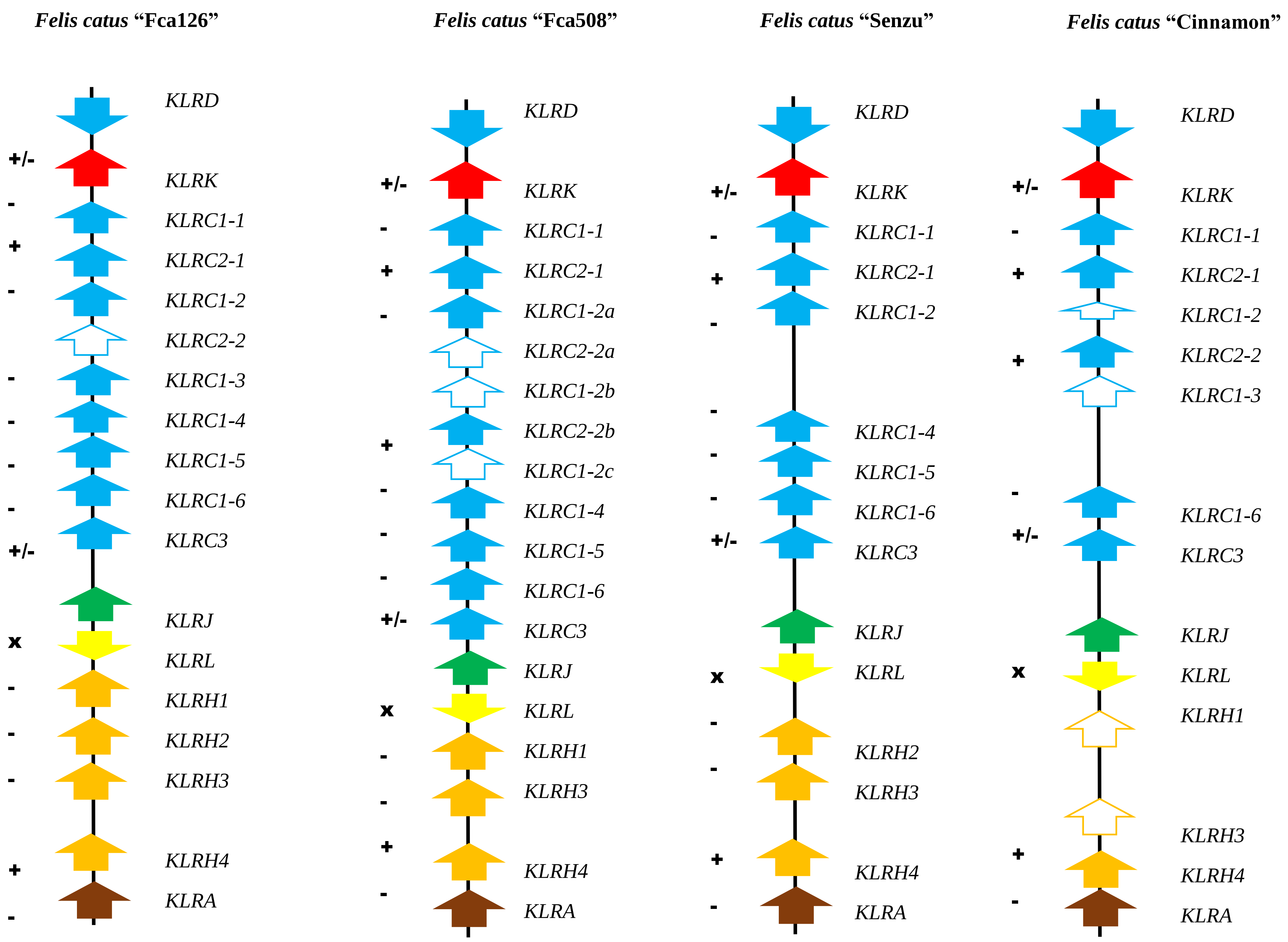
Felis catus "Fca126"
KLRD
KLRK
KLRC1-1
KLRC2-1
KLRC1-2
KLRC2-2
KLRC1-3
KLRC1-4
KLRC1-5
KLRC1-6
KLRC3
KLRJ
KLRL
KLRH1
KLRH2
KLRH3
KLRH4
KLRA
Felis catus "Fca508"
KLRD
KLRK
KLRC1-1
KLRC2-1
KLRC1-2a
KLRC2-2a
KLRC1-2b
KLRC2-2b
KLRC1-2c
KLRC1-4
KLRC1-5
KLRC1-6
KLRC3
KLRJ
KLRL
KLRH1
KLRH3
KLRH4
KLRA
Felis catus "Senzu"
KLRD
KLRK
KLRC1-1
KLRC2-1
KLRC1-2
KLRC1-4
KLRC1-5
KLRC1-6
KLRC3
KLRJ
KLRL
KLRH2
KLRH3
KLRH4
KLRA
Felis catus "Cinnamon"
KLRD
KLRK
KLRC1-1
KLRC2-1
KLRC1-2
KLRC2-2
KLRC1-3
KLRC1-6
KLRC3
KLRJ
KLRL
KLRH1
KLRH3
KLRH4
KLRA

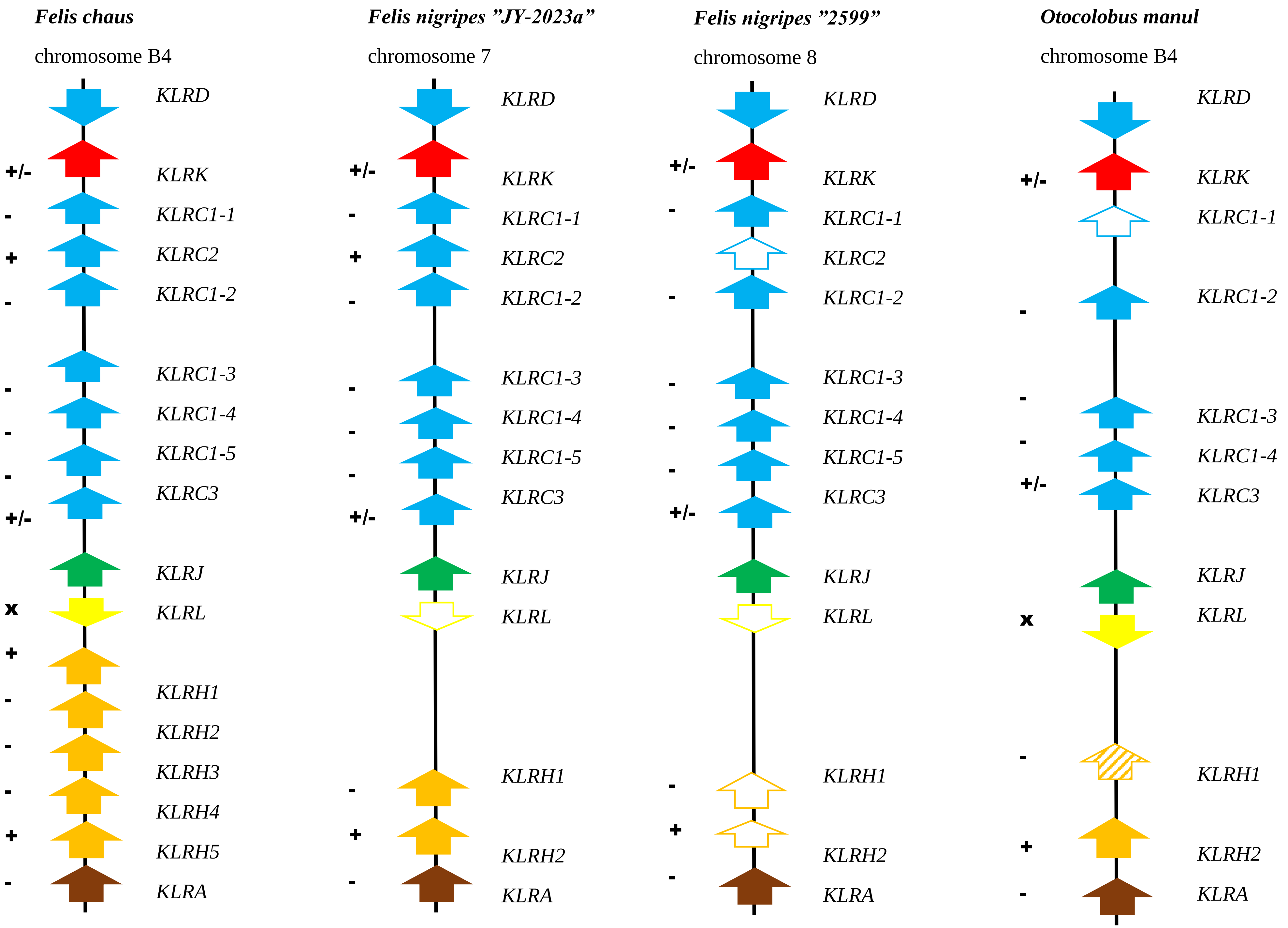
Felis chaus
chromosome B4
KLRD
+/-
KLRK
-
KLRC1-1
+
KLRC2
-
KLRC1-2
-
KLRC1-3
-
KLRC1-4
-
KLRC1-5
+/-
KLRC3
KLRJ
x
KLRL
+
-
KLRH1
-
KLRH2
-
KLRH3
+
KLRH4
-
KLRH5
KLRA
Felis nigripes "JY-2023a"
chromosome 7
KLRD
KLRK
KLRC1-1
KLRC2
KLRC1-2
KLRC1-3
KLRC1-4
KLRC1-5
KLRC3
KLRJ
KLRL
KLRH1
KLRH2
KLRA
Felis nigripes "2599"
chromosome 8
KLRD
KLRK
KLRC1-1
KLRC2
KLRC1-2
KLRC1-3
KLRC1-4
KLRC1-5
KLRC3
KLRJ
KLRL
KLRH1
KLRH2
KLRA
Otocolobus manul
chromosome B4
KLRD
KLRK
KLRC1-1
KLRC1-2
KLRC1-3
KLRC1-4
KLRC3
KLRJ
KLRL
KLRH1
KLRH2
KLRA

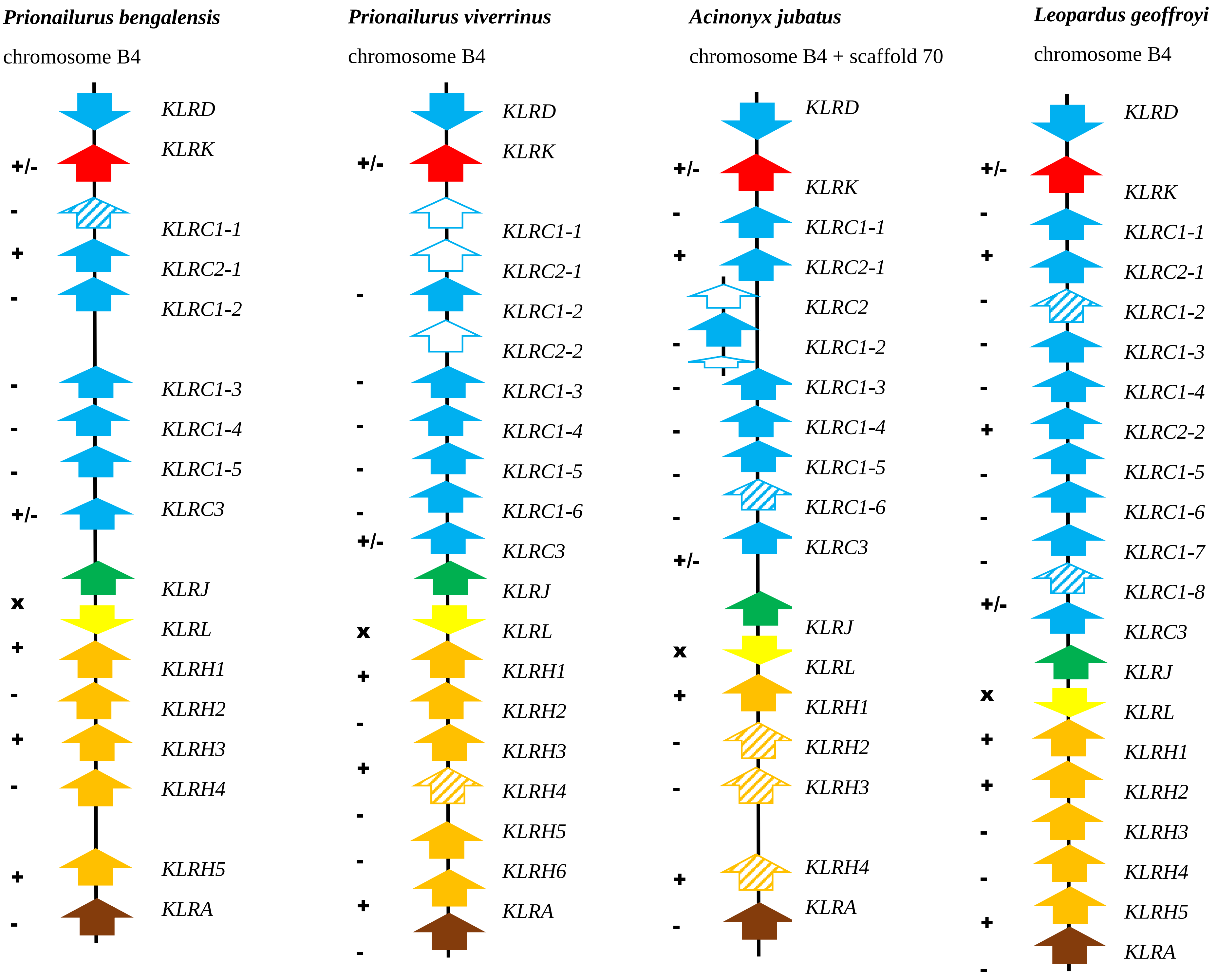

Prionailurus bengalensis
chromosome B4
KLRD
+/- KLRK
- KLRC1-1
+ KLRC2-1
- KLRC1-2
- KLRC1-3
- KLRC1-4
- KLRC1-5
+/- KLRC3
KLRJ
x KLRL
+ KLRH1
- KLRH2
+ KLRH3
- KLRH4
+ KLRH5
- KLRA
Prionailurus viverrinus
chromosome B4
KLRD
+/- KLRK
KLRC1-1
KLRC2-1
- KLRC1-2
KLRC2-2
- KLRC1-3
- KLRC1-4
- KLRC1-5
- KLRC1-6
+/- KLRC3
KLRJ
x KLRL
+ KLRH1
- KLRH2
+ KLRH3
- KLRH4
- KLRH5
+ KLRH6
- KLRA
Acinonyx jubatus
chromosome B4 + scaffold 70
KLRD
+/- KLRK
- KLRC1-1
+ KLRC2-1
KLRC2
- KLRC1-2
- KLRC1-3
- KLRC1-4
- KLRC1-5
- KLRC1-6
KLRC3
+/-
KLRJ
x KLRL
+ KLRH1
- KLRH2
- KLRH3
+ KLRH4
- KLRA
Leopardus geoffroyi
chromosome B4
KLRD
+/- KLRK
- KLRC1-1
+ KLRC2-1
- KLRC1-2
- KLRC1-3
- KLRC1-4
+ KLRC2-2
- KLRC1-5
- KLRC1-6
- KLRC1-7
KLRC1-8
+/- KLRC3
KLRJ
x KLRL
+ KLRH1
+ KLRH2
- KLRH3
- KLRH4
+ KLRH5
- KLRA

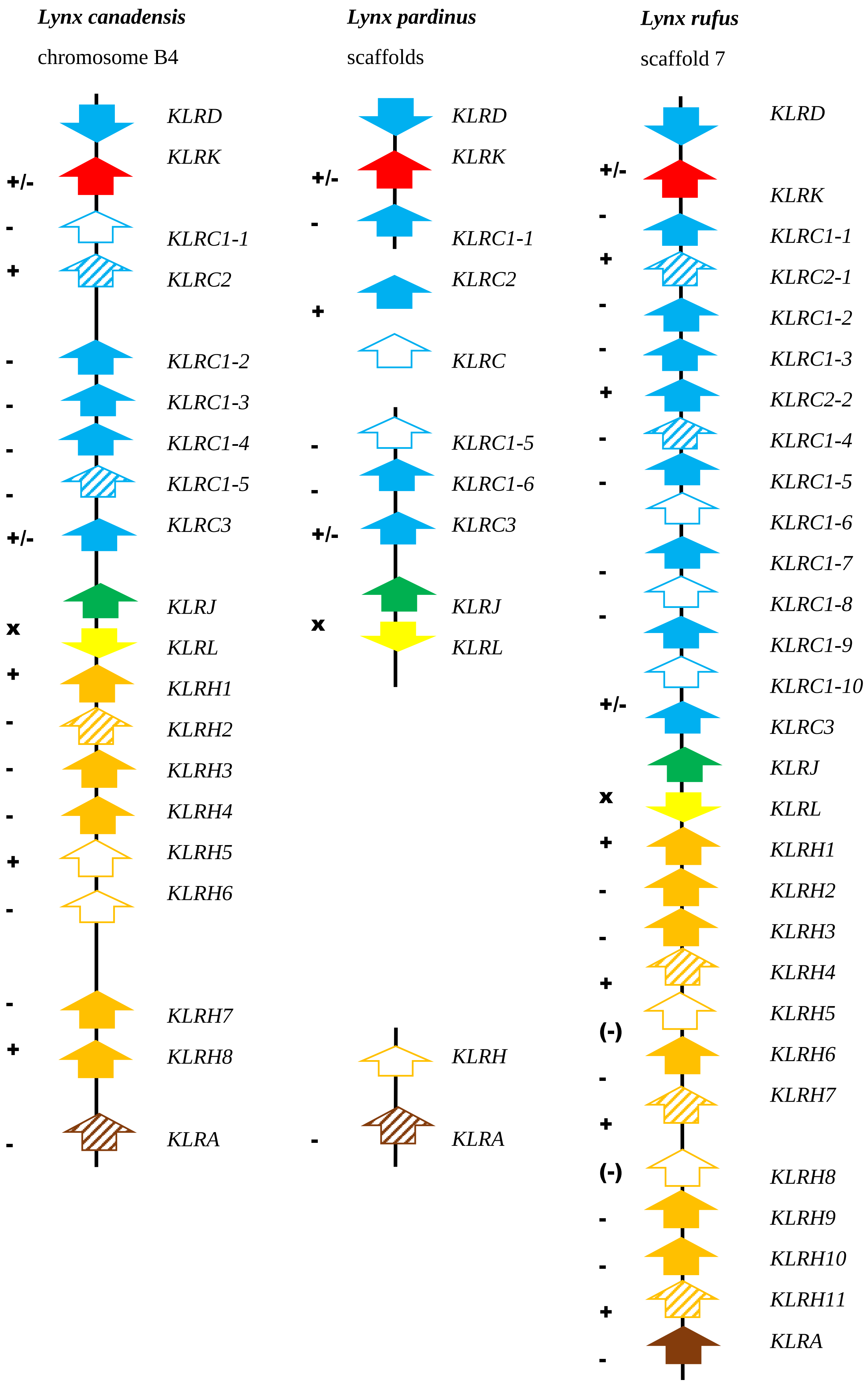
Lynx canadensis
chromosome B4
KLRD
KLRK
KLRC1-1
KLRC2
KLRC1-2
KLRC1-3
KLRC1-4
KLRC1-5
KLRC3
KLRJ
KLRL
KLRH1
KLRH2
KLRH3
KLRH4
KLRH5
KLRH6
KLRH7
KLRH8
KLRA
Lynx pardinus
scaffolds
KLRD
KLRK
KLRC1-1
KLRC2
KLRC
KLRC1-5
KLRC1-6
KLRC3
KLRJ
KLRL
KLRH
KLRA
Lynx rufus
scaffold 7
KLRD
KLRK
KLRC1-1
KLRC2-1
KLRC1-2
KLRC1-3
KLRC2-2
KLRC1-4
KLRC1-5
KLRC1-6
KLRC1-7
KLRC1-8
KLRC1-9
KLRC1-10
KLRC3
KLRJ
KLRL
KLRH1
KLRH2
KLRH3
KLRH4
KLRH5
KLRH6
KLRH7
KLRH8
KLRH9
KLRH10
KLRH11
KLRA

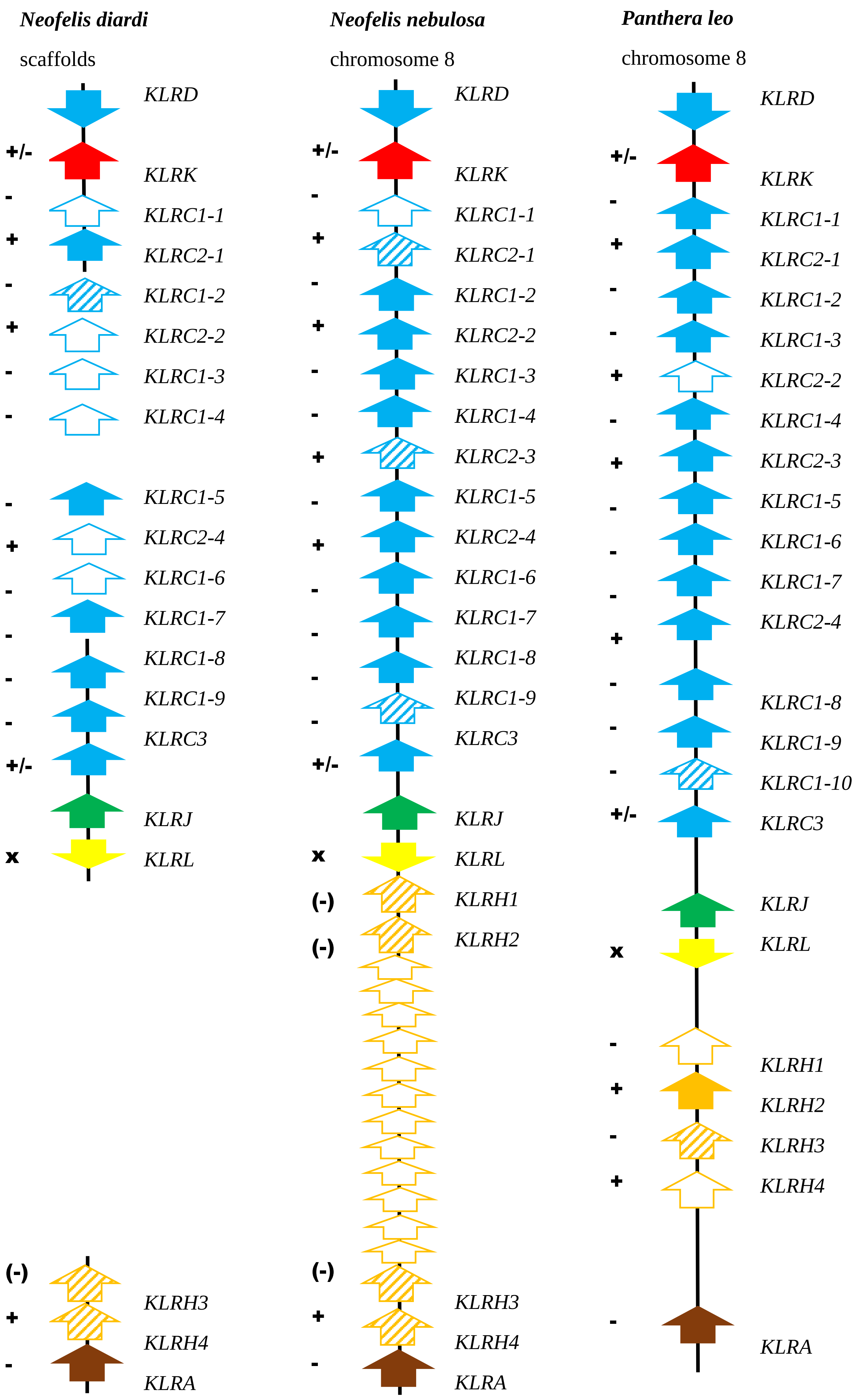

Neofelis diardi
scaffolds
KLRD
+/- KLRK
- KLRC1-1
+ KLRC2-1
- KLRC1-2
+ KLRC2-2
- KLRC1-3
- KLRC1-4
- KLRC1-5
+ KLRC2-4
- KLRC1-6
- KLRC1-7
- KLRC1-8
- KLRC1-9
+/- KLRC3
KLRJ
x KLRL
(-) KLRH3
+ KLRH4
- KLRA
Neofelis nebulosa
chromosome 8
KLRD
+/- KLRK
- KLRC1-1
+ KLRC2-1
- KLRC1-2
+ KLRC2-2
- KLRC1-3
- KLRC1-4
+ KLRC2-3
- KLRC1-5
+ KLRC2-4
- KLRC1-6
- KLRC1-7
- KLRC1-8
- KLRC1-9
+/- KLRC3
KLRJ
x KLRL
(-) KLRH1
(-) KLRH2
(-) KLRH3
+ KLRH4
- KLRA
Panthera leo
chromosome 8
KLRD
+/- KLRK
- KLRC1-1
+ KLRC2-1
- KLRC1-2
- KLRC1-3
+ KLRC2-2
- KLRC1-4
+ KLRC2-3
- KLRC1-5
- KLRC1-6
- KLRC1-7
+ KLRC2-4
- KLRC1-8
- KLRC1-9
- KLRC1-10
+/- KLRC3
KLRJ
x KLRL
- KLRH1
+ KLRH2
- KLRH3
+ KLRH4
- KLRA

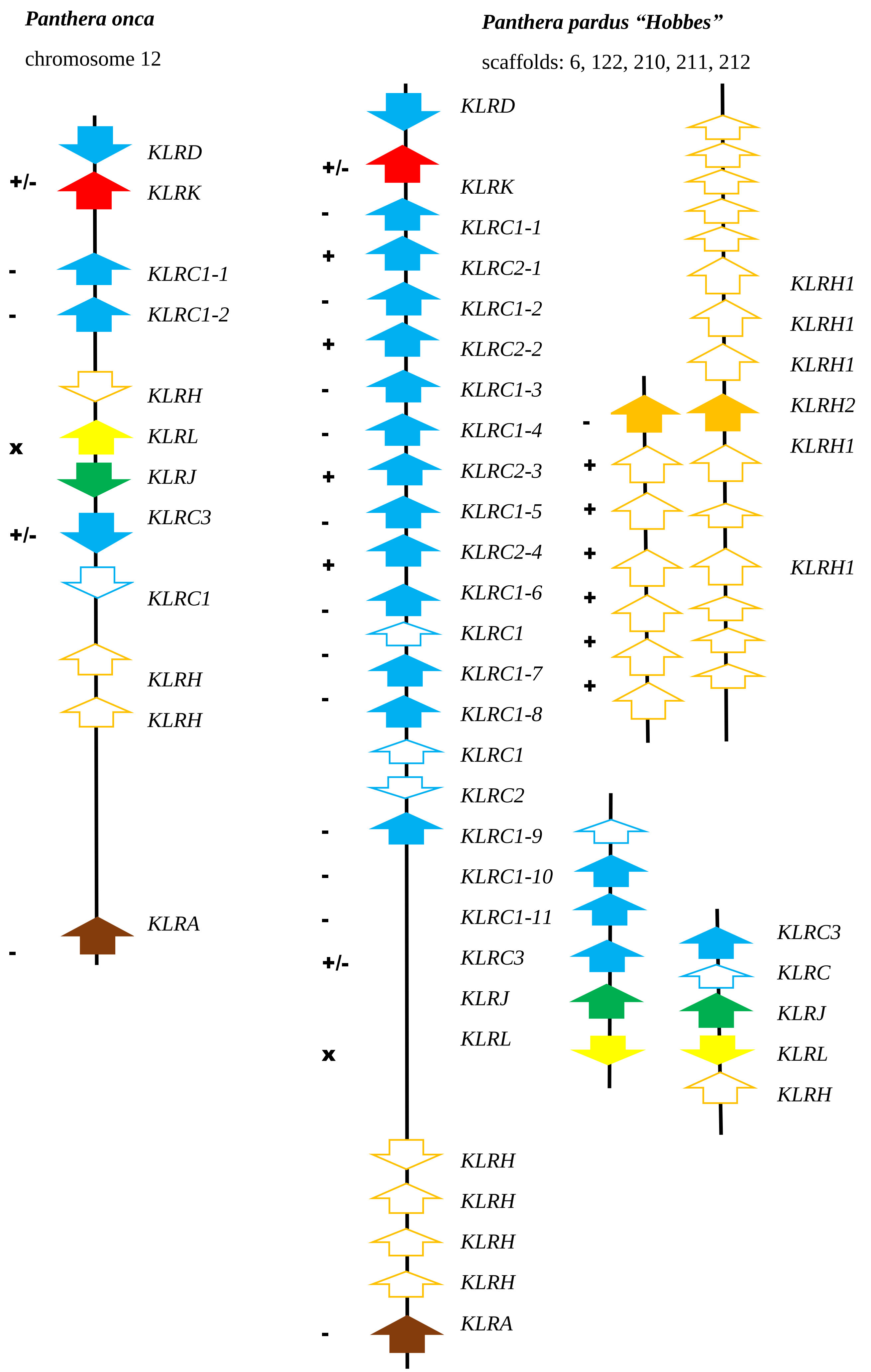

Panthera onca
chromosome 12
KLRD
+/-
KLRK
-
KLRC1-1
-
KLRC1-2
KLRH
x
KLRL
KLRJ
KLRC3
+/-
KLRC1
KLRH
KLRH
KLRA
-
Panthera pardus "Hobbes"
scaffolds: 6, 122, 210, 211, 212
KLRD
+/-
KLRK
-
KLRC1-1
+
KLRC2-1
-
KLRC1-2
+
KLRC2-2
-
KLRC1-3
-
KLRC1-4
+
KLRC2-3
-
KLRC1-5
+
KLRC2-4
-
KLRC1-6
KLRC1
-
KLRC1-7
-
KLRC1-8
KLRC1
KLRC2
-
KLRC1-9
-
KLRC1-10
-
KLRC1-11
+/-
KLRC3
KLRJ
x
KLRL
KLRH
KLRH
KLRH
KLRH
-
KLRA
KLRH1
KLRH1
KLRH1
KLRH2
KLRH1
KLRH1
-
+
+
+
+
+
+
KLRC3
KLRC
KLRJ
KLRL
KLRH

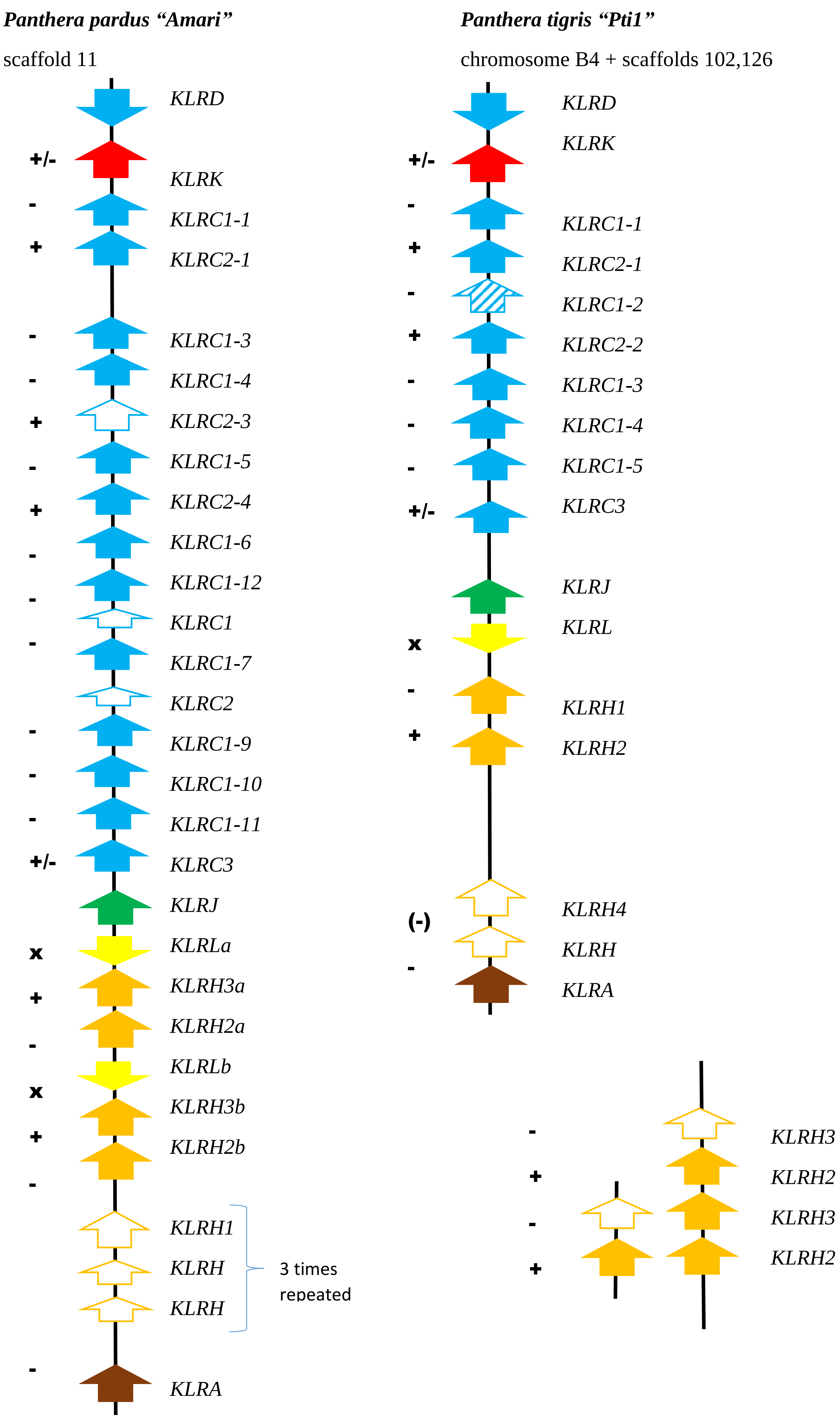

Panthera pardus “Amari”
scaffold 11
KLRD
+/-
KLRK
-
KLRC1-1
+
KLRC2-1
-
KLRC1-3
-
KLRC1-4
+
KLRC2-3
-
KLRC1-5
+
KLRC2-4
-
KLRC1-6
-
KLRC1-12
KLRC1
-
KLRC1-7
KLRC2
-
KLRC1-9
-
KLRC1-10
-
KLRC1-11
+/-
KLRC3
KLRJ
x
KLRLa
+
KLRH3a
-
KLRH2a
KLRLb
x
KLRH3b
+
KLRH2b
-
KLRH1
KLRH
KLRH
3 times repeated
-
KLRA
Panthera tigris “Pti1”
chromosome B4 + scaffolds 102,126
KLRD
+/-
KLRK
-
KLRC1-1
+
KLRC2-1
-
KLRC1-2
+
KLRC2-2
-
KLRC1-3
-
KLRC1-4
-
KLRC1-5
+/-
KLRC3
KLRJ
x
KLRL
-
KLRH1
+
KLRH2
(-)
KLRH4
KLRH
-
KLRA
-
KLRH3
+
KLRH2
-
KLRH3
+
KLRH2

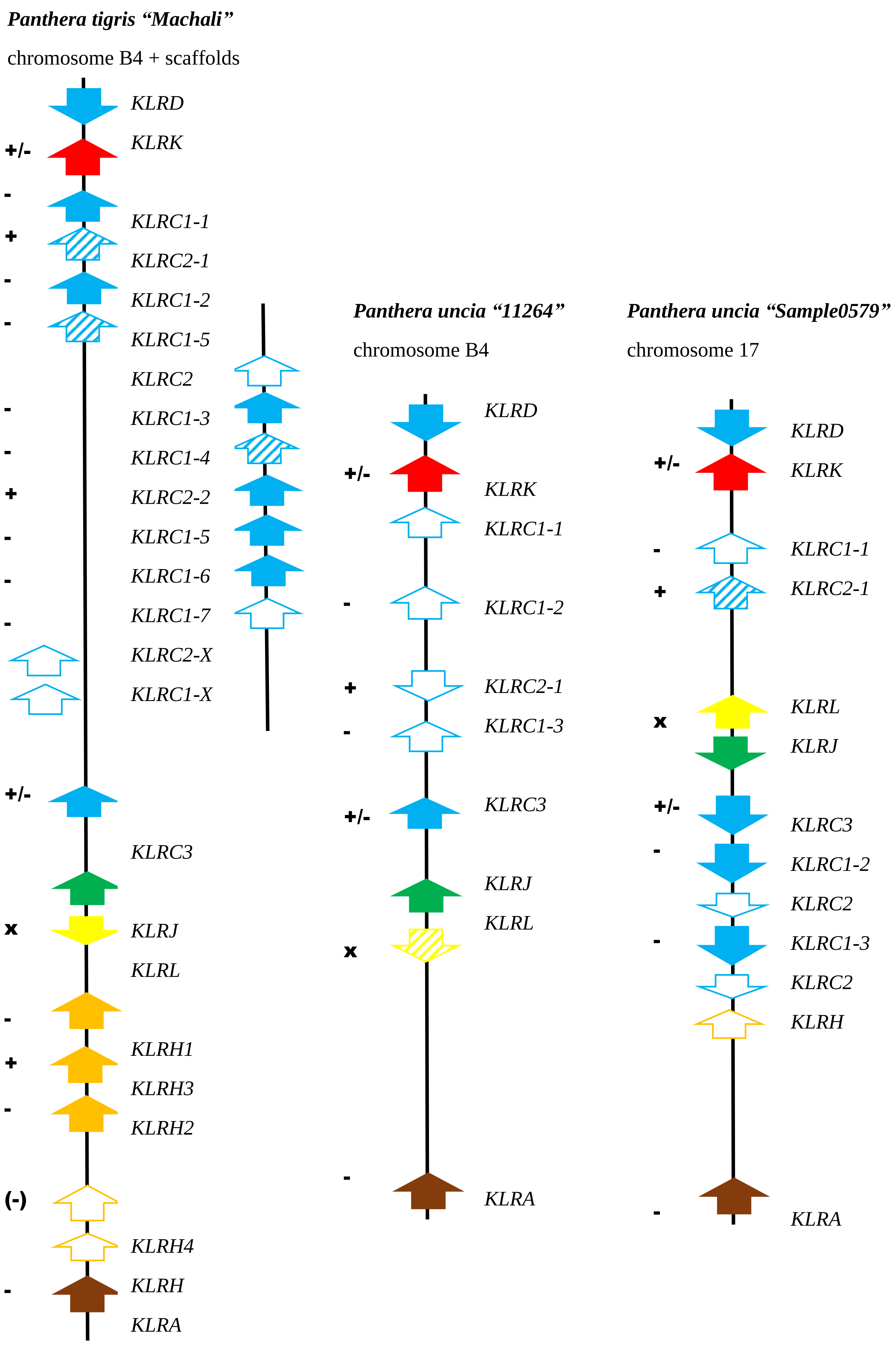
Panthera tigris "Machali"
chromosome B4 + scaffolds
KLRD
+/-
KLRK
-
KLRC1-1
+
KLRC2-1
-
KLRC1-2
-
KLRC1-5
KLRC2
-
KLRC1-3
-
KLRC1-4
+
KLRC2-2
-
KLRC1-5
-
KLRC1-6
-
KLRC1-7
KLRC2-X
KLRC1-X
+/-
KLRC3
x
KLRJ
KLRL
-
KLRH1
+
KLRH3
-
KLRH2
(-)
KLRH4
-
KLRH
KLRA
Panthera uncia "11264"
chromosome B4
KLRD
+/-
KLRK
KLRC1-1
-
KLRC1-2
+
KLRC2-1
-
KLRC1-3
+/-
KLRC3
KLRJ
KLRL
x
-
KLRA
Panthera uncia "Sample0579"
chromosome 17
KLRD
+/-
KLRK
-
KLRC1-1
+
KLRC2-1
x
KLRL
KLRJ
+/-
KLRC3
-
KLRC1-2
KLRC2
-
KLRC1-3
KLRC2
KLRH
-
KLRA

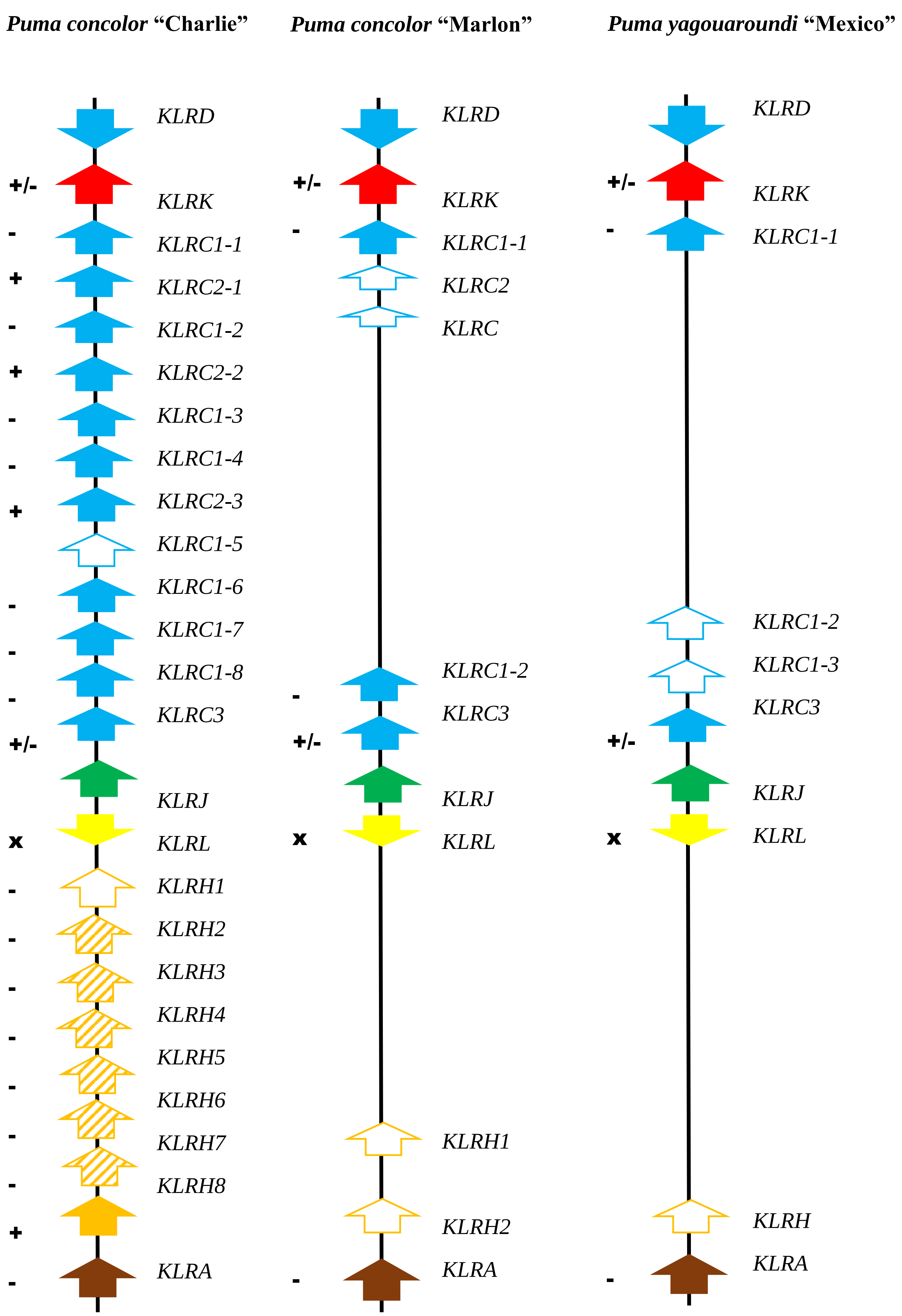
Puma concolor "Charlie"
Puma concolor "Marlon"
Puma yagouaroundi "Mexico"
KLRD
+/-
KLRK
-
KLRC1-1
+
KLRC2-1
-
KLRC1-2
+
KLRC2-2
-
KLRC1-3
-
KLRC1-4
+
KLRC2-3
KLRC1-5
KLRC1-6
-
KLRC1-7
-
KLRC1-8
-
KLRC3
+/-
KLRJ
x
KLRL
-
KLRH1
-
KLRH2
-
KLRH3
-
KLRH4
-
KLRH5
-
KLRH6
-
KLRH7
-
KLRH8
+
-
KLRA
KLRD
+/-
KLRK
-
KLRC1-1
KLRC2
KLRC
KLRC1-2
-
KLRC3
+/-
KLRJ
x
KLRL
KLRH1
KLRH2
KLRA
-
KLRD
+/-
KLRK
-
KLRC1-1
KLRC1-2
KLRC1-3
KLRC3
+/-
KLRJ
x
KLRL
KLRH
KLRA
-
