# Supplementary material for: Comparative genomics of the Natural Killer Complex in carnivores
Source: Front Immunol. 2024 Oct 3;15:1459122. doi: 10.3389/fimmu.2024.1459122 (PMC11484026; doi:10.3389/fimmu.2024.1459122)
Supplement: Supplementary file 5 [file Image2.pdf]

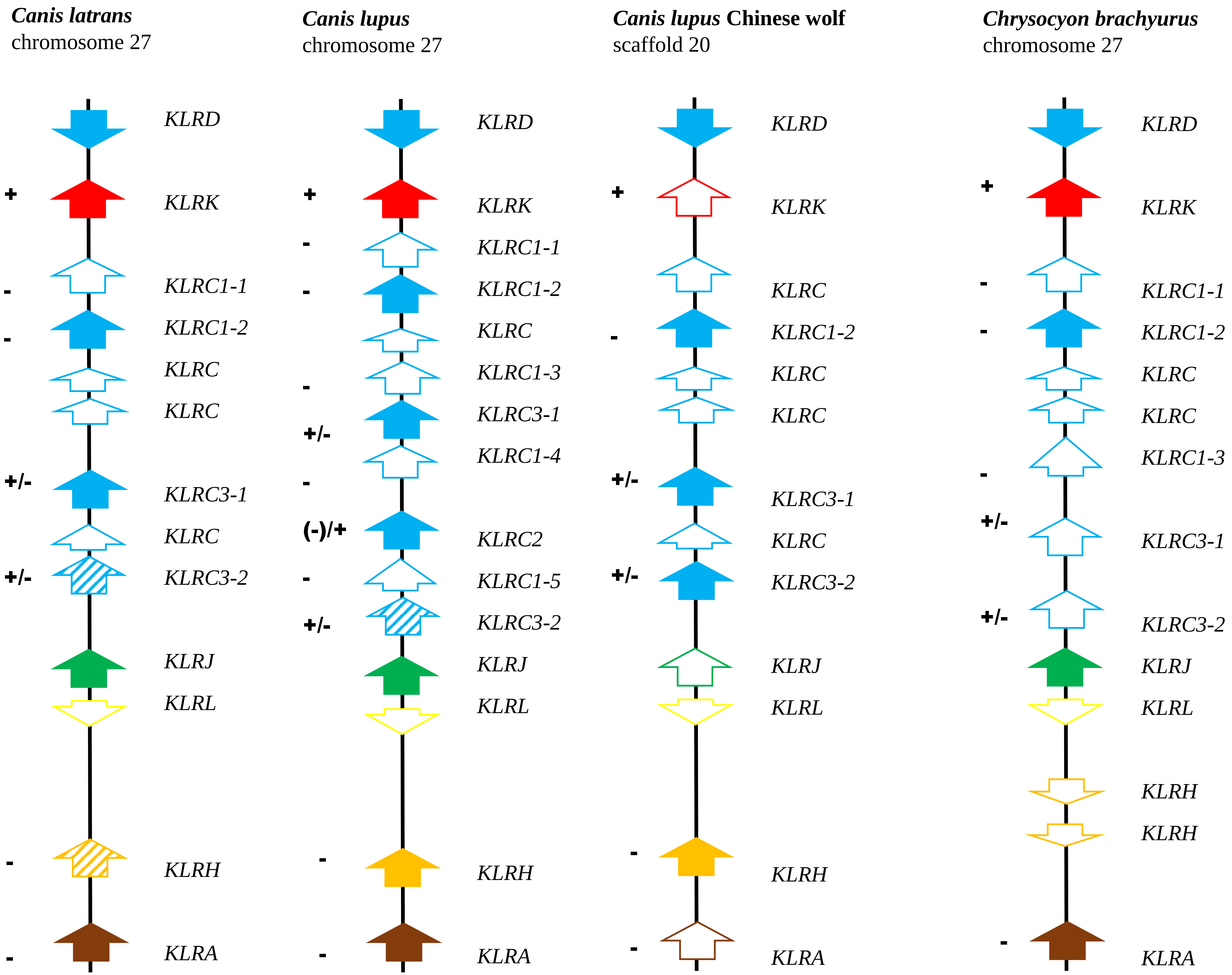
Canis latrans
chromosome 27
KLRD
+ KLRK
- KLRC1-1
- KLRC1-2
KLRC
KLRC
+/- KLRC3-1
KLRC
+/- KLRC3-2
KLRJ
KLRL
- KLRH
- KLRA
Canis lupus
chromosome 27
KLRD
+ KLRK
- KLRC1-1
- KLRC1-2
KLRC
- KLRC1-3
+/- KLRC3-1
- KLRC1-4
(-)/+ KLRC2
- KLRC1-5
+/- KLRC3-2
KLRJ
KLRL
- KLRH
- KLRA
Canis lupus Chinese wolf
scaffold 20
KLRD
+ KLRK
KLRC
- KLRC1-2
KLRC
KLRC
+/- KLRC3-1
KLRC
+/- KLRC3-2
KLRJ
KLRL
- KLRH
- KLRA
Chrysocyon brachyurus
chromosome 27
KLRD
+ KLRK
- KLRC1-1
- KLRC1-2
KLRC
KLRC
- KLRC1-3
+/- KLRC3-1
+/- KLRC3-2
KLRJ
KLRL
KLRH
KLRH
- KLRA

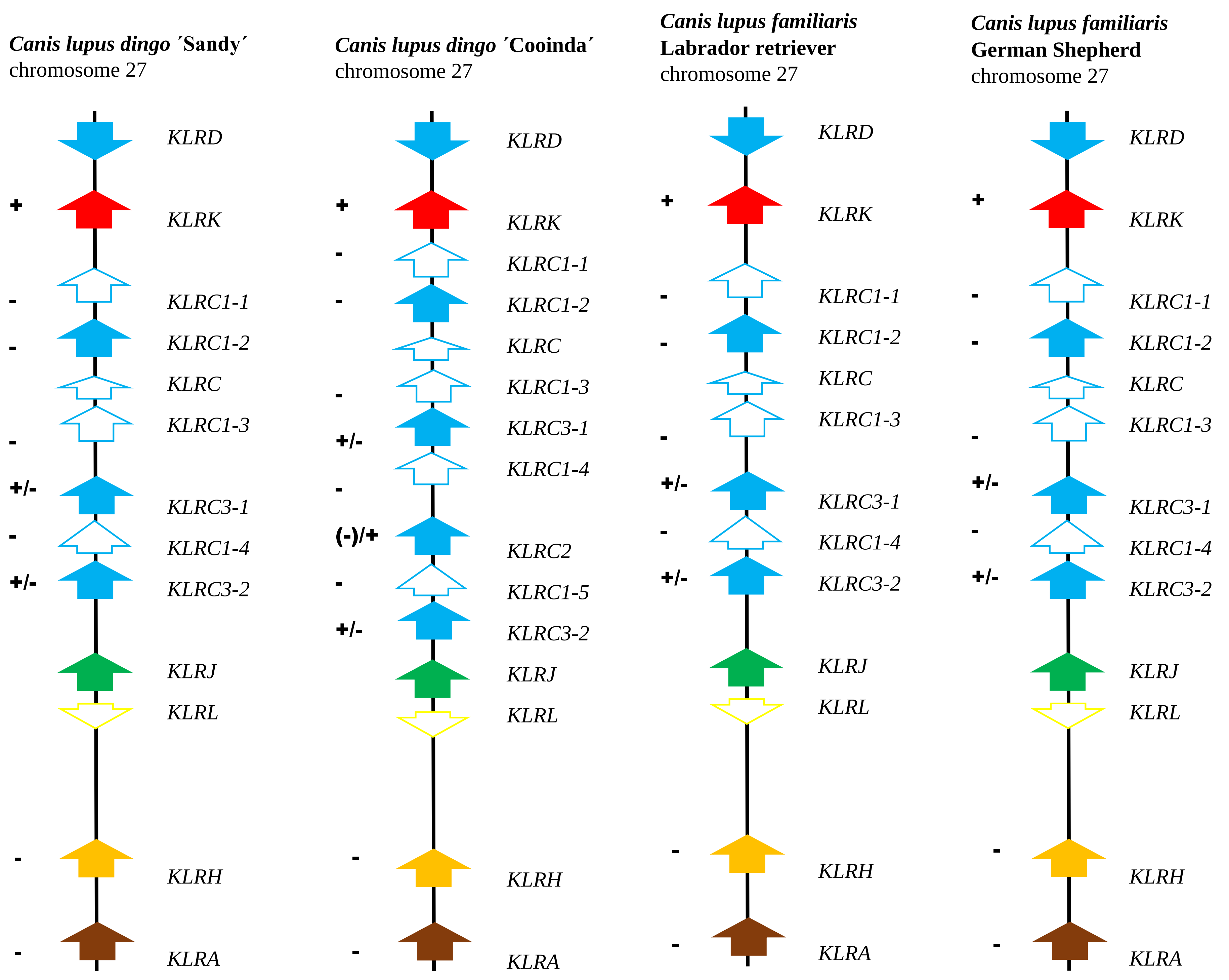

Canis lupus dingo ´Sandy´
chromosome 27
KLRD
+
KLRK
-
KLRC1-1
-
KLRC1-2
KLRC
KLRC1-3
-
+/-
KLRC3-1
-
KLRC1-4
+/-
KLRC3-2
KLRJ
KLRL
-
KLRH
-
KLRA
Canis lupus dingo ´Cooinda´
chromosome 27
KLRD
+
KLRK
-
KLRC1-1
-
KLRC1-2
KLRC
-
KLRC1-3
+/-
KLRC3-1
-
KLRC1-4
(-)/+
KLRC2
-
KLRC1-5
+/-
KLRC3-2
KLRJ
KLRL
-
KLRH
-
KLRA
Canis lupus familiaris
Labrador retriever
chromosome 27
KLRD
+
KLRK
-
KLRC1-1
-
KLRC1-2
KLRC
KLRC1-3
-
+/-
KLRC3-1
-
KLRC1-4
+/-
KLRC3-2
KLRJ
KLRL
-
KLRH
-
KLRA
Canis lupus familiaris
German Shepherd
chromosome 27
KLRD
+
KLRK
-
KLRC1-1
-
KLRC1-2
KLRC
KLRC1-3
-
+/-
KLRC3-1
-
KLRC1-4
+/-
KLRC3-2
KLRJ
KLRL
-
KLRH
-
KLRA

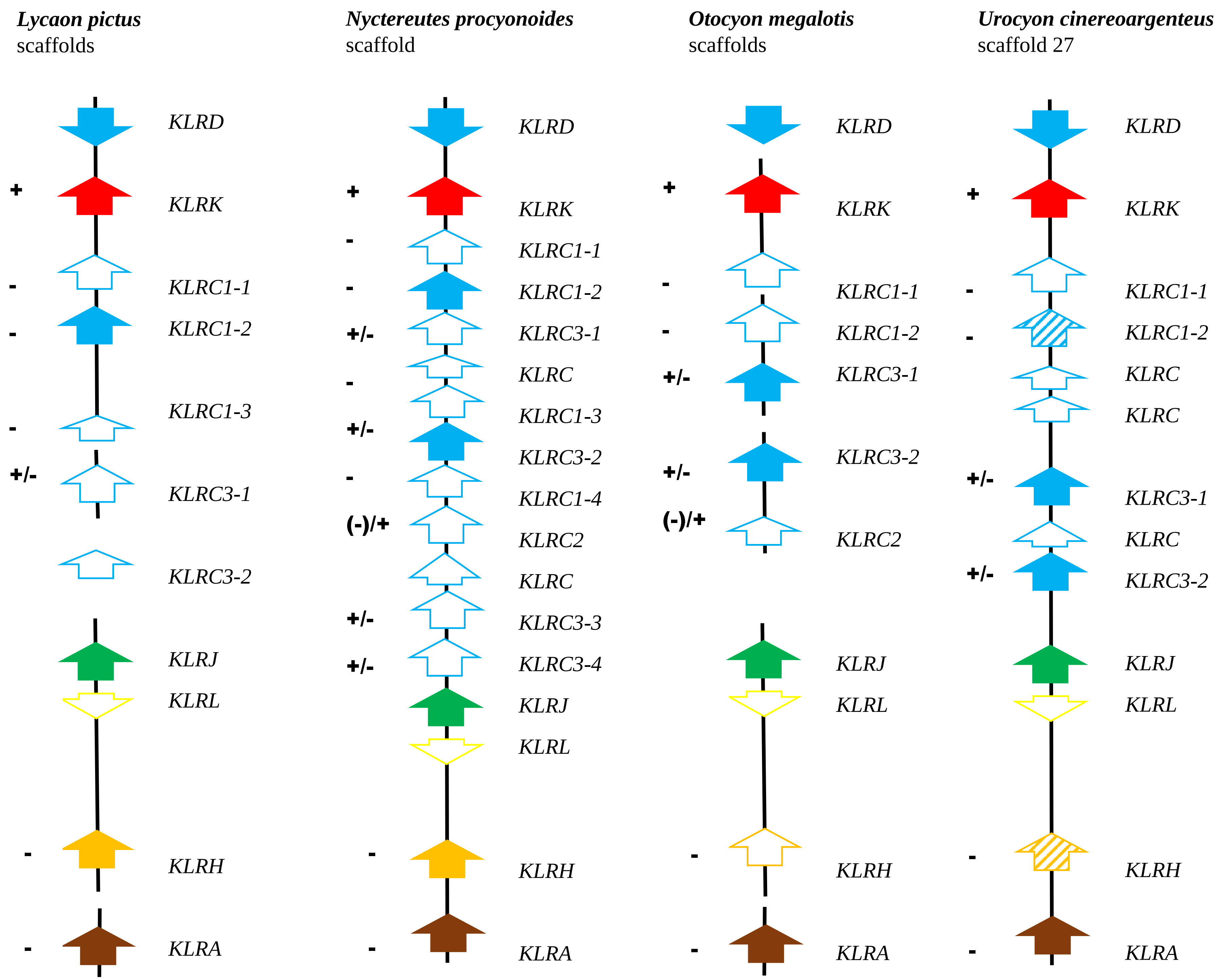

Lycaon pictus
scaffolds
KLRD
+
KLRK
-
KLRC1-1
-
KLRC1-2
-
KLRC1-3
+/-
KLRC3-1
KLRC3-2
KLRJ
KLRL
-
KLRH
-
KLRA
Nyctereutes procyonoides
scaffold
KLRD
+
KLRK
-
KLRC1-1
-
KLRC1-2
+/-
KLRC3-1
-
KLRC
KLRC1-3
+/-
KLRC3-2
-
KLRC1-4
(-)/+
KLRC2
KLRC
+/-
KLRC3-3
+/-
KLRC3-4
KLRJ
KLRL
-
KLRH
-
KLRA
Otocyon megalotis
scaffolds
KLRD
+
KLRK
-
KLRC1-1
-
KLRC1-2
+/-
KLRC3-1
+/-
KLRC3-2
(-)/+
KLRC2
KLRJ
KLRL
-
KLRH
-
KLRA
Urocyon cinereoargenteus
scaffold 27
KLRD
+
KLRK
-
KLRC1-1
-
KLRC1-2
KLRC
KLRC
+/-
KLRC3-1
KLRC
+/-
KLRC3-2
KLRJ
KLRL
-
KLRH
-
KLRA

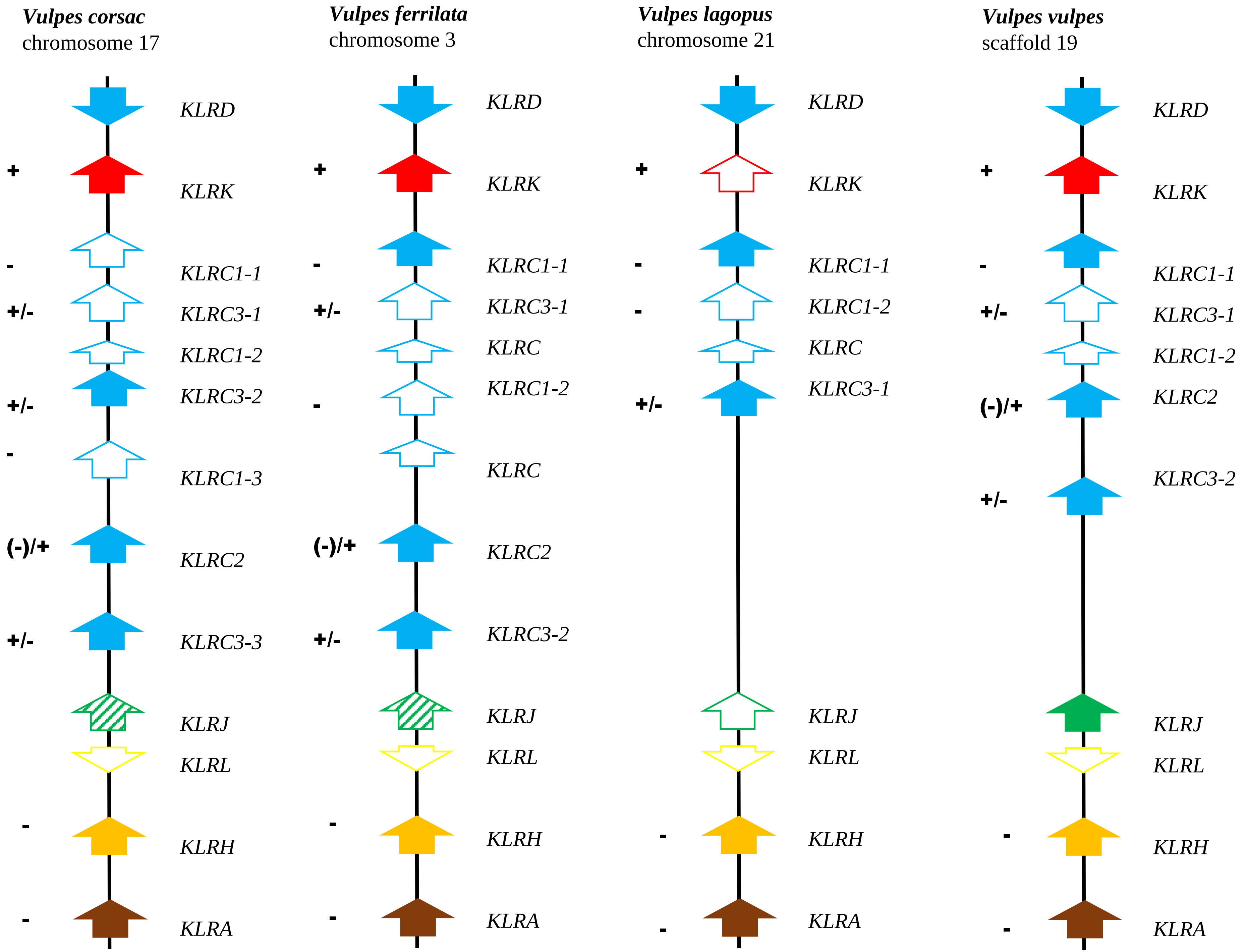

Vulpes corsac
chromosome 17
Vulpes ferrilata
chromosome 3
Vulpes lagopus
chromosome 21
Vulpes vulpes
scaffold 19
KLRD
+
KLRK
-
KLRC1-1
+/-
KLRC3-1
KLRC1-2
+/-
KLRC3-2
-
KLRC1-3
(-)/+
KLRC2
+/-
KLRC3-3
KLRJ
KLRL
-
KLRH
-
KLRA
KLRD
+
KLRK
-
KLRC1-1
+/-
KLRC3-1
KLRC
-
KLRC1-2
KLRC
(-)/+
KLRC2
+/-
KLRC3-2
KLRJ
KLRL
-
KLRH
-
KLRA
KLRD
+
KLRK
-
KLRC1-1
-
KLRC1-2
KLRC
+/-
KLRC3-1
KLRJ
KLRL
-
KLRH
-
KLRA
KLRD
+
KLRK
-
KLRC1-1
+/-
KLRC3-1
KLRC1-2
(-)/+
KLRC2
+/-
KLRC3-2
KLRJ
KLRL
-
KLRH
-
KLRA
