# Supplementary material for: Comparative genomics of the Natural Killer Complex in carnivores
Source: Front Immunol. 2024 Oct 3;15:1459122. doi: 10.3389/fimmu.2024.1459122 (PMC11484026; doi:10.3389/fimmu.2024.1459122)
Supplement: Supplementary file 6 [file Image3.pdf]

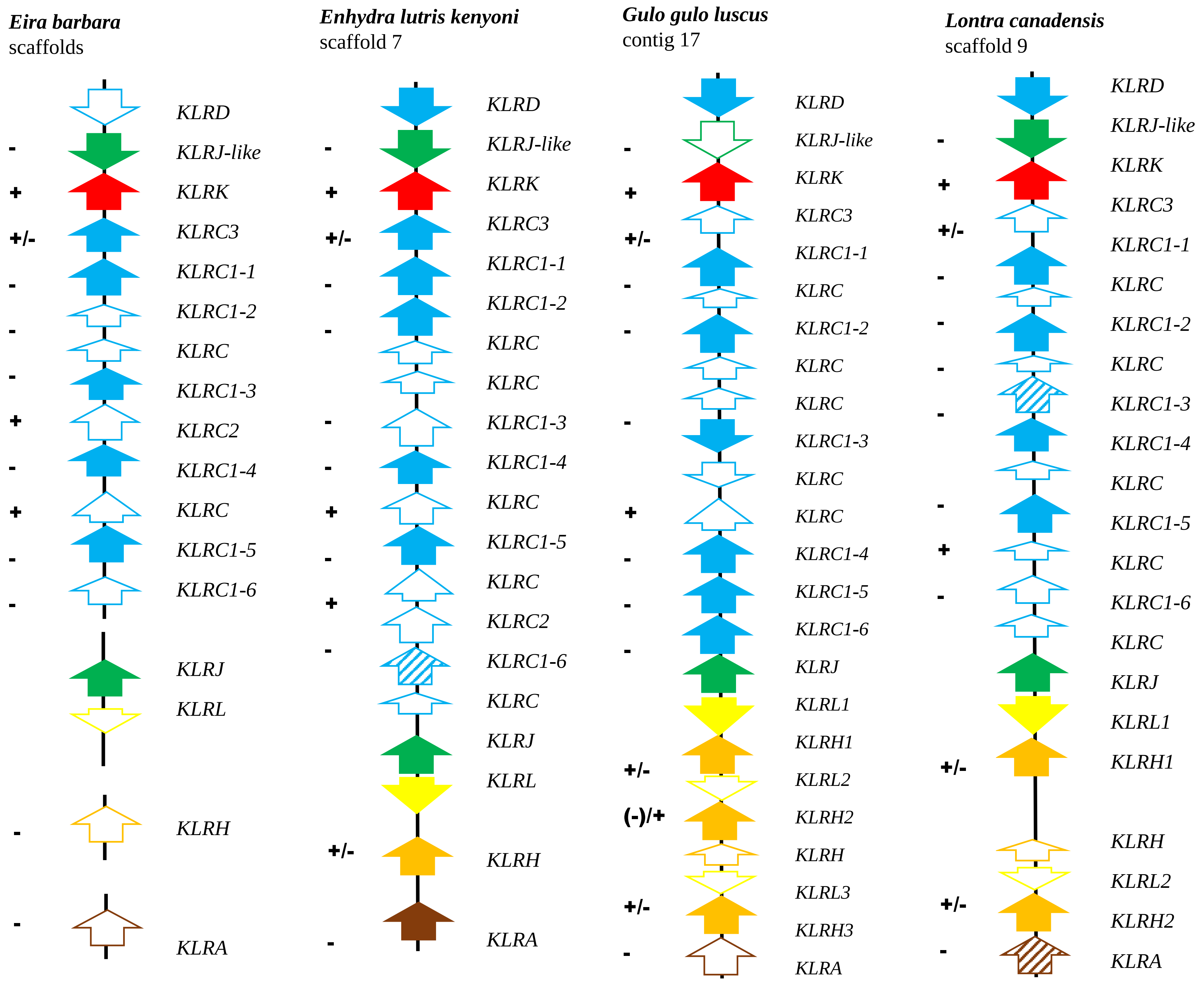

Eira barbara
scaffolds
KLRD
KLRJ-like
KLRK
KLRC3
KLRC1-1
KLRC1-2
KLRC
KLRC1-3
KLRC2
KLRC1-4
KLRC
KLRC1-5
KLRC1-6
KLRJ
KLRL
KLRH
KLRA
Enhydra lutris kenyoni
scaffold 7
KLRD
KLRJ-like
KLRK
KLRC3
KLRC1-1
KLRC1-2
KLRC
KLRC
KLRC1-3
KLRC1-4
KLRC
KLRC1-5
KLRC
KLRC2
KLRC1-6
KLRC
KLRJ
KLRL
KLRH
KLRA
Gulo gulo luscus
contig 17
KLRD
KLRJ-like
KLRK
KLRC3
KLRC1-1
KLRC
KLRC1-2
KLRC
KLRC
KLRC1-3
KLRC
KLRC
KLRC1-4
KLRC1-5
KLRC1-6
KLRJ
KLRL1
KLRH1
KLRL2
KLRH2
KLRH
KLRL3
KLRH3
KLRA
(-)/+
Lontra canadensis
scaffold 9
KLRD
KLRJ-like
KLRK
KLRC3
KLRC1-1
KLRC
KLRC1-2
KLRC
KLRC1-3
KLRC1-4
KLRC
KLRC1-5
KLRC
KLRC1-6
KLRC
KLRJ
KLRL1
KLRH1
KLRH
KLRL2
KLRH2
KLRA

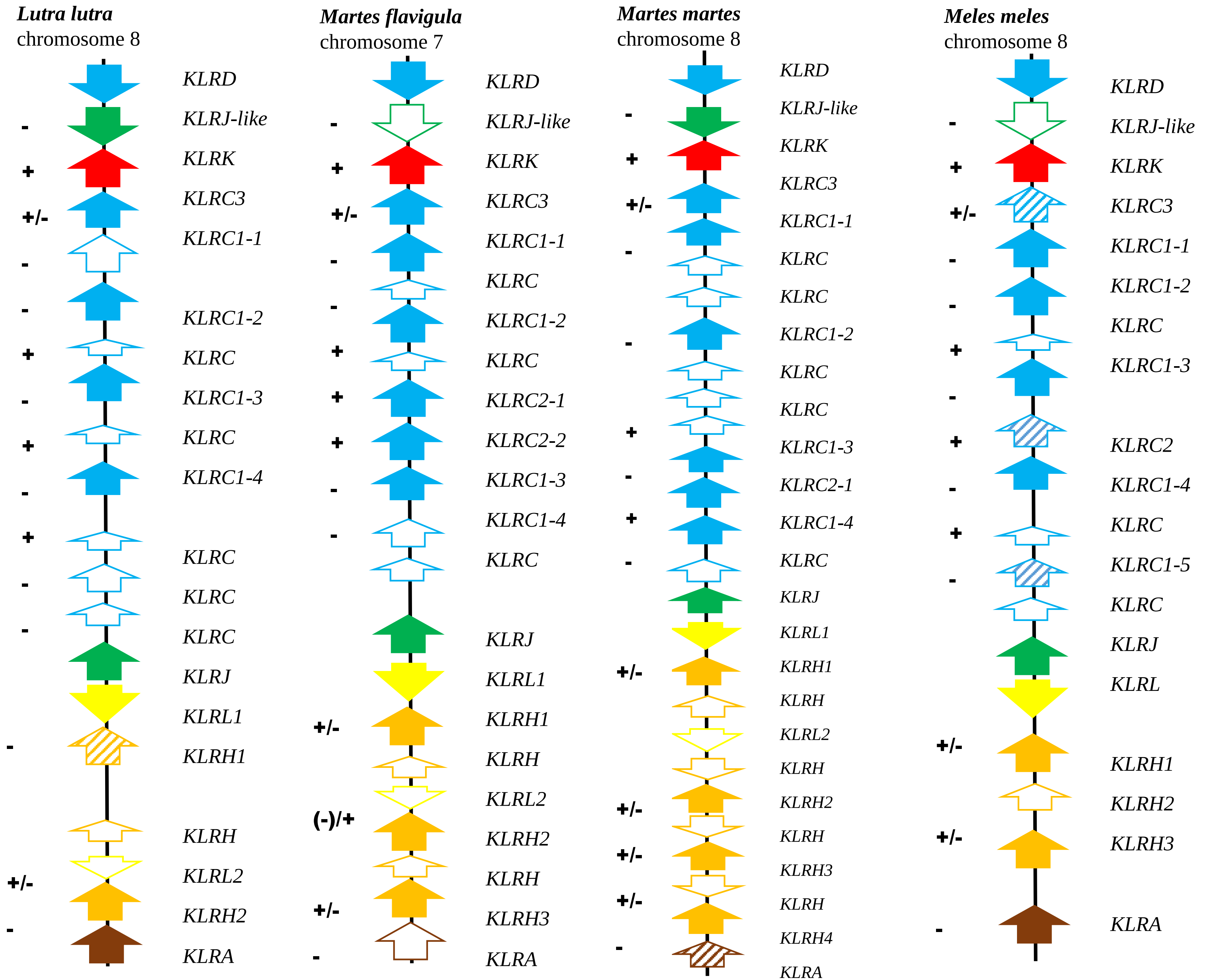
Lutra lutra
chromosome 8
KLRD
KLRJ-like
KLRK
KLRC3
KLRC1-1
KLRC1-2
KLRC
KLRC1-3
KLRC
KLRC1-4
KLRC
KLRC
KLRC
KLRJ
KLRL1
KLRH1
KLRH
KLRL2
KLRH2
KLRA
-
+
+/-
-
-
+
-
+
-
+
-
-
-
+/-
-
Martes flavigula
chromosome 7
KLRD
KLRJ-like
KLRK
KLRC3
KLRC1-1
KLRC
KLRC1-2
KLRC
KLRC2-1
KLRC2-2
KLRC1-3
KLRC1-4
KLRC
KLRJ
KLRL1
KLRH1
KLRH
KLRL2
KLRH2
KLRH
KLRH3
KLRA
-
+
+/-
-
-
+
+
+
-
-
+/-
(-)/+
+/-
-
Martes martes
chromosome 8
KLRD
KLRJ-like
KLRK
KLRC3
KLRC1-1
KLRC
KLRC
KLRC1-2
KLRC
KLRC
KLRC1-3
KLRC2-1
KLRC1-4
KLRC
KLRJ
KLRL1
KLRH1
KLRH
KLRL2
KLRH
KLRH2
KLRH
KLRH3
KLRH
KLRH4
KLRA
-
+
+/-
-
-
+
-
+
-
+/-
+/-
+/-
+/-
-
Meles meles
chromosome 8
KLRD
KLRJ-like
KLRK
KLRC3
KLRC1-1
KLRC1-2
KLRC
KLRC1-3
KLRC2
KLRC1-4
KLRC
KLRC1-5
KLRC
KLRJ
KLRL
KLRH1
KLRH2
KLRH3
KLRA
-
+
+/-
-
-
+
-
+
-
+
-
+/-
+/-
-

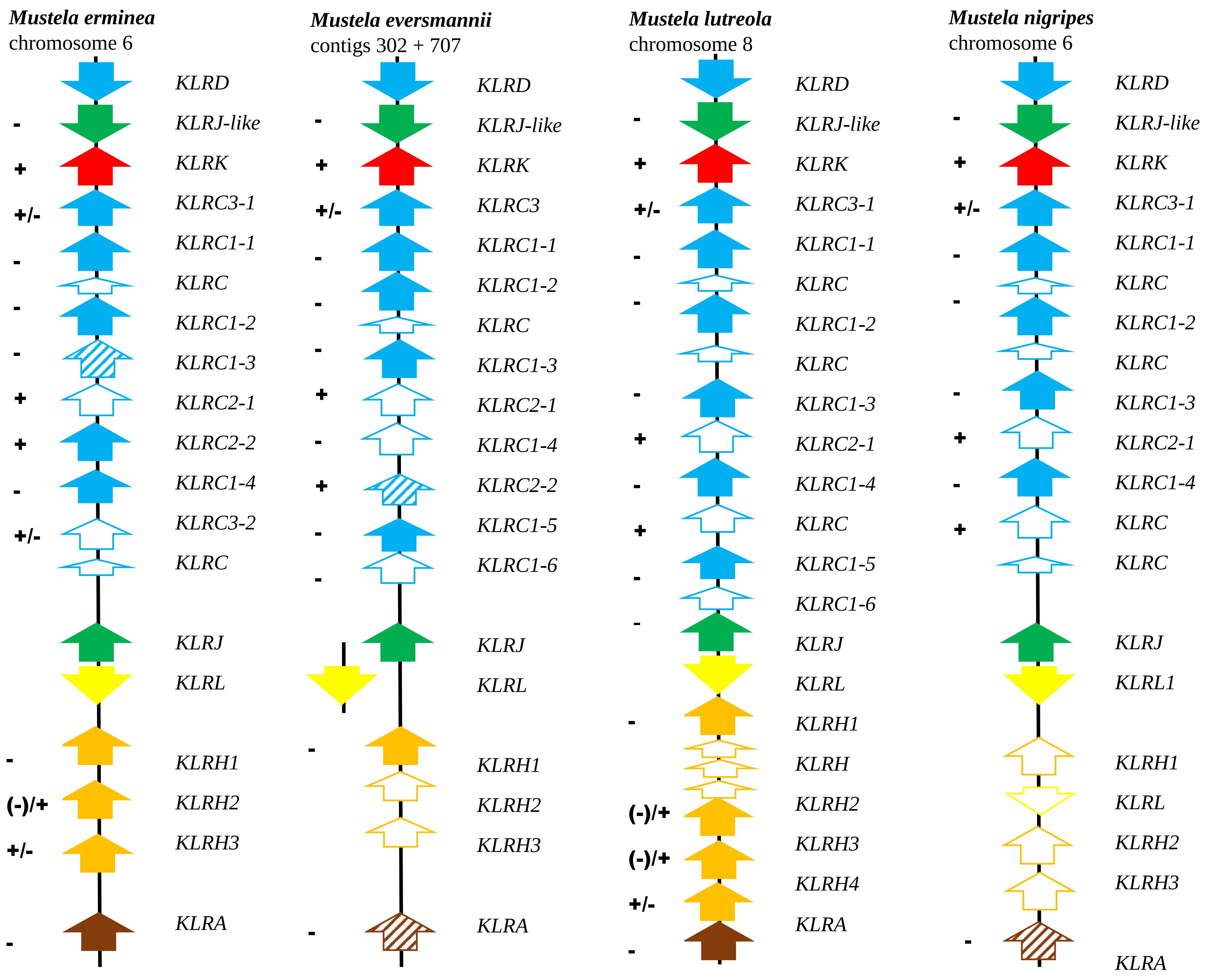

Mustela erminea
chromosome 6
KLRD
KLRJ-like
KLRK
KLRC3-1
KLRC1-1
KLRC
KLRC1-2
KLRC1-3
KLRC2-1
KLRC2-2
KLRC1-4
KLRC3-2
KLRC
KLRJ
KLRL
KLRH1
KLRH2
KLRH3
KLRA
Mustela eversmannii
contigs 302 + 707
KLRD
KLRJ-like
KLRK
KLRC3
KLRC1-1
KLRC1-2
KLRC
KLRC1-3
KLRC2-1
KLRC1-4
KLRC2-2
KLRC1-5
KLRC1-6
KLRJ
KLRL
KLRH1
KLRH2
KLRH3
KLRA
Mustela lutreola
chromosome 8
KLRD
KLRJ-like
KLRK
KLRC3-1
KLRC1-1
KLRC
KLRC1-2
KLRC
KLRC1-3
KLRC2-1
KLRC1-4
KLRC
KLRC1-5
KLRC1-6
KLRJ
KLRL
KLRH1
KLRH
KLRH2
KLRH3
KLRH4
KLRA
Mustela nigripes
chromosome 6
KLRD
KLRJ-like
KLRK
KLRC3-1
KLRC1-1
KLRC
KLRC1-2
KLRC
KLRC1-3
KLRC2-1
KLRC1-4
KLRC
KLRC
KLRJ
KLRL1
KLRH1
KLRL
KLRH2
KLRH3
KLRA

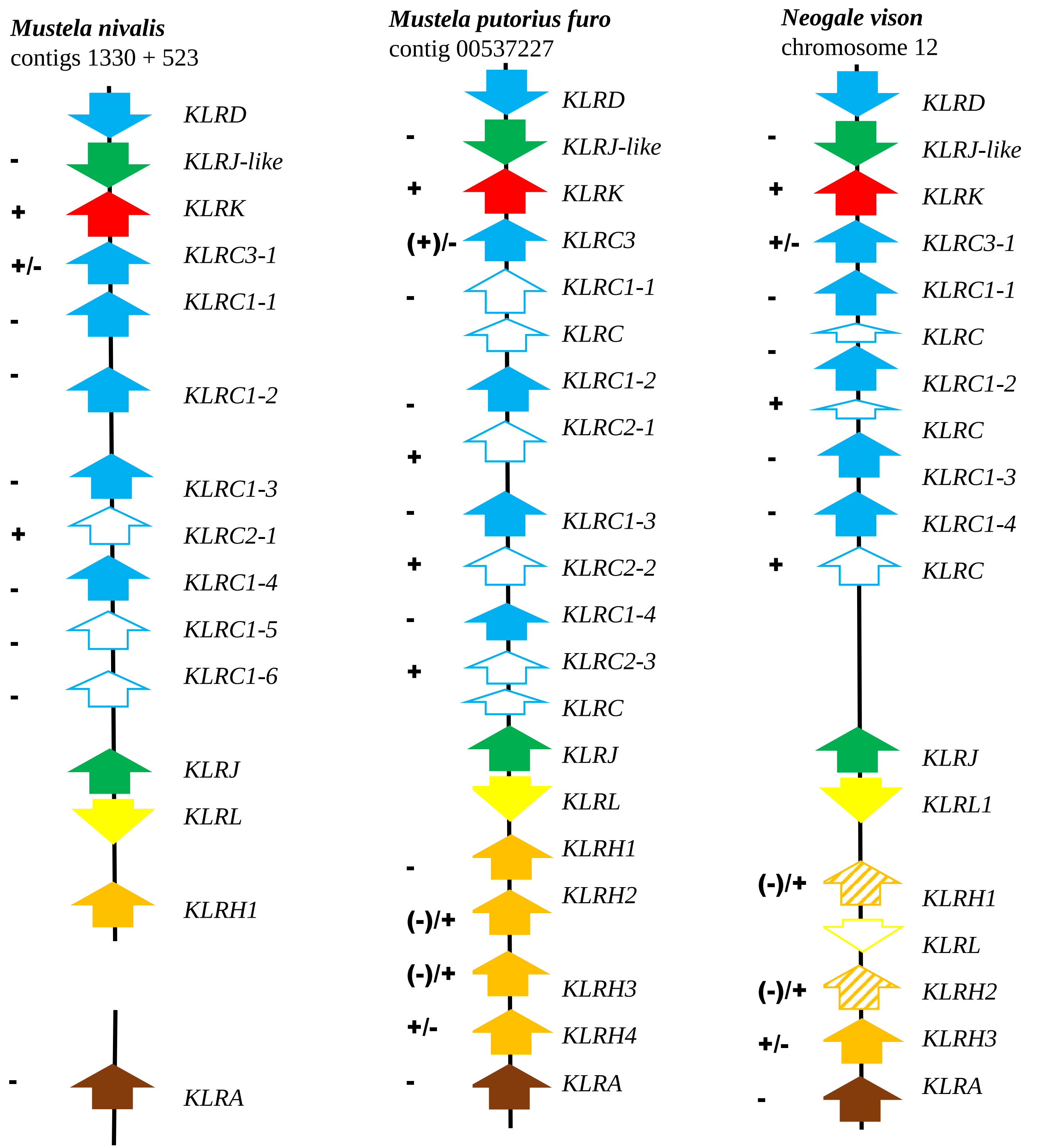

Mustela nivalis
contigs 1330 + 523
KLRD
- KLRJ-like
+ KLRK
+/- KLRC3-1
- KLRC1-1
- KLRC1-2
- KLRC1-3
+ KLRC2-1
- KLRC1-4
- KLRC1-5
- KLRC1-6
KLRJ
KLRL
KLRH1
- KLRA
Mustela putorius furo
contig 00537227
KLRD
- KLRJ-like
+ KLRK
(+)/- KLRC3
- KLRC1-1
KLRC
- KLRC1-2
KLRC2-1
+
- KLRC1-3
+ KLRC2-2
- KLRC1-4
+ KLRC2-3
KLRC
KLRJ
KLRL
KLRH1
- KLRH2
(-)/+
(-)/+ KLRH3
+/- KLRH4
- KLRA
Neogale vison
chromosome 12
KLRD
- KLRJ-like
+ KLRK
+/- KLRC3-1
- KLRC1-1
KLRC
- KLRC1-2
+ KLRC
- KLRC1-3
- KLRC1-4
+ KLRC
KLRJ
KLRL1
(-)/+ KLRH1
KLRL
(-)/+ KLRH2
+/- KLRH3
- KLRA
