# Supplementary material for: Comparative genomics of the Natural Killer Complex in carnivores
Source: Front Immunol. 2024 Oct 3;15:1459122. doi: 10.3389/fimmu.2024.1459122 (PMC11484026; doi:10.3389/fimmu.2024.1459122)
Supplement: Supplementary file 7 [file Image4.pdf]

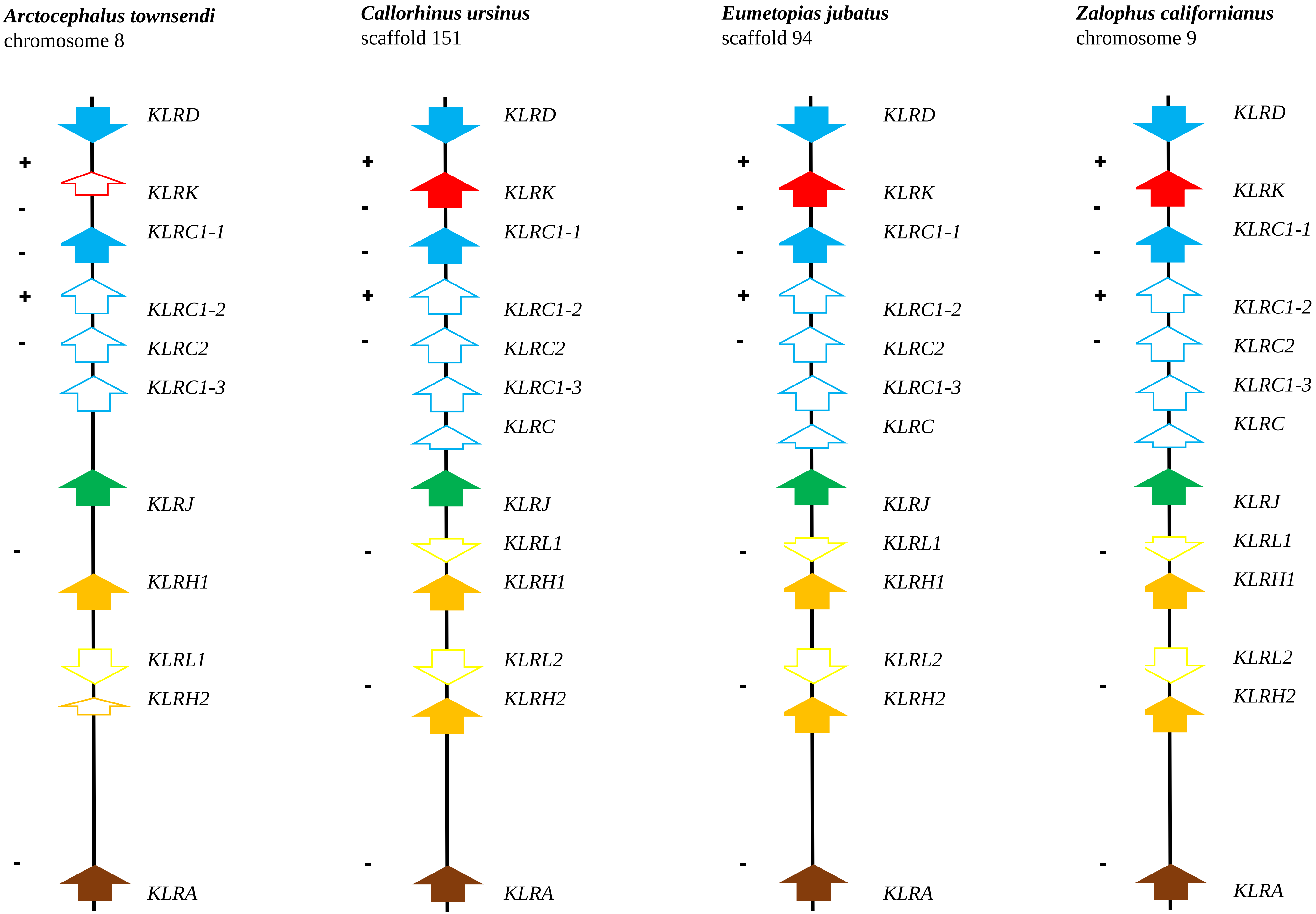
Arctocephalus townsendi
chromosome 8
KLRD
+
KLRK
-
KLRC1-1
-
+
KLRC1-2
-
KLRC2
KLRC1-3
KLRJ
-
KLRH1
KLRL1
KLRH2
-
KLRA
Callorhinus ursinus
scaffold 151
KLRD
+
KLRK
-
KLRC1-1
-
+
KLRC1-2
-
KLRC2
KLRC1-3
KLRC
KLRJ
-
KLRL1
KLRH1
-
KLRL2
KLRH2
-
KLRA
Eumetopias jubatus
scaffold 94
KLRD
+
KLRK
-
KLRC1-1
-
+
KLRC1-2
-
KLRC2
KLRC1-3
KLRC
KLRJ
-
KLRL1
KLRH1
-
KLRL2
KLRH2
-
KLRA
Zalophus californianus
chromosome 9
KLRD
+
KLRK
-
KLRC1-1
-
+
KLRC1-2
-
KLRC2
KLRC1-3
KLRC
KLRJ
-
KLRL1
KLRH1
-
KLRL2
KLRH2
-
KLRA
