# Supplementary material for: Comparative genomics of the Natural Killer Complex in carnivores
Source: Front Immunol. 2024 Oct 3;15:1459122. doi: 10.3389/fimmu.2024.1459122 (PMC11484026; doi:10.3389/fimmu.2024.1459122)
Supplement: Supplementary file 8 [file Image5.pdf]

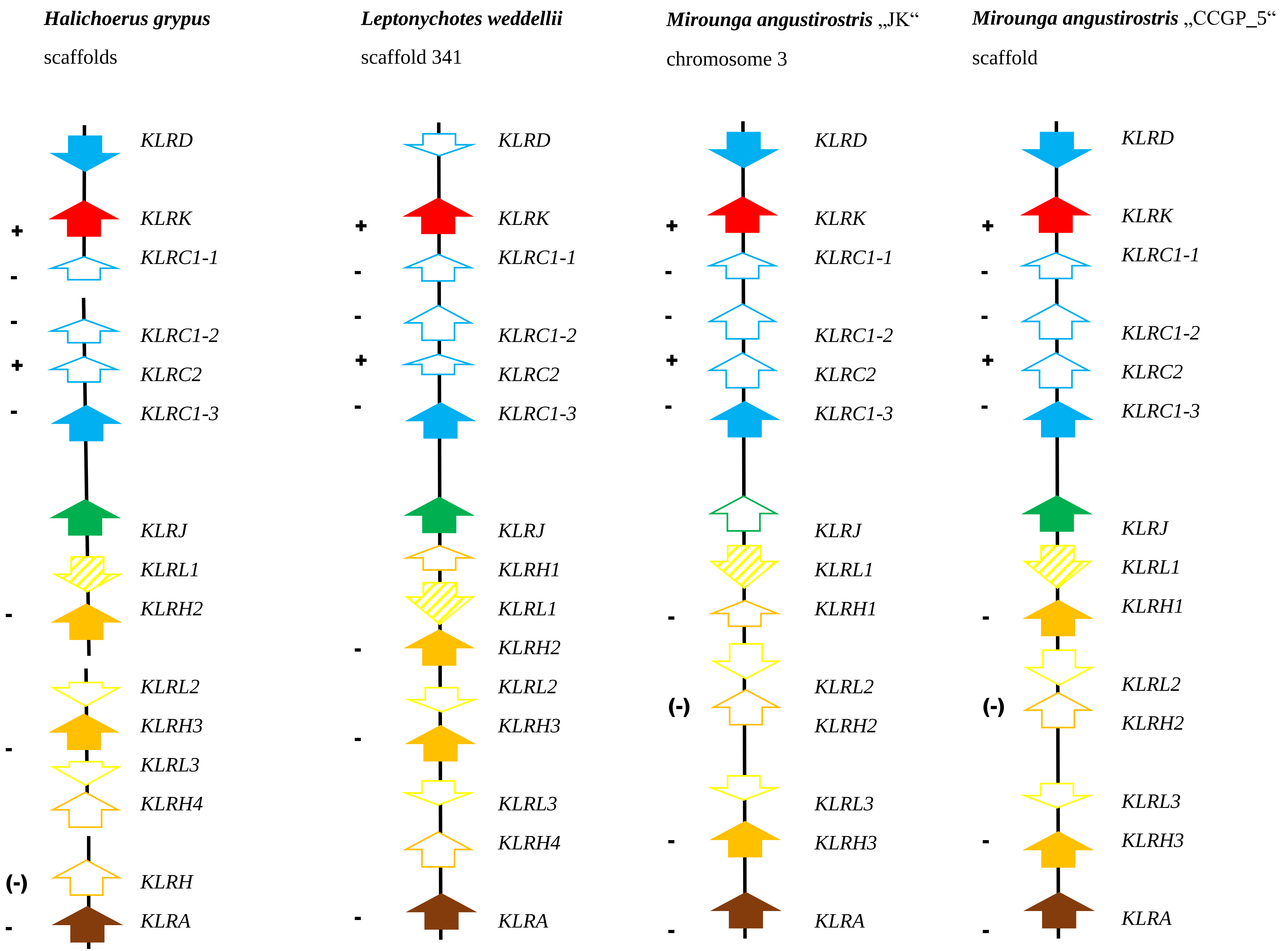
Halichoerus grypus
scaffolds
KLRD
KLRK
KLRC1-1
KLRC1-2
KLRC2
KLRC1-3
KLRJ
KLRL1
KLRH2
KLRL2
KLRH3
KLRL3
KLRH4
KLRH
KLRA
Leptonychotes weddellii
scaffold 341
KLRD
KLRK
KLRC1-1
KLRC1-2
KLRC2
KLRC1-3
KLRJ
KLRH1
KLRL1
KLRH2
KLRL2
KLRH3
KLRL3
KLRH4
KLRA
Mirounga angustirostris „JK“
chromosome 3
KLRD
KLRK
KLRC1-1
KLRC1-2
KLRC2
KLRC1-3
KLRJ
KLRL1
KLRH1
KLRL2
KLRH2
KLRL3
KLRH3
KLRA
Mirounga angustirostris „CCGP_5“
scaffold
KLRD
KLRK
KLRC1-1
KLRC1-2
KLRC2
KLRC1-3
KLRJ
KLRL1
KLRH1
KLRL2
KLRH2
KLRL3
KLRH3
KLRA

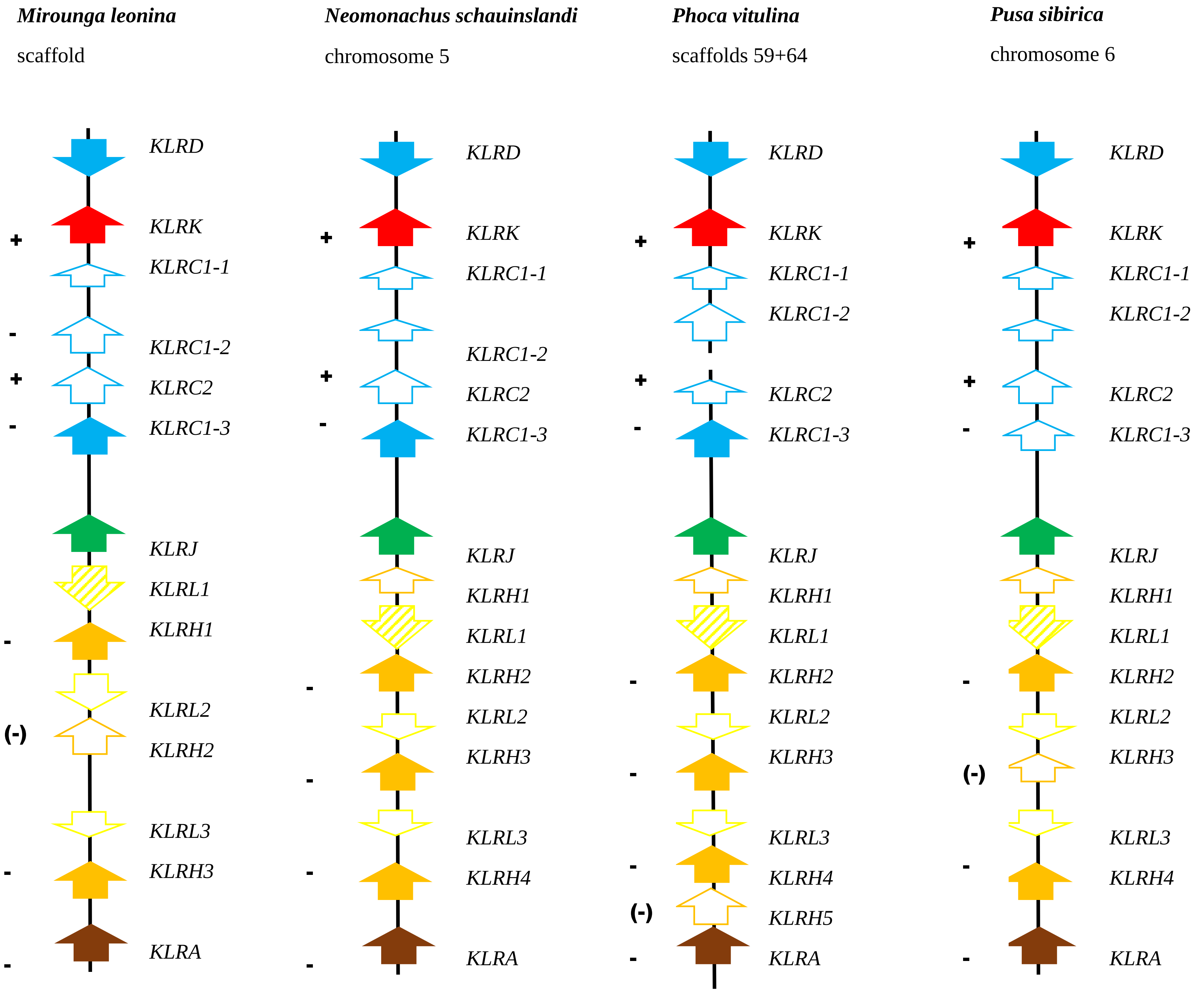
Mirounga leonina
scaffold
Neomonachus schauinslandi
chromosome 5
Phoca vitulina
scaffolds 59+64
Pusa sibirica
chromosome 6
KLRD
KLRK
KLRC1-1
KLRC1-2
KLRC2
KLRC1-3
KLRJ
KLRL1
KLRH1
KLRL2
KLRH2
KLRL3
KLRH3
KLRA
KLRD
KLRK
KLRC1-1
KLRC1-2
KLRC2
KLRC1-3
KLRJ
KLRH1
KLRL1
KLRH2
KLRL2
KLRH3
KLRL3
KLRH4
KLRA
KLRD
KLRK
KLRC1-1
KLRC1-2
KLRC2
KLRC1-3
KLRJ
KLRH1
KLRL1
KLRH2
KLRL2
KLRH3
KLRL3
KLRH4
KLRH5
KLRA
KLRD
KLRK
KLRC1-1
KLRC1-2
KLRC2
KLRC1-3
KLRJ
KLRH1
KLRL1
KLRH2
KLRL2
KLRH3
KLRL3
KLRH4
KLRA
+
-
(-)
