# Supplementary material for: Comparative genomics of the Natural Killer Complex in carnivores
Source: Front Immunol. 2024 Oct 3;15:1459122. doi: 10.3389/fimmu.2024.1459122 (PMC11484026; doi:10.3389/fimmu.2024.1459122)
Supplement: Supplementary file 9 [file Image6.pdf]

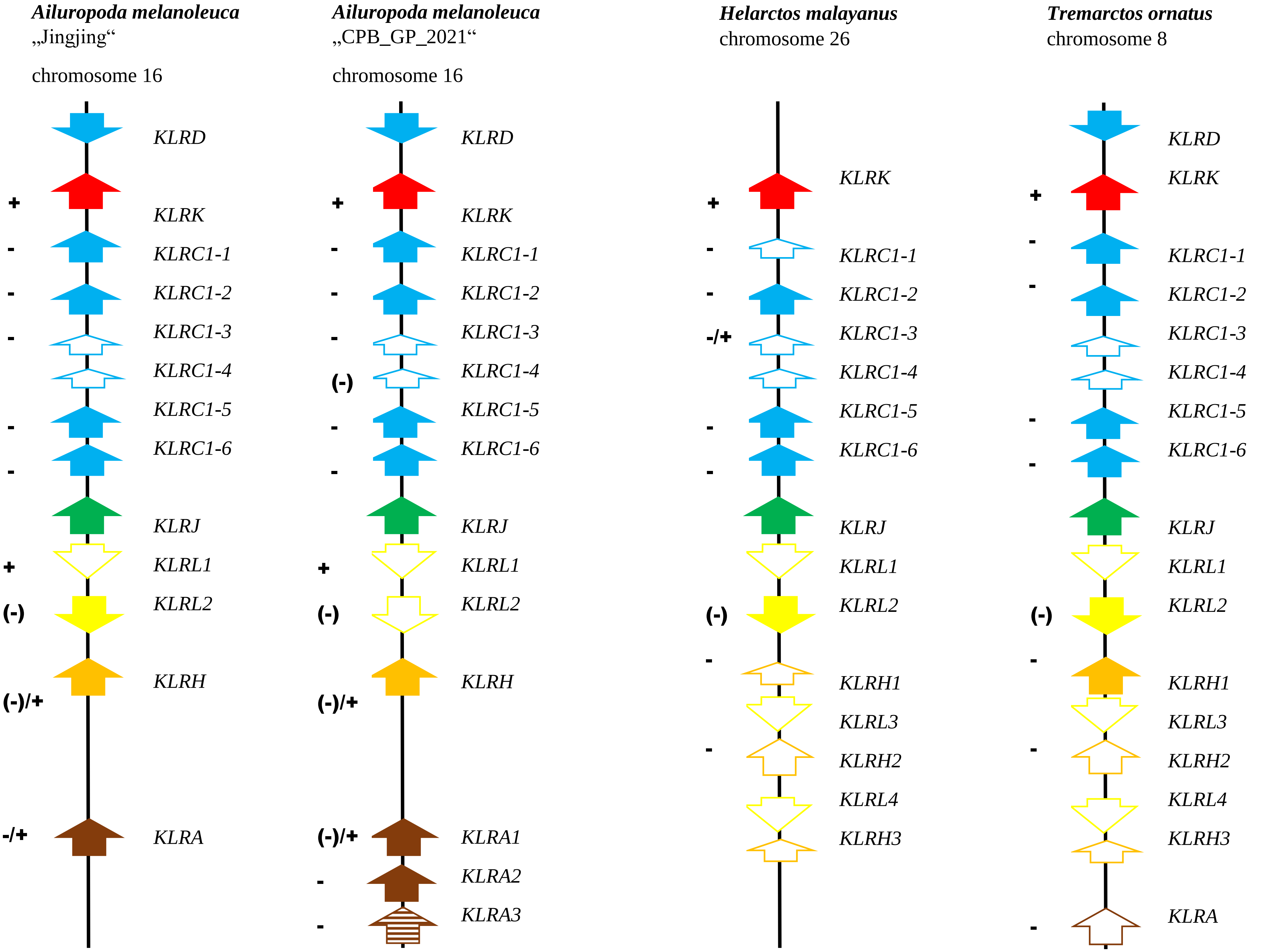

Ailuropoda melanoleuca „Jingjing“ chromosome 16
KLRD
+ KLRK
- KLRC1-1
- KLRC1-2
- KLRC1-3
KLRC1-4
- KLRC1-5
- KLRC1-6
KLRJ
+ KLRL1
(-) KLRL2
(-)/+ KLRH
-/+ KLRA
Ailuropoda melanoleuca „CPB_GP_2021“ chromosome 16
KLRD
+ KLRK
- KLRC1-1
- KLRC1-2
- KLRC1-3
(-) KLRC1-4
- KLRC1-5
- KLRC1-6
KLRJ
+ KLRL1
(-) KLRL2
(-)/+ KLRH
(-)/+ KLRA1
- KLRA2
- KLRA3
Helarctos malayanus chromosome 26
+ KLRK
- KLRC1-1
- KLRC1-2
-/+ KLRC1-3
KLRC1-4
- KLRC1-5
- KLRC1-6
KLRJ
KLRL1
(-) KLRL2
- KLRH1
KLRL3
- KLRH2
KLRL4
KLRH3
Tremarctos ornatus chromosome 8
KLRD
+ KLRK
- KLRC1-1
- KLRC1-2
KLRC1-3
KLRC1-4
- KLRC1-5
- KLRC1-6
KLRJ
KLRL1
(-) KLRL2
- KLRH1
KLRL3
- KLRH2
KLRL4
KLRH3
- KLRA

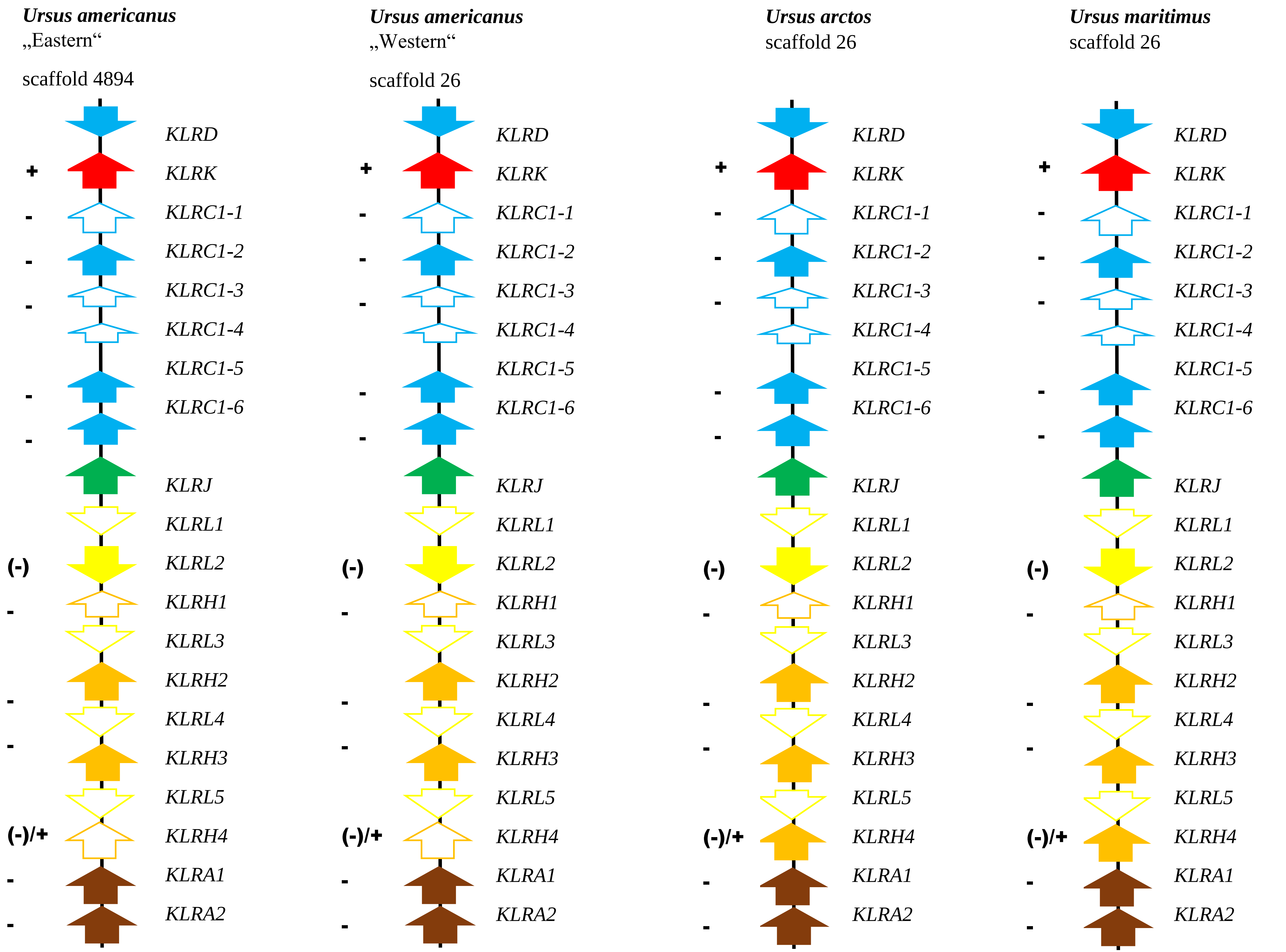

Ursus americanus
„Eastern“
scaffold 4894
Ursus americanus
„Western“
scaffold 26
Ursus arctos
scaffold 26
Ursus maritimus
scaffold 26
KLRD
KLRK
KLRC1-1
KLRC1-2
KLRC1-3
KLRC1-4
KLRC1-5
KLRC1-6
KLRJ
KLRL1
KLRL2
KLRH1
KLRL3
KLRH2
KLRL4
KLRH3
KLRL5
KLRH4
KLRA1
KLRA2
+
-
(-)
(-)/+
