# Supplementary figures and images for: Comparative genomics of the Natural Killer Complex in carnivores
Source: Front Immunol. 2024 Oct 3;15:1459122. doi: 10.3389/fimmu.2024.1459122 (PMC11484026; doi:10.3389/fimmu.2024.1459122)

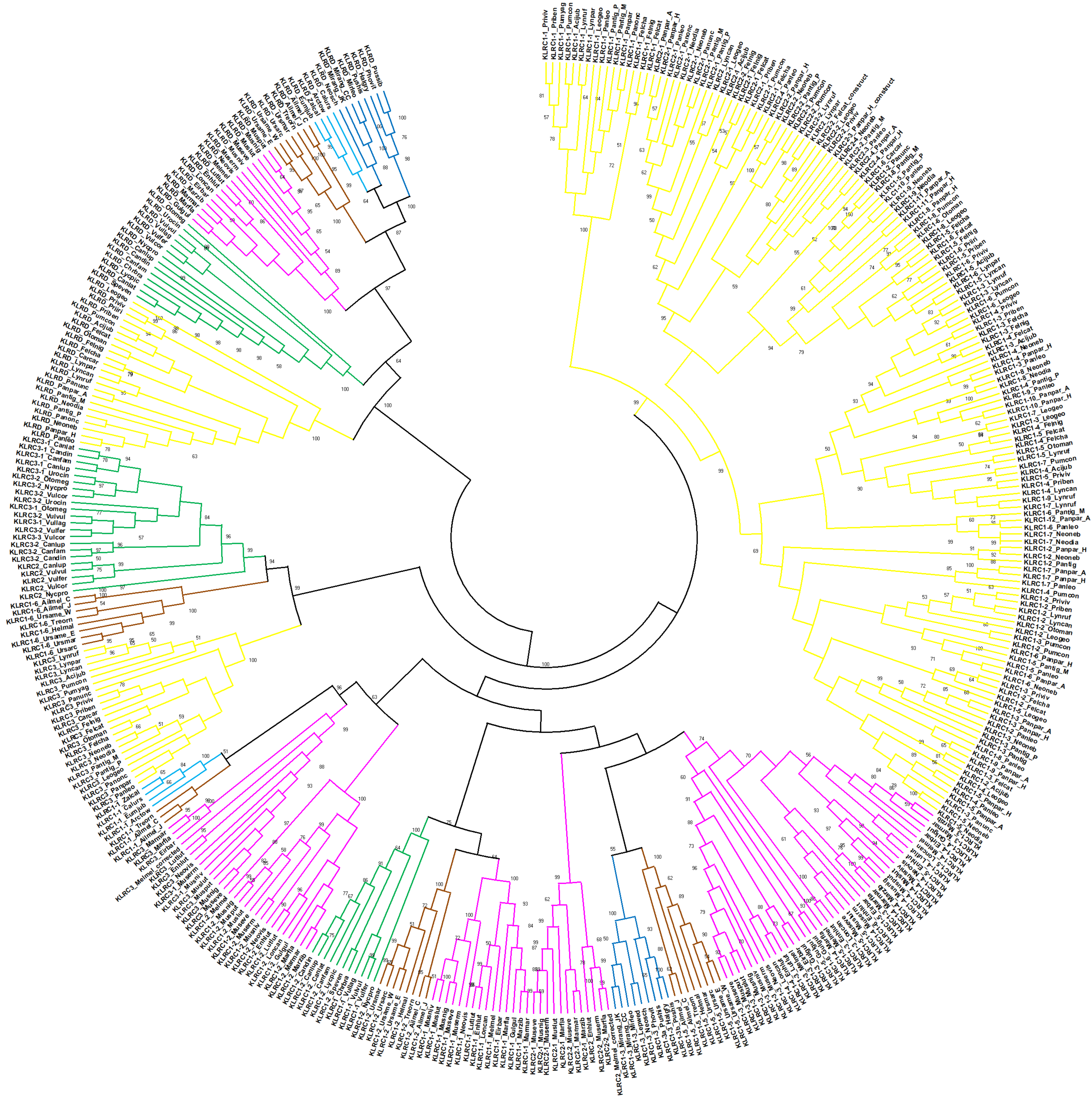

Supplement: Supplementary file 10 [file Image7.pdf]

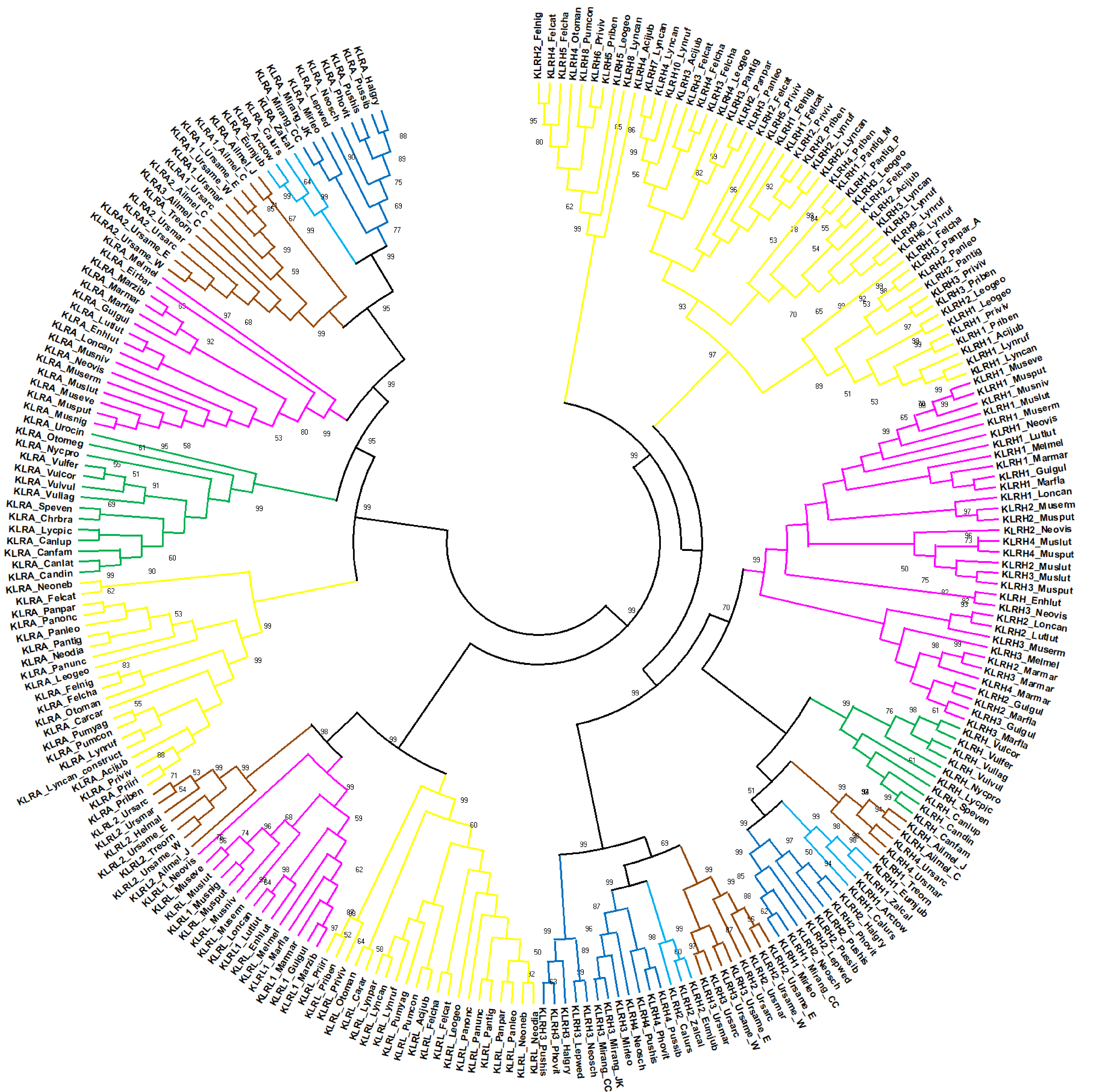

Supplement: Supplementary file 11 [file Image8.pdf]
