# Supplementary material for: Comparative genomics of the Natural Killer Complex in carnivores
Source: Front Immunol. 2024 Oct 3;15:1459122. doi: 10.3389/fimmu.2024.1459122 (PMC11484026; doi:10.3389/fimmu.2024.1459122)

CPB_GP_2021 Ailuropoda_melanoleuca_CPB_GP_2021:1-188001

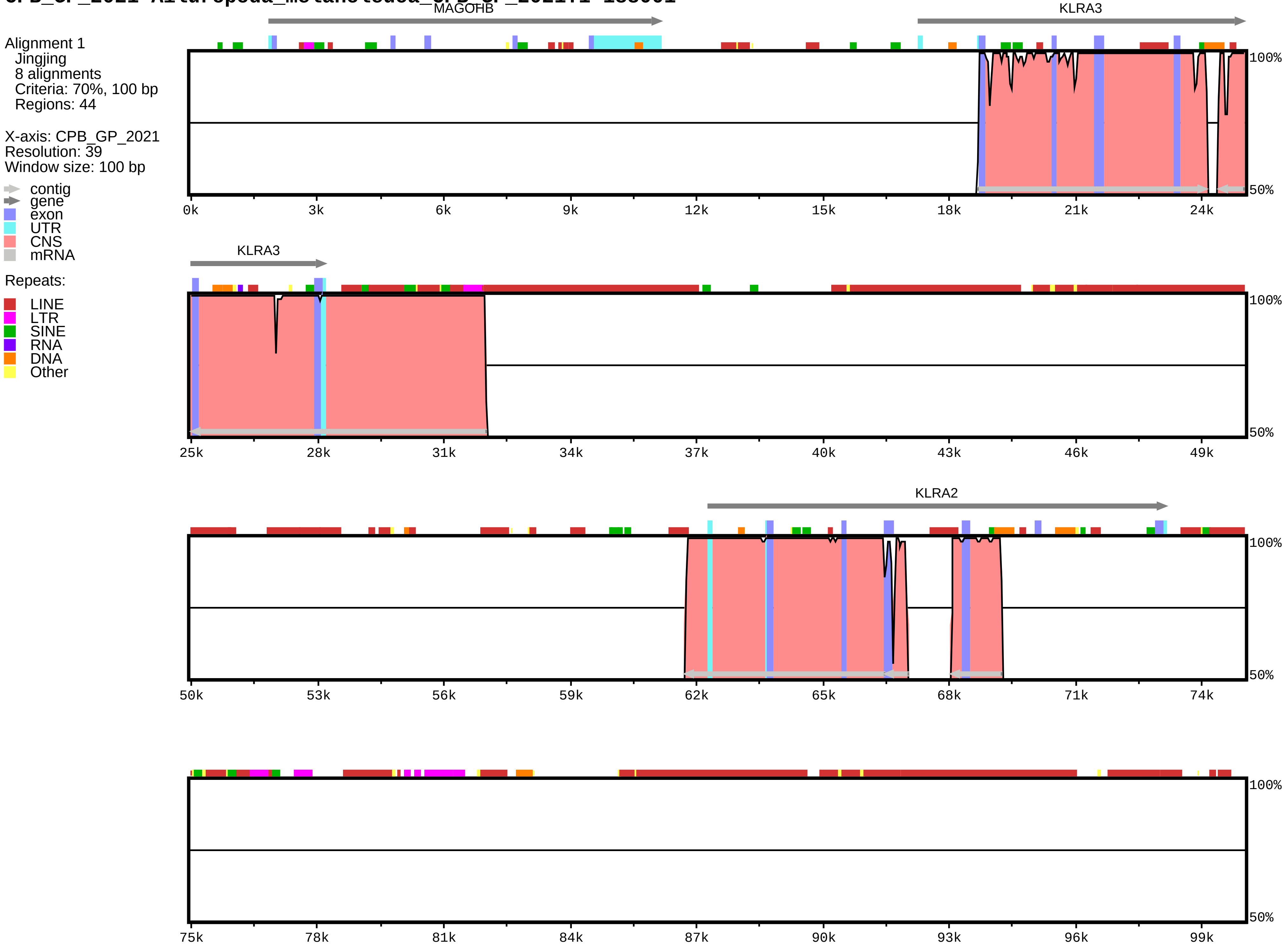

CPB_GP_2021 Ailuropoda_melanoleuca_CPB_GP_2021:1-188001

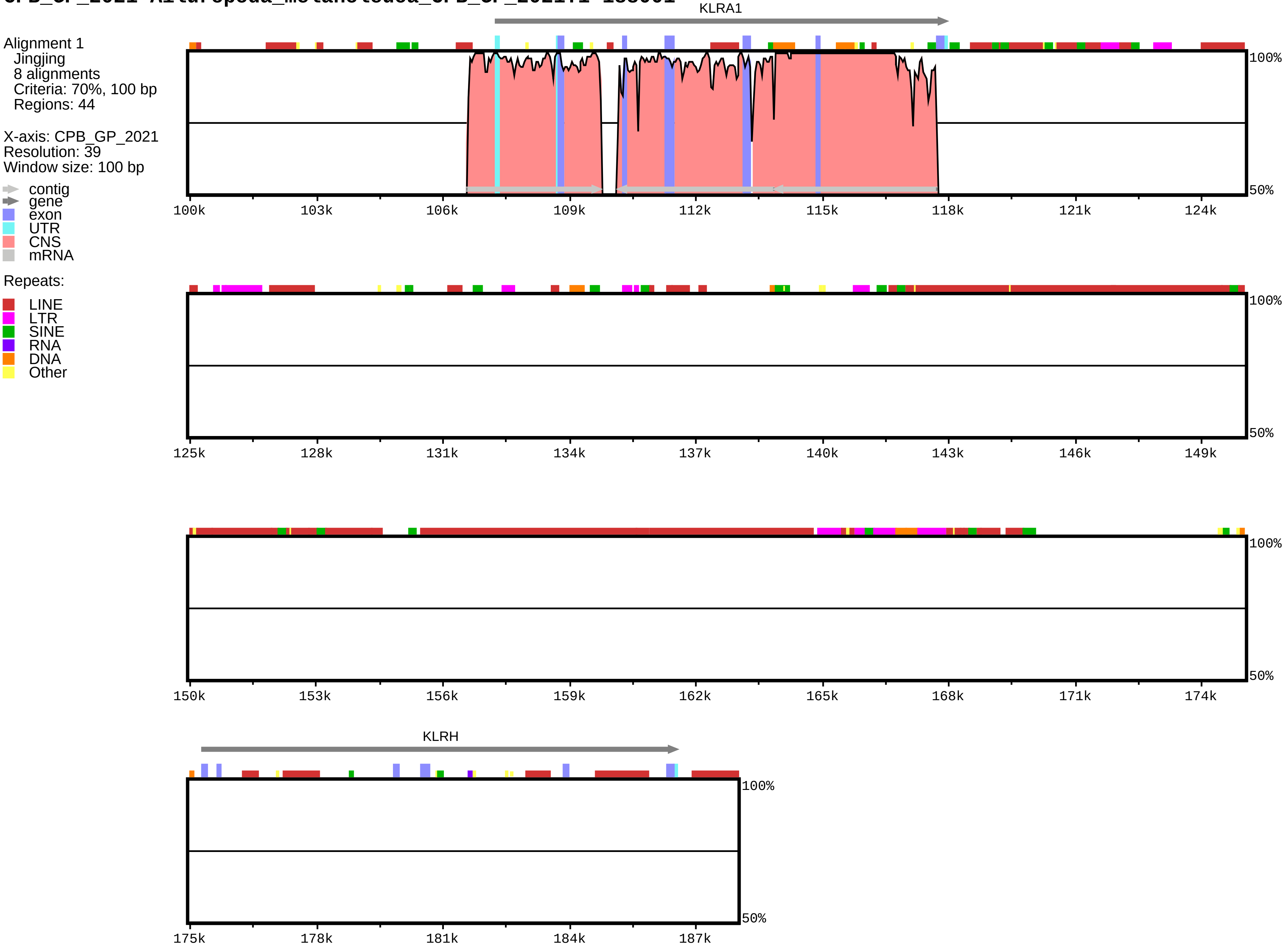

Supplement: Supplementary file 13 [file Image10.pdf]
