# Supplementary material for: Comparative genomics of the Natural Killer Complex in carnivores
Source: Front Immunol. 2024 Oct 3;15:1459122. doi: 10.3389/fimmu.2024.1459122 (PMC11484026; doi:10.3389/fimmu.2024.1459122)
Supplement: Supplementary file 16 [file Table3.pdf]

| **Felidae** | ***KLRA*** | ***KLRB*** | ***KLRC*** | ***KLRD*** | ***KLRF*** | ***KLRG*** | ***KLRH*** | ***KLRJ*** | ***KLRK*** | ***KLRL*** |
|---|---|---|---|---|---|---|---|---|---|---|
| *Acinonyx jubatus* | 1 | 1 | 7 (3) | 1 | 2 (1) | 1 | 1 (3) | 1 | 1 | 1 |
| *Caracal caracal* | 1 | 1 | 2 (5) | 1 | 0 (3) | 1 | 0 (2) | 1 | 0 (1) | 1 |
| *Felis catus* | 1 | 1 | 5-8 (0-3) | 1 | 3 | 1 | 1-4 (0-2) | 1 | 1 | 1 |
| *Felis chaus* | 1 | 1 | 7 | 1 | 3 | 1 | 5 | 1 | 1 | 1 |
| *Felis nigripes* | 1 | 1 | 6-7 (0-1) | 1 | 3 | 1 | 0-2 (0-2) | 1 | 1 | 0 (1) |
| *Leopardus geoffroyi* | 1 | 1 | 9 (2) | 1 | 2 (1) | 1 | 5 | 1 | 1 | 1 |
| *Lynx canadensis* | 0 (1) | 1 | 4 (3) | 1 | 2 (1) | 0 (1) | 5 (3) | 1 | 1 | 1 |
| *Lynx pardinus* | 0 (1) | 1 | 4 (6) | 1 | 2 (1) | 1 | 0 (6) | 1 | 1 | 1 |
| *Lynx rufus* | 1 | 1 | 8 (5) | 1 | 2 (1) | 1 | 6 (5) | 1 | 1 | 1 |
| *Neofelis diardii* | 1 | 1 | 6 (7) | 1 | 2 (1) | 1 | 0 (2) | 1 | 1 | 1 |
| *Neofelis nebulosa* | 1 | 1 | 10 (4) | 1 | 2 (1) | 1 | 0 (16) | 1 | 1 | 1 |
| *Otocolobus manul* | 1 | 1 | 4 (1) | 1 | 3 | 1 | 1 (1) | 1 | 1 | 1 |
| *Panthera leo* | 1 | 1 | 13 (2) | 1 | 2 (1) | 1 | 1 (3) | 1 | 1 | 1 |
| *Panthera onca* | 1 | 1 | 3 (9) | 1 | 1 (2) | 1 | 0 (5) | 1 | 1 | 1 |
| *Panthera pardus* | 1 | 1 | 13-16 (3-4) | 1 | 2 (1) | 1 | 2-4 (9-25) | 1-2 | 1 | 2 |
| *Panthera tigris* | 1 | 1 | 6 (2) | 1 | 2 (1) | 1 | 6 (5) | 1 | 1 | 1 |
| *Panthera uncia* | 1 | 0-1 (0-1) | 1-3 (4) | 1 | 1 (2) | 1 | 0 (1) | 1 | 1 | 1 |
| *Prionailurus bengalensis* | 1 | 1 | 6 (1) | 1 | 3 | 1 | 5 | 1 | 1 | 1 |
| *Prionailurus iriomotensis* | 1 | 1 | 1 (9) | 1 | 3 | 1 | 0 (6) | 0 (1) | 0 (1) | 1 |
| *Prionailurus viverrinus* | 1 | 1 | 6 (3) | 1 | 3 | 1 | 5 (1) | 1 | 1 | 1 |
| *Puma concolor* | 1 | 1 | 3-11 (1-2) | 1 | 1 (2) | 1 | 0-1 (2-7) | 1 | 1 | 1 |
| *Puma yagouaroundi* | 1 | 1 | 2 (4) | 0 (1) | 1 (2) | 1 | 0 (2) | 1 | 1 | 1 |

| **Canidae** | ***KLRA*** | ***KLRB*** | ***KLRC*** | ***KLRD*** | ***KLRF*** | ***KLRG*** | ***KLRH*** | ***KLRJ*** | ***KLRK*** | ***KLRL*** |
|---|---|---|---|---|---|---|---|---|---|---|
| *Canis latrans* | 1 | 1 | 2 (5) | 1 | 1 (2) | 1 | 0 (1) | 1 | 1 | 0 (1) |
| *Canis lupus* | 0-1 (0-1) | 1 | 3 (4-6) | 1 | 0-1 (2-3) | 1 | 1 | 0-1 (0-1) | 0-1 (0-1) | 0 (1) |
| *Canis lupus dingo* | 1 | 1 | 3-4 (4-5) | 1 | 1 (2) | 1 | 1 | 1 | 1 | 0 (1) |
| *Canis lupus familiaris* | 1 | 1 | 3 (4) | 1 | 1 (2) | 1 | 1 | 1 | 1 | 0 (1) |
| *Chrysocyon brachyurus* | 1 | 1 | 1 (6) | 1 | 1 (2) | 1 | 0 (2) | 1 | 1 | 0 (1) |
| *Lycaon pictus* | 1 | 1 | 1 (4) | 1 | 1 (2) | 1 | 1 | 1 | 1 | 0 (1) |
| *Nyctereutes procyonoides* | 1 | 1 | 2 (9) | 1 | 1 (2) | 1 | 1 | 1 | 1 | 0 (1) |
| *Otocyon megalotis* | 1 | 1 | 2 (3) | 1 | 1 (2) | 1 | 0 (1) | 1 | 1 | 0 (1) |
| *Speothos venaticus* | 1 | 1 | 1 (10) | 1 | 1 (2) | 1 | 1 | 1 | 1 | 0 (1) |
| *Urocyon cine-reoargenteus* | 1 | 1 | 2 (5) | 1 | 1 (2) | 1 | 0 (1) | 1 | 1 | 0 (1) |
| *Vulpes corsac* | 1 | 1 | 3 (4) | 1 | 2 (1) | 1 | 1 | 0 (1) | 1 | 0 (1) |
| *Vulpes ferrilata* | 1 | 1 | 3 (4) | 1 | 1 (2) | 1 | 1 | 0 (1) | 1 | 0 (1) |
| *Vulpes lagopus* | 1 | 1 | 2 (2) | 1 | 1 (2) | 1 | 1 | 0 (1) | 0 (1) | 0 (1) |
| *Vulpes vulpes* | 1 | 0 (1) | 3 (2) | 1 | 2 (1) | 1 | 1 | 1 | 1 | 0 (1) |

| Mustelidae | *KLRA* | *KLRB* | *KLRC* | *KLRD* | *KLRF* | *KLRG* | *KLRH* | *KLRJ* | *KLRK* | *KLRL* |
|---|---|---|---|---|---|---|---|---|---|---|
| *Eira barbara* | 0 (1) | 2 | 5 (5) | 0 (1) | 2 (1) | 1 | 0 (1) | 1 (1) | 1 | 0 (1) |
| *Enhydra lutris kenyoni* | 1 | 2 | 5 (8) | 1 | 2 | 1 | 1 | 2 | 1 | 1 |
| *Gulo gulo luscus* | 0 (1) | 2 | 6 (6) | 1 | 3 | 1 | 3 (1) | 1 (1) | 1 | 1 (2) |
| *Lontra canadensis* | 1 | 2 | 5 (7) | 1 | 2 (1) | 1 | 2 (1) | 2 | 1 | 1 (1) |
| *Lutra lutra* | 1 | 2 | 4 (6) | 1 | 1 (2) | 1 | 1 (2) | 2 | 1 | 1 (1) |
| *Martes flavigula* | 0 (1) | 1 (1) | 6 (4) | 1 | 3 | 1 | 3 (2) | 1 (1) | 1 | 1 (1) |
| *Martes martes* | 0 (1) | 2 | 6 (6) | 1 | 3 | 1 | 4 (4) | 2 | 1 | 1 (1) |
| *Martes zibellina* | 0 (1) | 0 (2) | 5 (8) | 1 | 3 | 1 | 0 (4) | 2 | 1 | 1 (1) |
| *Meles meles* | 1 | 2 | 4 (6) | 1 | 2 (1) | 1 | 2 (1) | 2 | 1 | 1 |
| *Mellivora capensis* | 0 (1) | 1 (1) | 1 (6) | 0 (1) | 0 (3) | 0 (1) | 0 (2) | 1 (1) | 0 (1) | 0 (1) |
| *Mustela erminea* | 1 | 2 | 5 (5) | 1 | 3 | 1 | 3 | 2 | 1 | 1 |
| *Mustela eversmannii* | 0 (1) | 2 | 5 (5) | 1 | 3 | 0 (2) | 3 | 2 | 1 | 1 |
| *Mustela lutreola* | 1 | 2 | 6 (5) | 1 | 3 | 0 (2) | 4 (3) | 2 | 1 | 1 |
| *Mustela nigripes* | 0 (1) | 2 | 5 (5) | 1 | 2 (1) | 0 (1) | 0 (3) | 2 | 1 | 1 (1) |
| *Mustela nivalis* | 1 | 2 | 5 (3) | 1 | 3 | 0 (1) | 1 (1) | 2 | 1 | 1 |
| *Mustela putorius furo* | 1 | 2 | 4 (6) | 1 | 3 | 1 | 4 | 2 | 1 | 1 |
| *Neogale vison* | 1 | 2 | 5 (3) | 1 | 3 | 1 | 3 | 2 | 1 | 1 (1) |
| *Pteronura brasiliensis* | 1 | 0 (2) | 1 (6) | 0 (1) | 3 | 0 (1) | 0 (2) | 1 (1) | 0 (1) | 0 (1) |
| *Taxidea taxus jeffersonii* | 1 | 1 | 0 | 0 (1) | 1 | 0 | 0 (1) | 0 | 0 | 0 |

| **Otariidae** | ***KLRA*** | ***KLRB*** | ***KLRC*** | ***KLRD*** | ***KLRF*** | ***KLRG*** | ***KLRH*** | ***KLRJ*** | ***KLRK*** | ***KLRL*** |
|---|---|---|---|---|---|---|---|---|---|---|
| *Arctocephalus towsendii* | 1 | 1 | 1 (3) | 1 | 2 | 1 | 1 (1) | 1 | 0 (1) | 0 (1) |
| *Callorhinus ursinus* | 1 | 1 | 1 (4) | 1 | 1 (1) | 1 | 2 | 1 | 1 | 0 (2) |
| *Eumetopias jubatus* | 1 | 1 | 1 (4) | 1 | 2 | 1 | 2 | 1 | 1 | 0 (2) |
| *Zalophus californianus* | 1 | 1 | 1 (4) | 1 | 2 | 1 | 2 | 1 | 1 | 0 (2) |

| **Phocidae** | ***KLRA*** | ***KLRB*** | ***KLRC*** | ***KLRD*** | ***KLRF*** | ***KLRG*** | ***KLRH*** | ***KLRJ*** | ***KLRK*** | ***KLRL*** |
|---|---|---|---|---|---|---|---|---|---|---|
| *Halichoerus grypus* | 1 | 1 | 1 (3) | 1 | 2 | 1 | 1 (3) | 1 | 1 | 0 (3) |
| *Leptonychotes weddellii* | 1 | 1 | 1 (3) | 0 (1) | 1 (1) | 1 | 2 (2) | 1 | 1 | 0 (3) |
| *Mirounga angustirostris* | 1 | 0-1 (0-1) | 1 (3) | 1 | 2 | 1 | 1-2 (1-2) | 0-1 (0-1) | 1 | 0 (3) |
| *Mirounga leonina* | 1 | 1 | 1 (3) | 1 | 2 | 1 | 2 (1) | 1 | 1 | 0 (3) |
| *Neomonachus schauinslandi* | 1 | 1 | 1 (3) | 1 | 1 (1) | 1 | 3 (1) | 1 | 1 | 0 (3) |
| *Phoca vitulina* | 1 | 1 | 1 (3) | 1 | 2 | 1 | 3 (2) | 1 | 1 | 0 (3) |
| *Pusa hispida saimensis* | 1 | 1 | 2 (3) | 1 | 2 | 1 | 3 (1) | 1 | 1 | 0 (4) |
| *Pusa sibirica* | 1 | 1 | 0 (4) | 1 | 2 | 1 | 2 (2) | 1 | 1 | 0 (3) |

| **Ursidae** | ***KLRA*** | ***KLRB*** | ***KLRC*** | ***KLRD*** | ***KLRF*** | ***KLRG*** | ***KLRH*** | ***KLRJ*** | ***KLRK*** | ***KLRL*** |
|---|---|---|---|---|---|---|---|---|---|---|
| *Ailuropoda melanoleuca* | 1-2 (0-1) | 1 | 4 (2) | 1 | 0-1 (2-3) | 0-1 (0-1) | 1 | 1 | 1 | 0-1 (1-2) |
| *Helarctos malayanus* | 0 | 1 | 3 (3) | 0 | 1 (2) | 1 | 0 (3) | 1 | 1 | 1 (3) |
| *Tremarctos ornatus* | 0 (1) | 1 | 4 (2) | 1 | 1 (2) | 1 | 1 (2) | 1 | 1 | 1 (3) |
| *Ursus americanus* | 2 | 1 | 3 (3) | 1 | 1 (2) | 1 | 2 (2) | 1 | 1 | 1 (4) |
| *Ursus arctos* | 2 | 1 | 3 (3) | 1 | 1 (2) | 1 | 3 (1) | 1 | 1 | 1 (4) |
| *Ursus maritimus* | 2 | 1 | 3 (3) | 1 | 1 (2) | 1 | 3 (1) | 1 | 1 | 1 (4) |
